# Plasmid Flux in *Escherichia coli* ST131 Sublineages, Analyzed by Plasmid Constellation Network (PLACNET), a New Method for Plasmid Reconstruction from Whole Genome Sequences

**DOI:** 10.1371/journal.pgen.1004766

**Published:** 2014-12-18

**Authors:** Val F. Lanza, María de Toro, M. Pilar Garcillán-Barcia, Azucena Mora, Jorge Blanco, Teresa M. Coque, Fernando de la Cruz

**Affiliations:** 1 Departamento de Biología Molecular (Universidad de Cantabria) and Instituto de Biomedicina y Biotecnología de Cantabria IBBTEC (UC-SODERCAN-CSIC), Santander, Spain; 2 Laboratorio de Referencia de *E. coli* (LREC), Departamento de Microbiología y Parasitología, Facultad de Veterinaria, Universidad de Santiago de Compostela, Lugo, Spain; 3 Departamento de Microbiología, Hospital Universitario Ramón y Cajal, Instituto Ramón y Cajal de Investigación Sanitaria (IRYCIS), Madrid, Spain; 4 Unidad de Resistencia a Antibióticos y Virulencia Bacteriana asociada al Consejo Superior de Investigaciones Científicas (CSIC), Madrid, Spain; 5 Centros de Investigación Biomédica en Red de Epidemiología y Salud Pública, (CIBER-ESP), Madrid, Spain; MicroTrek Incorporated, United States of America

## Abstract

Bacterial whole genome sequence (WGS) methods are rapidly overtaking classical sequence analysis. Many bacterial sequencing projects focus on mobilome changes, since macroevolutionary events, such as the acquisition or loss of mobile genetic elements, mainly plasmids, play essential roles in adaptive evolution. Existing WGS analysis protocols do not assort contigs between plasmids and the main chromosome, thus hampering full analysis of plasmid sequences. We developed a method (called plasmid constellation networks or PLACNET) that identifies, visualizes and analyzes plasmids in WGS projects by creating a network of contig interactions, thus allowing comprehensive plasmid analysis within WGS datasets. The workflow of the method is based on three types of data: assembly information (including scaffold links and coverage), comparison to reference sequences and plasmid-diagnostic sequence features. The resulting network is pruned by expert analysis, to eliminate confounding data, and implemented in a Cytoscape-based graphic representation. To demonstrate PLACNET sensitivity and efficacy, the plasmidome of the *Escherichia coli* lineage ST131 was analyzed. ST131 is a globally spread clonal group of extraintestinal pathogenic *E. coli* (ExPEC), comprising different sublineages with ability to acquire and spread antibiotic resistance and virulence genes via plasmids. Results show that plasmids flux in the evolution of this lineage, which is wide open for plasmid exchange. MOB_F12_/IncF plasmids were pervasive, adding just by themselves more than 350 protein families to the ST131 pangenome. Nearly 50% of the most frequent γ–proteobacterial plasmid groups were found to be present in our limited sample of ten analyzed ST131 genomes, which represent the main ST131 sublineages.

## Author Summary

Plasmids are difficult to analyze in WGS datasets, due to the fragmented nature of the obtained sequences. We developed a method, called PLACNET, which greatly facilitates this analysis. As an example, we analyzed the plasmidome of *E. coli* ST131, an ExPEC clonal group involved in human urinary tract infections and septicemia. Relevant variation within this clone (e.g., antibiotic resistance and virulence) is frequently caused by the acquisition and loss of plasmids and other mobile genetic elements. Nevertheless, our knowledge of the ST131 plasmidome is limited to a few antibiotic resistance plasmids and to identification of replicons from known plasmid groups. PLACNET analysis extends the number of sequenced plasmids in ST131, which can be used for comparative genomics, from 11 to 50. The ST131 plasmidome is seemingly huge, encompassing roughly 50% of the main plasmid groups of γ–proteobacteria. MOB_F12_/IncF plasmids are apparently the most active players in the dissemination of relevant genetic information.

## Introduction

Clinical microbiology is being transformed by whole genome sequencing (WGS) [1]. A case in point is *Escherichia coli*: there were 1,618 *E. coli* projects submitted to NCBI compared to just 68 complete genomes by year 2013. Within the realms of clinical and environmental microbiology, plasmid analysis is increasingly used to track the dissemination of genes encoding virulence, resistance to antibiotics, heavy metals and biocides [2]–[4] and, to a lesser extent, to analyze differences in the adaptive evolution of certain clonal backgrounds [5], [6]. Hybridization with specific probes [7], amplification of plasmid replication initiator proteins (RIP) [8]–[10], and relaxases (REL) [11] allow preliminary identification of plasmid families. In addition, plasmid MLST (pMLST) is used for epidemiological surveillance, but is restricted to individual plasmids of a few plasmid families of Enterobacteriaceae (http://pubmlst.org/plasmid/). This precludes the detection of plasmid mutations or rearrangements, as well as the identification of conjugative plasmids not represented in the pMLST database and of most mobilizable plasmids [11]. Finished plasmid/genome sequencing provides accurate and non-biased information, but is still expensive and thus seldom used specifically for plasmid analysis. Draft WGS dramatically cut down cost and analysis time. Although it allowed rapid and cheap data acquisition, WGS datasets typically result in more than a hundred contigs for a given genome, due to the short read lengths generally obtained. Genome fragmentation makes it difficult to distinguish between physical units, that is, between chromosome and plasmid sequences, as well as between different plasmids that usually coexist in bacterial cells. Several strategies can be followed to analyze WGS genome sequences, the workflow described by [12] being a typical example. There are also applications to identify plasmids in WGS sequences, such as PlasmidFinder (http://cge.cbs.dtu.dk/services/PlasmidFinder/), which identifies plasmids according to PCR-based replicon typing (PBRT) [8]–[10] and the subtyping scheme included in the pMLST web page (http://pubmlst.org). PlasmidFinder is limited by its inability to reconstruct the sequences of entire plasmids, underscoring the urgent need for improvement over existing tools.

*E. coli* ST131 is a successful high-risk clonal complex of pandemic distribution, able to cause extraintestinal infections in humans [13]-[18]. The increasing recovery of ST131 isolates from hospitalized and non-hospitalized individuals and, more recently, from companion and foodborne animals [17], [19]–[25], sewage and main rivers of large European cities [26], [27] highlights the rapid spread and local adaptation to different habitats of this lineage. ST131 is characterized by high metabolic potential [28] and a variable number of virulence factors, including adhesins, siderophores, toxins, polysaccharide coats (capsules and lipopolysaccharides), protectins and invasins [19], [29], [30], mostly acquired by recombination and by the interplay of mobile genetic elements (MGEs) [16]. Such traits, which are common among different lineages of the *E. coli* B2 phylogroup [31], [32], enable strains to colonize mucosal surfaces, invade tissues, foil defence mechanisms and yield injurious inflammatory responses in the host. *E. coli* populations identified as ST131 by the widely used ‘Achtman scheme’ of multilocus sequence typing (MLST) [33] (http://mlst.warwick.ac.uk/mlst/dbs/Ecoli), split in diverse clusters or subclones on the basis of genomic profile, serotype, content of virulence factors, antibiotic susceptibility pattern and the presence of certain *fimH* alleles [21], [29], [34]–[36]. The most prevalent ST131 clonal sublineage (*H*30) is characterized by the presence of a *fimH*30 allele, serotype O25:H4 and a specifically conserved *gyrA*/*parC* allele combination that confers fluoroquinolone resistance (FQ-R). Most human infections caused by ST131 are due to isolates of the *H*30 sublineage [13], [16], [37]–[39], many of them carrying the *bla*_CTX-M-15_ gene which is responsible for resistance to third generation of cephalosporins. Some authors suggested differences between CTX-M-15 and non-CTX-M-15 producers, referred to as *H*30*-*R and *H*30-Rx sublineages, respectively [13], [35], [37], [38]. Currently, diverse O25b:H4 ST131 variants (e.g. *fimH*22, *fimH*30) or O16:H5 (e.g. *fimH*41) seem also to be widely spread [13]-[16], [40]. Full genome sequencing of several ST131 *E. coli* genomes, most of them *H*30-Rx variants [16], [41]–[44], revealed further differences among strains, mainly chromosomal SNPs, indels and plasmid variations [16], [43], [44]. Heterogeneity of MGEs has been reported in other relevant *E. coli* clones, mainly Shiga-toxin producing *E. coli* (STEC) as O157:H7, O104:H4 or O26:H11 [5], [6], [45]–[47], often associated with ecological diversification of *E. coli* populations that can influence host-pathogen interactions [48], [49]. Recently, International and European organisations including European Food Safety Agency, EFSA; European Centre for Disease Control, ECDC; Food Drug Administration, FDA; Centre for Diseases Control, CDC) and national food safety authorities underscored the need to identify clonal variants with enhanced transmissibility or pathogenicity as well as to infer the evolutionary history of pathogens of interest in Public Health (http://www.efsa.europa.eu/en/events/event/140616.htm). Because relevant adaptive traits are plasmid located, there is an urgent need to consider MGEs in population genetic studies.

In this work we describe PLACNET, a method to reconstruct plasmids from WGS datasets, and its application to the comprehensive analysis of bacterial plasmidomes. As a specific example, we describe the ST131 plasmidome and discuss its possible impact in the diversification of this clinically important lineage. PLACNET allows the identification of plasmids currently circulating among *E. coli* and other enterobacterial species that may be underestimated, thus providing a useful tool to approach comprehensive plasmid population genetic studies.

## Results

### Phylogeny of *E. coli* ST131 genomes

**Figure 1:**
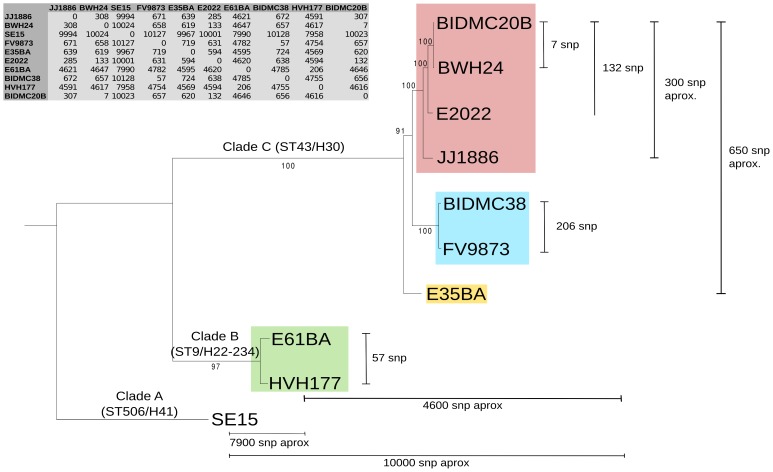
Phylogenetic tree of ST131 *E. coli*. The tree is based on a 3,629,034 bp core genome (3,734 orthologous genes: 90% identity and 90% coverage) and 100 bootstrapping replicates. ST131 clades are named according to [16] and further subdivided and colored according to virotypes [36]: virotype A (blue), virotype B (yellow), virotype C (pink), virotype D (green). Virotype classification is based on the presence/absence of four putative virulence factors: *afaFM955459* (encoding an Afa/Dr adhesin), *sat* (secreted autotransporter toxin, present in PAI-CFT073-pheV), *ibeA* (invasion of brain endothelium) and *iroN* (salmochelin siderophore receptor). The commensal ST131 strain SE15 was used to root the tree (virotype non typable; serotype O150 in the original publication [91] but lying within the H41 cluster in the phylogenomic study of [16]). Given SNP numbers are approximate averages of individual comparisons.

We analyzed ten *E. coli* genomes, classified as ST131 according to the Achtman scheme (http://mlst.warwick.ac.uk/mlst/dbs/Ecoli), which branch in three main clusters identified as ST43, ST9 and ST506 (Fig. 1) according to the cgMLST Pasteur Institute scheme (http://www.pasteur.fr/recherche/genopole/PF8/mlst/EColi.html). The use of these two schemes is widely accepted in epidemiology [50] and increasingly used for *E. coli* typing. The ST43 branch contains isolates of the *H*30 lineage, which split in three subclusters (four strains of virotype C, two of virotype A, one of virotype B). The ST9 branch corresponds to isolates of the *H*22/*H*324 sublineage (virotype D). The most distal branch to the main cluster is represented by the commensal strain SE15, a member of sublineage *H*41 identified as ST506 [16]. It does not contain any marker used for the virotype subtyping method described by Blanco et al (*afa, sat, ibeA, iroN*) [36], [51], [52]. Thus, the sample analyzed in this work includes representatives of all ST131 branches described to date [13], [16]. The core genome of the 10 strains encompasses 3.6 Mb (Fig. 1 inset). As can be seen, the phylogenetic tree of ST131 genomes can be rooted at the commensal strain SE15. It should be noted, however, that SE15 is not necessarily the ancestor of the pathogenic lineages, as inferred by recent evidence [16]. The divergence of SE15 from the other ST131 strains is of about 3,000 SNP/Mb, a measure of the depth of the ST131 phylogenetic branch (<0.3% divergence in the core genome). There are only 650 SNPs among the genomes of cluster C lineage (i.e., <200 SNP/Mb), indicating their close phylogenetic relationship. There are <300 SNPs within a given virotype. The average distance between clades A and B is of about 4,600 SNPs (i.e., 1,300 SNP/Mb).

### Plasmid reconstruction in *E. coli* ST131 genomes

**Figure 2:**
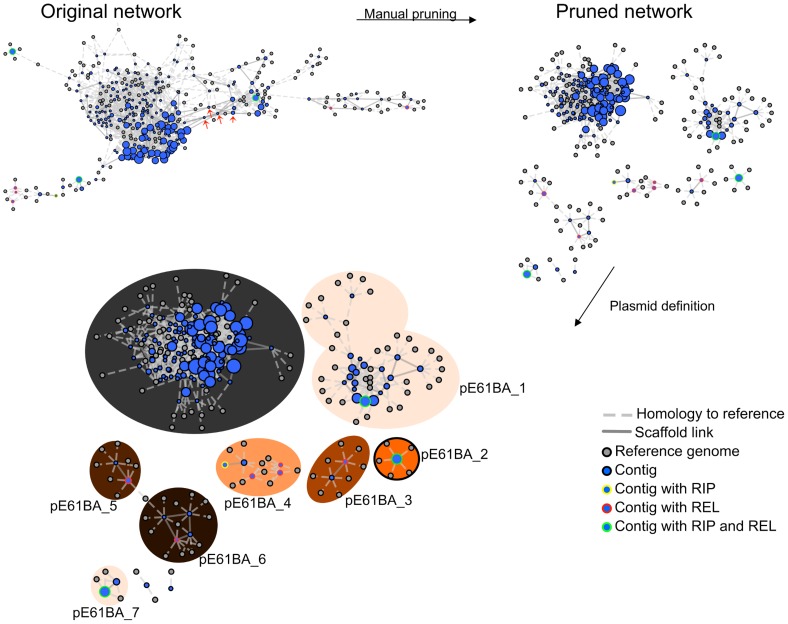
PLACNET plasmid reconstruction of ST131 genome E61BA (ST9/H324/virotype D). The network contains nodes of two different colors (blue for contigs, grey for reference genomes). The size of reference nodes is always the same. The size of the contig nodes is proportional to the contig length. Besides, outlines are yellow for contigs containing RIP proteins, red for relaxases and green for both proteins. Edges are either solid (scaffold links) of dotted (homologous references). The length of the edges is arbitrarily selected by Cytoscape algorithm. In the upper left, the network output (original network) is shown, which resulted from automatic reference search, scaffold links and protein tagging rules. The original network was converted to a pruned network by eliminating contigs smaller than 200 bp and duplicating specific hubs (red arrows). Two contigs could not be assigned for lack of scaffold links: a 2,953 bp contig (putative DNA primase + lytic transglycosylase) and a 1,301 bp contig (TrbI + TraB-partial). Closed plasmids (e.g., pE61BA_2, size: 24,447 bp) are shown with a black outline in the final PLACNET network.

The PLACNET protocol was used as explained in Materials and Methods. We proceeded with plasmid reconstruction, as exemplified in Fig. 2 for the reconstruction of the E61BA genome (ST9/H324/virotype D). When we applied the rules for reference homology, scaffold links and plasmid protein tagging, the E61BA network shown as “original network” was produced. Obviously, this network was not neat enough to allow plasmid reconstruction. Expert pruning of the network consisted on several steps. First, contigs smaller than 200 bp were eliminated. Second, hubs were identified (see arrows in the original network of Fig. 2), duplicated and assigned to separate disjoint connected components. Scaffold links and coverage information, as well as score values of conflict edges, were used to decide on valid component assignment. Inspection of the coding potential of hubs usually showed them to correspond to ISs, transposons or other known repeated elements (as shown in S9 and S10 Figs.). As a result, a pruned network was reconstructed as shown in Fig. 2. Differential coloring of disjoint connected components in the pruned network thus displayed the final network of plasmids (as contig constellations). In PLACNET Cytoscape representation, most plasmids can be identified by their RIP and/or REL proteins. Thus, the reconstructed E61BA genome contains seven plasmids: a 134 kb MOB_F12_/IncF plasmid (pE61BA-1), a 37.7 kb MOB_P6_/IncI2 plasmid (pE61BA-7), a 24.5 kb MOB_C12_ plasmid (pE61BA-2), a 18 kb MOB_P11_/IncP1 plasmid (pE61BA-4), two MOB_P5_/ColE1-like plasmids of 6.6 and 6.9 kb (pE61BA-5 and pE61BA-6, respectively) and one MOB_Q12_ 5.0 kb plasmid (pE61BA-3). Only plasmid pE61BA-2 could be closed, the remaining contained at least two contigs. Thus, their reported sizes are minimum sizes, since they might include small repeated sequences that were taken out of the analysis during network pruning. Two contigs remained as “not assigned” to any physical unit in this particular genome because they did not show any reference or scaffold link that bind them to other contigs: a 2,953 bp contig (containing a putative DNA primase and a lytic transglycosylase) and a 1,301 bp contig (containing two conjugation-related genes: *trbI* and a partial *traB* gene).

The same procedure was applied to the three other strains sequenced for this work as well as to the four genomes obtained from public DBs as Illumina reads. The plasmid content of the four strains sequenced in this work was confirmed by the analysis of S1-digested genomic DNA profiles by PFGE. This analysis fully confirmed the presence of plasmids of similar size to those identified by PLACNET (S2 Text and S4 Table), In the case of strain E35BA, in which PLACNET identified two IncF plasmids that could not be separated (totaling 211 kb), S1-PFGE identified two plasmids of 140 kb and 75 kb. As a result of PLACNET analysis, we obtained the plasmid constellation networks shown in S1 to S8 Figs. A summary of the results, i.e., the reconstructed plasmids, is shown in Table 1, which includes also the plasmids of the ST131 reference strains JJ1886 and SE15. As can be seen, the number of plasmids in the ST131 genomes is variable, even from strains belonging to the same ST131 sublineage, ranging from just one plasmid in HVH177 (clade B/ST9/*fimH22*) or SE15 (clade A/ST506/*fimH41*) to seven plasmids in E61BA (clade B/ST9/*fimH22*), to give an average of 4 plasmids per genome. There is not a single plasmid group that appears specific of a particular sublineage. S1 Table contains the complete list of contigs assigned to each plasmid or chromosome.

**Table 1:** Summary of plasmid content.

Genome	*fimH* allele	Strain virotype	MOB_F12_/IncF	Phage-related/RepFIB	MOB_P12_/IncI	MOB_P6_/IncI2	MOB_F11_/IncN	MOB_P3_/IncX	MOB_P11_/IncP1	MOB_C12_ d	MOB_P5_/ColE1-like	MOB_Qu_ d	MOB_Q12_ d	no-MOB^d^
**FV9873**	*H*30	A	pFV9873_5 (91.4 Kb; ΔTraI)					pFV9873_4 (33.3 Kb)				pFV9873_1 (4.1 Kb)	pFV9873_6 (5.2 Kb)	pFV9873_2 (2.2 Kb); pFV9873_3 (4.6 Kb)
**BIDMC38**	*H*30	A	pBIDMC38_5 (123 Kb)								pBIDMC38_1 (11.8 Kb)	pBIDMC38_4 (4.2 Kb)	pBIDMC38_2 (5.3 Kb)	pBIDMC38_3 (1.6 Kb)
**E35BA**	*H*30	B	p35BA_2+3 (211 Kb)						IME_E35BA (14.2 Kb)			pE35BA_1 (4.1 Kb)		
**E2022**	*H*30	C	pE2022_2 (103 Kb)		pE2022_1 (98.3 Kb)			pE2022_3 (35.0 Kb)				pE2022_4 (4.1 Kb)		pE2022_5 (2.2 Kb)
**BIDMC20B**	*H*30	C	pBIDMC20B_1 (128 Kb, ΔTraI^b^)	pBIDMC20B_2 (109 Kb)										
**BWH24**	*H*30	C	pBWH24_1 (123 Kb, ΔTraI^b^)	pBWH24_2 (109 Kb)		pBWH24_3 (60.3 Kb)								
**JJ1886**	*H*30	C	pJJ1886-5 (110 Kb ΔTraI)						pJJ1886-4 (55.9 Kb)		pJJ1886-3 (5.6 Kb)		pJJ1886-2 (5.2 Kb)	pJJ1886-1 (1.6 Kb)
**E61BA**	*H*324	D	pE61BA_1 (137 Kb)			pE61BA_7 (37.9 Kb)			pE61BA_4 (18.3 Kb)	pE61BA_2 (24.5 Kb)	pE61BA_5 (6.5 Kb); pE61BA_6 (6.9 Kb)		pE61BA_3 (5.5 Kb)	
**HVH177**	*H*22	D	pHVH177_1 (78.6 Kb)											
**SE15**	*H*41	Commensal	pECSF1 (122 Kb)											
**Other ST131 plasmids** a			pEK516 (64.5 Kb, ΔTra); pEK499 (117 Kb, ΔTra); pJIE186-2^c^ (138 Kb); pGUE-NDM (87.0 Kb)		pEK204 (93.7 Kb)		pKC394 (53.2 Kb); pKC396 (44.2 Kb); pNDM-ECS01 (41.2 Kb); pECN580 (64.9 Kb)	pJIE143 (34.3 Kb)						

a Plasmid references: pEK516 ([93]; EU935738); pEK499 ([93]; EU935739); pJIE186-2 ([56]; NC_020271); pGUE-NDM (in [119]; JQ364967); pEK204 ([93]; EU935740); pKC394 ([120]; HM138652); pKC396 ([120], HM138653); pNDM-ECS01 (Unpublished; KJ413946); pECN580 ([121]; KF914891); pJIE143 ([94]; JN194214).

b pBIDMC20B_1 and pBWH24_1 plasmids lacked the REL domain of the TrwC protein.

c Plasmid pJIE186-2 was isolated from strain JIE186 [94], although GenBank acc. n° NC_020271 specifies it is located at EC958 strain. Strain JIE186 also contains plasmid pJIE186-1, not included in this study as it is not available at public DBs.

d No correlation with RIP typing methods.

#### Overall plasmid diversity is visualized in plasmid dendrograms

**Figure 3:**
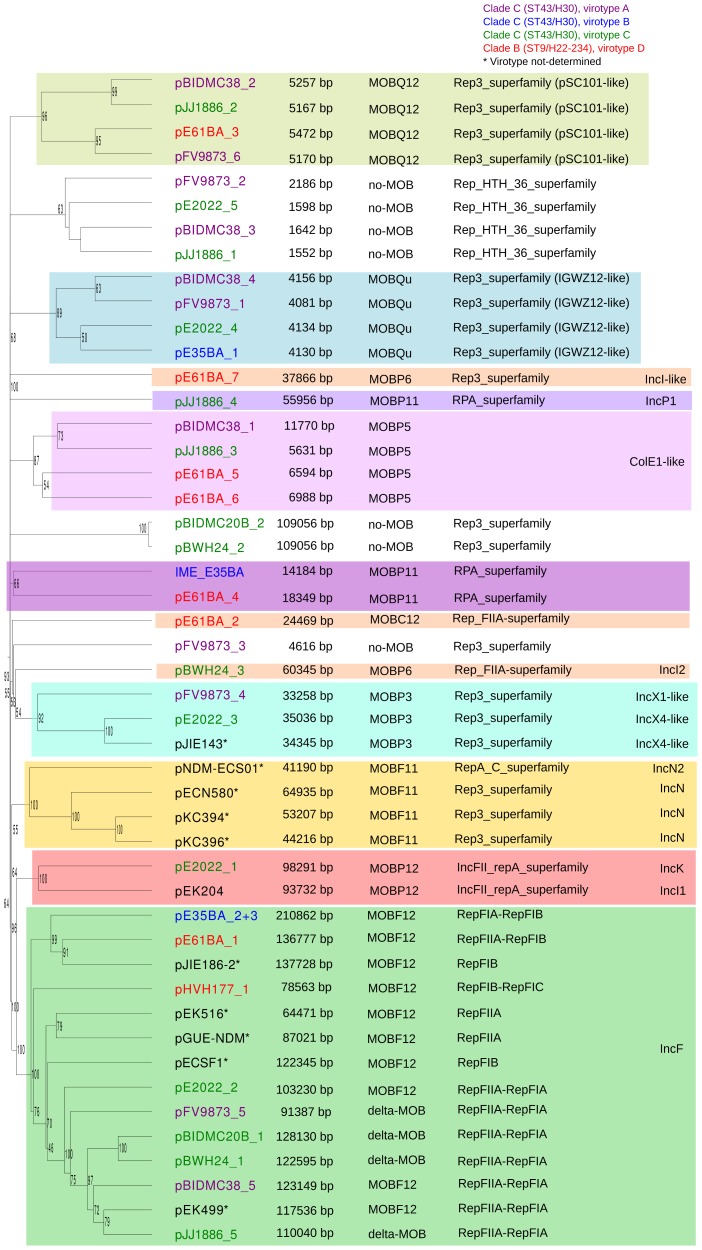
Hierarchical clustering dendrogram of ST131 plasmids. The UPGMA dendrogram was based on protein cluster analysis using 60% sequence identity and 80% coverage. Plasmid names are colored according to their clade, taking into account ST, *fimH* allele and virotype, following the color code shown at the upper right. The five plasmid names in black correspond to previously sequenced plasmids from ST131 strains. Different color backgrounds are shown to emphasize branches of related plasmids. To the right of the dendrogram, four columns show, respectively, plasmid size, MOB type, RIP type and Inc type.

**Figure 4:**
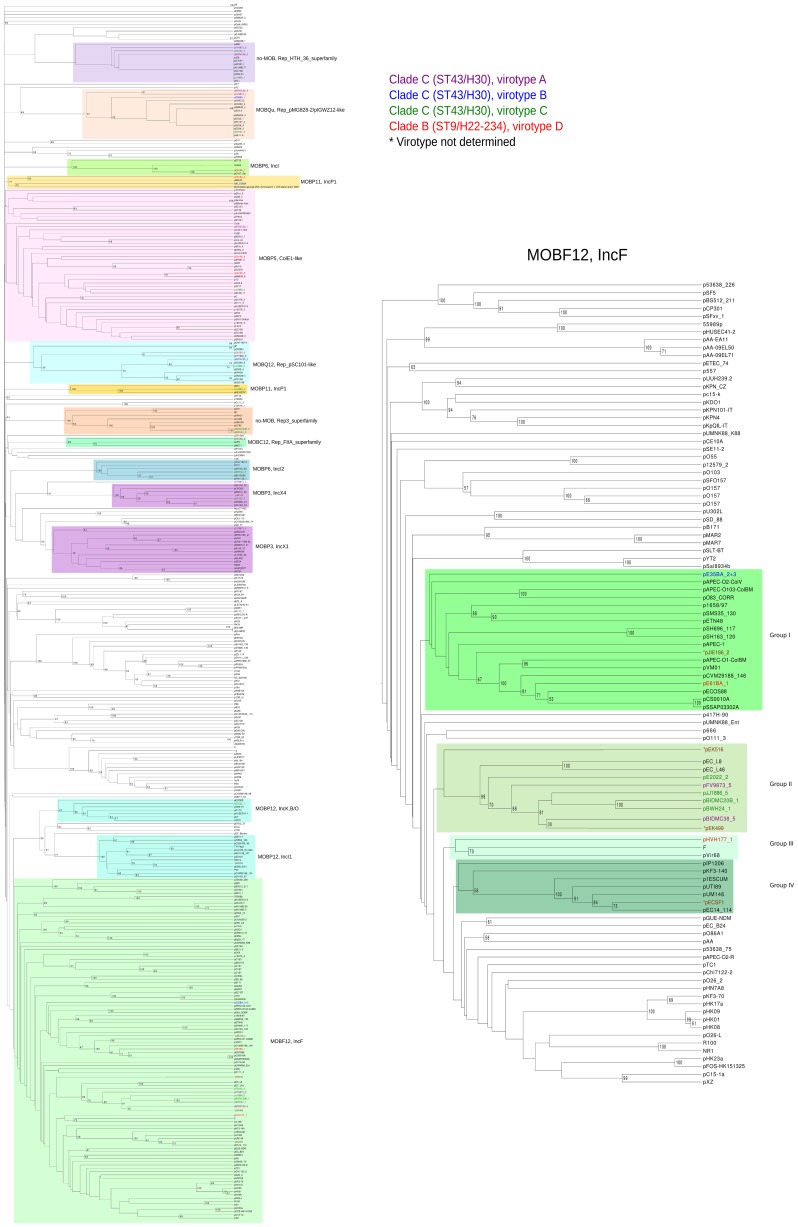
Hierarchical clustering dendrogram of ST131 plasmids and relevant references. The left dendrogram shows the complete tree, with references. Dendrogram construction and color codes are as in Fig. 4. The right dendrogram expands the MOB_F12_/IncF branch, with new background colors highlighting plasmid groups within this branch that are mentioned in the text.

Overall, the ten ST131 genomes analyzed contain 39 plasmids (including one potential ICE), which can be assorted by their relative sizes and MOB groups [53], as shown in Table 1. The most conspicuous group was that of MOB_F12_/IncF plasmids (11 plasmids), present in all ten sequenced ST131 genomes. Other relevant plasmid backbones belong to the MOB_P_ (RIP groups IncI1/K, IncI2, IncX1, IncX4, ColE1), MOB_Q_ (Qu, Q12) and MOB_C_ (C12) REL families. The non-F plasmids comprise a total of 20 plasmids belonging to eight plasmid groups. Two plasmids were phage-like and belong to the Rep-3 RIP family. Finally, 5 plasmids corresponded to the no-MOB category. The E35BA genome (ST43/H30 virotype B) showed a MOB_P11_ relaxase within a 234 kb chromosomal contig, implying the presence of an ICE (integrative and conjugative element). Detailed inspection of this contig identified a 14.2 kb IME (integrative and mobilizable element) (see below and Suppl. Mat.). Once plasmids were identified by PLACNET and contigs assorted, the next step in plasmid analysis consisted in the construction of a dendrogram that summarizes plasmid gene content, allowing a visualization of relatedness between individual plasmids. The dendrogram of the 44 plasmids found in ST131 genomes (39 described in this report plus five plasmids already published) is shown in Fig. 3. The figure shows how the plasmids divide into branches that coincide with backbone MOB groups. There are 14 plasmid groups, according to the dendrogram, shown in the figure by different color backgrounds. Since each dendrogram group links related plasmids, they can be now analyzed individually, by comparing them either among themselves (Fig. 3) or with selected references (Fig. 4).

#### Analysis of IncF plasmids

**Figure 5:**
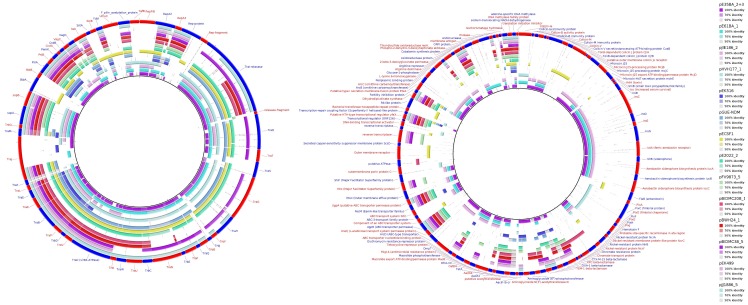
MOB_F12_/IncF plasmid analysis. Protein cluster analysis was performed with kClust software (parameters: 30% identity, 50% coverage) on the set of 14 plasmids shown in Table 4. Plasmid pGUE-NDM [119] was excluded from this comparison since it is only distantly related to the others (see dendrogram in Fig. 5). A total of 354 protein clusters were obtained and annotated versus the NCBI protein database (Blastp). Manual inspection was carried out to classify the reference proteins of each cluster into one of these three groups (comparative analysis shown with BRIG): (i) Backbone and metabolic proteins (panel A); (ii) Virulence and Antibiotic resistance proteins (panel B); and (iii) ISs and hypothetical proteins (not shown).

Representatives of the largest plasmid group, formed by 15 MOB_F12_/IncF plasmids, were found in each of the analyzed ST131 strains (Table 1). Included in this set are four plasmids lacking MOB and *tra* regions but containing RIP and other backbone genes related to IncF plasmids. The dendrogram of Fig. 3 clearly indicates that these plasmids belong to the IncF plasmid family, underscoring the usefulness of this step in plasmid reconstruction. Judging from their position in the dendrogram, it seems IncF plasmid genes are scrambled as if the precise constitution of each individual IncF plasmid could not be predicted at all for isolates of each specific ST131 cluster or phylotype. This is even clearer in Fig. 4. In this figure, ST131 plasmids are represented together with the reference sequences that were used for PLACNET reconstruction and analysis. Besides, BRIG comparison of IncF plasmids (Fig. 5) reveals high heterogeneity between them, with not a single completely conserved gene (confirmed by the fact that not a single plasmid gene was found to belong to the ST131 core genome). KClust software [54] at 30% identity and 50% coverage was used to group all proteins coded by IncF plasmids into 354 reference clusters. Manual curation was used to classify these 354 clusters in three groups (Fig. 5): (i) plasmid backbone (i.e., conjugation, RIP and maintenance genes) and metabolic protein genes, (ii) antibiotic resistance and virulence genes, and (iii) other protein genes such as ISs, transposases or hypothetical proteins. Conjugation proteins represent 30 of the 53 backbone proteins and constitute the most conserved set. As mentioned above, 4/14 plasmids do not keep a complete backbone. Table 2 contains the functional annotation of the IncF plasmids. As shown, 11/15 of the MOB_F12_/IncF plasmids contain antibiotic resistance genes (conferring resistance to beta-lactams, in all cases, but also to sulfonamides, aminoglycosides, trimethoprim, chloramphenicol, tetracycline and macrolides, in some of them). In addition, nine of the ten antibiotic resistance-plasmids confer a multidrug-resistance (MDR) phenotype (equal or more than four antibiotic families). Besides, 10/15 MOB_F12_/IncF plasmids presented putativevirulence genes (Table 2). As previously noted, there was an apparent trade-off between antibiotic resistance and virulence, genes coding for these adaptive traits being located in different plasmids [2]. Finally, a DNA modification gene (adenine-specific DNA methylase) was conserved in all IncF plasmids except in pHVH177_1.

**Table 2:** Resistance genes and virulence determinants in MOB_F12_ plasmids.

Plasmid	Strain Virotype	Size (Kb)	RIP (FAB formula)^a^	Antibiotic resistance genes^b^	Virulence genes^c^
pFV9873_5	A	91.4	RepFIIA-RepFIA (F2:A1:B-)	*bla*_CTX-M-15_, *bla*_OXA-1_, *sul1*, *aadA5*, *aac*(6′)-Ib-cr, *dfr*A17, *cat*B4, *tet*(A), *mphA*	None-detected
pBIDMC38_5	A	123	RepFIIA-RepFIA (F2:A1:B-)	*bla*_TEM-1_, *sul1*, *aadA5*, *tet*(A), *mphA*	*traT, finO*
pE2022_2	C	103	RepFIIA-RepFIA (F2:A1:B-)	*bla*_CTX-M-15_, *bla*_TEM-1_, *bla*_OXA-1_, *aac*(6′)-Ib-cr-like^d^, *cat*B4, *tet*(A)	*traT, finO*
pBIDMC20B_1	C	128	RepFIIA-RepFIA (F2:A1:B-)	*bla*_KPC-3_, *sul1, sul2, strA, strB, aadA5, dfr*A17, *tet*(A)	*finO*
pBWH24_1	C	123	RepFIIA-RepFIA (F2:A1:B-)	*bla*_KPC-3_, *sul1, sul2, strA, strB, aadA5, dfr*A17, *tet*(A)	*finO*
pJJ1886-5	C	110	RepFIIA-RepFIA (F2:A1:B-)	*bla*_TEM-1_, *bla*_OXA-1_, *aac*(6′)-Ib-cr	None-detected
pEK499	-	117	RepFIIA-RepFIA (F2:A1:B-)	*bla*_CTX-M-15_, *bla*_TEM-1_, *bla*_OXA-1_, *sul1*, *aadA5*, *aac*(6′)-Ib-cr, *dfr*A17^e^, *tet*(A), *mphA*	None-detected
pEK516	-	64.5	RepFIIA (F2:A-:B-)	*bla*_CTX-M-15_, *bla*_TEM-1_, *bla*_OXA-1_, *aac*(6′)-Ib-cr, *aac*(3')-IIa, *cat*B4, *tet*(A)	None-detected
pGUE-NDM	-	87.0	RepFIIA (F2:A-:B-)	*bla*_NDM-1_, *bla*_OXA-1_, *ble*_MBL_, *sul1*, *aac*(6')-Ib-cr, *aac*(3')-II, *aad*A2, *dfr*A12, Δ*cat*B4	*traT*
pE61BA_1	D	137	RepFIIA-RepFIB (F2:A-:B1)	*bla*_TEM-1_, *tet*(A)	*ompT, iss, iroBCDEN, iucABCD, cvaBC, traT, etsABC, mig14-hlyF-finO*
pECSF1	Commensal	122	RepFIIA-RepFIB (F29:A-:B10)	None-detected	*traT, finO*
pE35BA_2+3	B	211	RepFIA-RepFIB (F-:A2:B1)	*bla*_CTX-M-15_, *bla*_TEM-1_, *bla*_OXA-1_, s*ul2, strA, strB*, *aac*(6′)-Ib-cr, *aac*(3′)-IIa, *dfr*A14, *cat*B4, *tet*(A)	*ompT, iss, iroBCDEN, iucABCD, iutA, cvaBC, sitC, traT, etsABC, mig14-hlyF-finO*
pJIE186-2	-	138	RepFIB (F-:A-:B1)	None-detected	*ompT, iss, iroBCDEN, iucABCD, iutA, cvaBC, etsABC, mig14-hlyF-finO*
pHVH177_1	D	78.6	RepFIB (F-:A-:B31)	None-detected	*traT, etsABC, mig14-hlyF-finO*

a FAB formula according to http://pubmlst.org/plasmid/classification scheme [9].

b According to the ARG-annot database (>90% amino acid identity) [http://en.mediterranee-infection.com].

c According to our in-house database (>90% amino acid identity).

d *aac(6′)-Ib-cr*-like presents the Glu72Gly additional mutation.

e In the original paper [93] *dfrA7* is reported, instead of *dfrA17*. However, inspection of its amino acid sequence indicates it is a DfrA17 protein.

The ST131 IncF plasmids belong to four different branches of the dendrogram, as shown in Fig. 4 inset. Group I includes four plasmids similar to the well-known virulence plasmids pAPEC-ColV like (also called pS88-like), which are commonly detected among avian pathogenic *E. col*i (APEC) [2], [55]. A suitable reference is the ST131 plasmid pJIE186-2, coming from a ST131 strain previously recovered in Australia in 2006 [56]. As shown in S11A Fig., group I IncF plasmids share two large homologous regions: a 70 kb region containing virulence genes *iss, iroBCDEN, iucABCD, iutA, cvaBC* and *sitC* and the cassette *ompT-hlyF-mig14*, eventually also linked to *estABCDE* [55] and a 40 kb region containing the *tra* region and other backbone genes. Group II contains 10 MDR plasmids, 8 of which are ST131 plasmids with characteristic F2:A1:B- replicons and multiple antibiotic resistancegenes. A suitable reference is the ST131 plasmid pJJ1886-5, coming from a ST43/*fimH*30 lineage from USA. As shown in S11B Fig., group II IncF plasmids share most of their genomes. It should be noted that three of these plasmids (pFV9873_5, pEK499 and pEK516) have extensive deletions within their *tra* regions, as seen in the figure. Groups III and IV are just represented by one plasmid each. None of them contains antibiotic resistance genes and they are poor in virulence genes. While group III plasmid pHVH177_1 is not similar to any reference outside the backbone genes (S11C Fig.), the group IV plasmid pECSF1 is extensively similar to various large *E.coli* plasmids, (S11D Fig.). A more comprehensive comparison of F plasmids recovered from ST131 with previously described F-like plasmids is given in Suppl. Mat.

#### Analysis of other ST131 plasmid groups

Besides MOB_F12_/IncF plasmids, 28 other plasmids were represented in ST131 isolates (Table 1). Ten were large, presumably conjugative plasmids (>18 kb), while 17 were small plasmids (<12 kb) and there was one IME.

Among the large plasmids, a most remarkable branch is composed by two almost identical 109 kb plasmids (pBIDMC20B_2 and pBWH24_2) present in two ST43/*H*30 isolates of virotype C, for which only RepFIB (Rep3-superfamily) and the maintenance protein ParB could be identified as plasmid backbone genes. No conjugative genes were detected. On the other hand, they code for an integrase protein and several phage-typical proteins. The plasmids are highly similar to pECOH89, recently recovered from a CTX-M-15 producer *E. coli* isolate from Germany [57]. Closest reference hits were the adherent invasive *E. coli* (AIEC) plasmid pLF82, isolated from a patient with Crohn's disease [58], the STEC plasmid p09EL50 [5], the *Salmonella* plasmid pHCM2 [59] and the *Salmonella* bacteriophage SSU5 [60]. These are all cryptic plasmids isolated from pathogenic enterobacteria that have been barely analyzed and thus are poorly annotated. The possibility arises of these elements being similar to lysogenic phages that are stably maintained as plasmids, analogous to phage P1 [57]. S12 Fig. compares this branch of related plasmids, using plasmid pECOH89 as a reference. As can be seen, both ST131 plasmids share most of their sequences with this 111 kb plasmid, including several phage-like protein genes. Significantly, none of the cryptic plasmids described in this study or those mentioned in the references, except pECOH89, harbor a resistance gene.

The 98.3 kb MOB_P12_/IncK plasmid pE2022_1 is most similar to pCT [61]. pE2022_1 contains a *bla*_CTX-M-14_ gene identical to that in pCT, a plasmid carrying *bla*_CTX-M-14_ that is globally spread among humans and animals and particularly prevalent in clinical isolates form Spain [61], [62]. The backbone of plasmid pE2022_1 is homologous to that of the reference IncI1 plasmid pEK204. These plasmids are described in S13. Fig. Despite their different Inc names, IncI1, IncK and IncB/O have similar backbones, belonging to different branches of the IncI complex (an analogous case to IncF plasmids).

MOB_P6_ is a large plasmid family, as can be observed in the phylogenetic tree of the MOB_P6_ relaxase family (S14A Fig.). The two ST131 MOB_P6_/IncI2 plasmids are rather different, as judged by the distant positions of their REL. Plasmid pBWH24_3 (60.3 kb) is similar to the IncI2 prototype R721 [63], but most similar to the APEC plasmid pChi7122_3 [64]. In turn, pE61BA_7 (37.9 kb) is most similar to *Salmonella agona* plasmid SL483 and the enterohemorrhagic *E. coli* (EHEC) plasmid pO157_Sal [65]. They were recovered from isolates identified as *H*30_virotype C from the USA and *H*324_virotype D from Spain, respectively.

Another ST131 important group is MOB_P3_/IncX, composed by three ST131 plasmids (pFV9873_4 and pE2022_3, as well as the reference pJIE143). Plasmids pFV9873_4 and pE2022_3, obtained from different *H*30 subgroups, are rather different between them and belong to different plasmid groups (IncX1 and IncX4), as shown their relatively distant positions in the MOB_P3_ phylogenetic tree of S15A Fig., even if showing similar sizes of about 34 kb. Their coding capacity is shown in the BRIG representations shown in S15B and S15C Fig. The IncX1 plasmid pFV9873_4 is most similar to the EC plasmid p2ESCUM [66], although genetic similarity is constrained to their backbone genes, occupying about half of the reference plasmid sequences. Conversely, the IncX4 plasmid pE2022_3 is most similar to pSH696_34 and the ST131 reference plasmid pJIE143 all over its sequence length (S15C Fig.).

Two plasmids and the IME belong to the MOB_P11_/IncP1 family, as shown in the MOB_P11_ relaxase phylogenetic tree of S16A Fig. Plasmids pJJ1886_4 and pE61BA_4 showed widely different sizes (56 and 18 kb, respectively). Plasmid pE61BA_4 is only distantly related in its backbone genes to environmental plasmid pMBUI2, isolated from an uncultured bacterium [67]. Plasmid pJJ1886_4, on the other hand, is similar to the *E. coli* plasmid pHS102707 (GeneBank Acc. N° KF701335). These two plasmids thus represent new additions to the ST131 plasmidome (see S16B Fig.). The IME_E35BA is a 14.2 kb insertion within a 234 kb chromosomal contig. S16C Fig. shows some detail on the genetic structure of the IME and its insertion site in the ST131 core genome.

The last potentially conjugative plasmid is the 24.5 kb MOB_C12_ plasmid pE61BA_2, also not closely related to any reference, as seen in the REL phylogenetic tree of S17A Fig. This plasmid resulted in a single contig, so it could be closed. The closest homolog is the *Yersinia pestis* plasmid pCRY, with which it shares all backbone genes. Its RIP belongs to the Rep_FIIA superfamily, although this plasmid group is not represented in the classical PBRT method [8]. It does not contain any gene with known adaptive function, except a protease and a putative secreted thermonuclease. As in the case above, this plasmid represents a new addition to the ST131 plasmidome (S17B Fig.), its relaxase being only 70% identical to its closest homolog, the pCRY relaxase.

Among the small plasmids, the first group is composed by four MOB_P5_/ColE1-like plasmids (11.8, 6.9, 6.6 and 5.6 kb). Three of the plasmids are relatively different, as judged by the MOB_P5_ phylogenetic tree of S18A Fig. The large plasmid (pBIDMC38_1) contains a type II restriction-modification system (Cfr10I) and is almost identical to the ST131 reference pJJ1886_3 (S18B Fig.). The two MOB_P5_/ColE1 plasmids of strain E61BA (plasmids pE61BA_5 and pE61BA_6) contain colicin ColE and ColK genes, respectively (S18C and S18D Fig.). Colicins are considered both as virulence factors as well as traits that influence bacterial fitness and survival in the presence of competitors [68].

Besides, there were four almost identical MOB_Qu_ plasmids of around 4.1 kb (S19A Fig.), which populate all *H*30 subgroups (two of virotype A, one of virotype B and one of virotype C). Nothing remarkable could be distinguished it their genetic constitution, besides a common MOB region and a pIGWZ12 -like Rep protein (S19B Fig.). Four very similar MOB_Q12_ plasmids (around 5.2 kb) are also represented in *H*30 (two of virotype A, one of virotype C) and H22 (one of virotype D). They contain RIP and REL proteins but, as in the case of the MOB_Qu_ plasmids, no phenotype could be pointed out (S20 Fig.). MOB_Qu_ and MOB_Q12_ plasmids have received little attention because they are cryptic and remain unnoticed in most typing schemes. The present ST131 plasmidome analysis suggests they can be surprisingly abundant in *E. coli*. Finally, there were five no-MOB cryptic plasmids (four of them were 1.5 kb long and the other 5.0 kb). They all contain distinguishable Rep proteins (Rep_HTH_36_superfamily), without assignment in the PBRT method. Four of them are almost identical among themselves (S21 Fig.), while the fifth (pFV9873_3) was unique and unrelated to any reference. A detailed analysis of MOBF11/IncN plasmid family is detailed in S22 Fig.

## Discussion

There are two aspects of this work that will focus the discussion. On one side, the applicability, usefulness and limitations of PLACNET will be discussed. On the other, the plasmidome of *E. coli* ST131 genomes that were reconstructed by PLACNET will be analyzed as an example of the applicability of the method. Analysis of the individual reconstructed plasmids, meant for plasmid specialists, is expanded in S1 Text.

### Bacterial genomes and plasmid reconstructions

Most bacterial genomes contain more than one physical unit of DNA. Besides the main chromosome, some bacteria contain additional chromosomes and most contain plasmids. We propose that PLACNET can be used as a new method to analyze bacterial genomes. It allows the assignment of chromosomes and plasmids as separate physical units within a genome. Visual representation of the network in Cytoscape, in which plasmids appear as constellations in a starry sky, allows user-friendly apprehension of that genome constitution. We applied PLACNET in this work to analyze the plasmidome of *E. coli* ST131 genomes, but it has been shown to work also for a series of prototypic bacterial genera with different GC content and genome architecture, such as *Salmonella*, *Klebsiella*, *Agrobacterium, Staphylococcus* or *Bacillus*. As an example, the PLACNET representation of the genome of *Staphylococcus aureus* strain 118 (ST772) (GenBank acc number AJGE00000000) is shown in S23 Fig. PLACNET scope of application also includes multi-chromosome bacteria like *Vibrio* or *Brucella*, where it correctly predicts both chromosomes present in these species. One *Vibrio cholerae* Pacini 1854 genome (Bioproject ID: PRJEB2215) is shown in S24 Fig. as an example. Once contigs belonging to each plasmid are defined, classical plasmid analysis ensues, as explained in the Results section. Contigs selected as part of a single plasmid are taken together and its overall proteome used to build a clustering dendrogram with reference plasmids present in the network. The dendrogram tree gathers plasmids according to the number of homologous proteins they share, providing an indication on prototype plasmids closely related with the query plasmid. There are two issues in PLACNET analysis that require additional work and for which additional improvement can be expected:

#### Unassigned contigs and reference sets

After plasmid reconstruction, occasionally, one or a few contigs may remain unassigned. In the set of ST131 genomes analyzed in this work, there were only two unassigned contigs (>200 bp), both in E61BA (Fig. 2). The fact that only two unassigned contigs appeared in the analysis of eight *E. coli* genomes suggests that this is not a quantitatively important problem. As could be expected, unassigned contigs are more frequent in genomes for which there are fewer references available. The lack of a suitable reference set results in poor quality clustering and an increased fraction of contigs without references. It is obvious from the preceding discussion that bacterial taxons for which not enough references exist will be more problematic for plasmid reconstruction. Thus, any such project should start by the generation of a sufficiently ample set of plasmid references. In this respect, *E. coli* constitutes probably the best choice, due to the large reference set available.

#### Repeat sequences and difficult plasmids

Usually PLACNET works well because contigs belonging to individual plasmids pair with different selected references and thus cluster in disjoint connected components in the Cytoscape representation after a single pruning step. The pruning step consists in identifying the bridging contigs (network hubs) as repeat sequences (RS). Two sets of evidence were used: (i) homology to known ISs or transposons, and (ii) existence of three or more scaffold links. Contigs fulfilling these two criteria were assumed to be in fact repeated in the connected network. Thus, the pruning operation consisted of duplicating the alluded nodes and splitting their scaffold links. In the tested set of *E. coli* genomes that were used to validate PLACNET (the set of eight ST131 genomes analyzed here, the 32 genome set analyzed by de Been et al. (2014) < submitted to Plos Genet together with this work >, plus another set of 10 other ESBL genomes obtained from clinical strains of bioprojects PRJNA186205 and PRJNA202876), there was only one case in which RS pruning operation was not sufficient to obtain disjoint components. It was the case of genome E35BA, where two coexisting MOB_F12_/IncF plasmids (pE35BA_2 and pE35BA _3) could be inferred, but repeated pruning did not result in disjoint components. The evidence for the existence of two plasmids was the finding of two sets of contigs containing REL and other plasmid backbone genes. PLACNET failed in discriminating both plasmids probably because network links to references were interlocking, since several PLACNET-selected references established best hits to different components of each set. Besides, the assembly program could not distinguish among parts of both sequences and considered them as RSs. Closely related plasmids that coexist in a given cell poses the most serious problem we encountered in the application of PLACNET.

### The ST131 plasmidome

HGT plays a critical role in shaping bacterial lineages, especially those of multi-environment opportunistic pathogens. Comprehensive characterization of plasmidomes has been impeded by methodological limitations, although they are essential for multilevel population genetics analysis, an approach necessary to explain selection and diversification of bacterial populations and to understand the reservoir dynamics of antibiotic resistance and virulence genes [69]. The application of PLACNET to ST131 genomes allowed the detection of emerging plasmid variants, important for the evolutionary history of this ExPEC lineage, which constitutes an outstanding example of a “high risk clonal complex”, a concept increasingly important in Public Health [69].

#### Plasmidome description

We describe a remarkable heterogeneity of plasmids among the *E. coli* ST131 genomes analyzed, with the identification of 39 plasmids to add to the 11 plasmids in the ST131 lineage already sequenced (Table 1). Interestingly, these plasmids encompass 8 out of 17 main MOB plasmid groups found in the whole class of γ-proteobacteria [11], [53], namely F12 (IncF), P3 (IncX), P5 (ColE1), P6 (IncI2), P11 (IncP), P12 (IncI/K/BO), Q12 (Rep_pSC101-like), Qu (Rep_pMG828-2/IGWZ12-like), several of them undetectable by PBRT. Besides conjugative or mobilizable plasmids, there were other plasmids, lacking REL, but identifiable by their RIP proteins. An in-depth analysis of each plasmid group identified in this study has been diverted to a Supplementary Discussion (though exciting for clinical epidemiology or plasmid biology, it is outside the mainstream goal of this work). Our findings substantially enlarge the repertoire of plasmids identified among *E. coli* ST131 isolates, which now reflect a genome widely open for plasmid infection. It is of note that this scenario has also been described for *E. coli* clones of different pathovars [47], [70]–[73]. Comparative genomics of the few *E. coli* lineages comprehensively analyzed to date suggests that this species is a generalist able to colonize and infect humans. It also suggests that phages and plasmids make an important contribution to specialization by accessorizing the genome with new adaptive traits and tools that modify genome structure and, eventually, by modifying transcriptional regulation [70], [71], [74], [75]. It should also be emphasized that almost all available studies on ST131, included this one, focused on strains involved in the spread of antibiotic resistance genes, which constitute, undoubtedly, a biased fraction of the ST131 plasmidome and thus preclude an accurate view of its evolutionary history [76] (see also below).

#### Plasmids and *E. coli* diversification

Specific ExPEC lineages have scarcely been analyzed in the context of multilevel population genetics with the exception of punctual cases involving clonally unrelated isolates [70]. A recent phylogenomic analysis of 95 ST131 isolates from different geographical areas identified the same three clusters studied in the present work [16]. This analysis concluded that point-mutations and recombination events associated with diverse MGEs, including prophages and genomic islands, determined the diversification of this ExPEC lineage. However, the diversity of plasmids was only inferred by searching for incompatibility regions based on PBRT schemes. The role of plasmids in genomic versatility were not further analyzed [16]. Even though our study analyzed a smaller number of isolates, some observations can be drawn about the role of plasmids in the diversification of the ST131 lineage.

The rate of mutagenesis of *E. coli* has been roughly estimated in one mutation per genome per year [77]. Although this number is no doubt controversial, such study and those addressing the role of recombination in the ST131 lineage add context to understand its evolution as represented in Fig. 1. Compared to the limited sequence divergence among the ST131 core genomes (the ST43/*H*30 branch includes just about 600 SNPs), plasmids represent a very active fraction of ST131 adaptive evolution, as can be concluded from the analysis of Table 1. Such plasmid variability suggests that independent plasmid acquisitions and losses frequently occur within and between ST131 sublineages. Within the *H*30 cluster, the presence of antibiotic resistance plasmids is remarkable, specially the identification of structurally similar F2:A1:B- plasmids carrying genes conferring antibiotic resistance, since early acquisition of *bla*_CTX-M-15_, mainly associated with F2 plasmids, is considered a key event in the selection of the ST131 cluster C/*H*30 subclone [13], [16], [17]. The modular structure of F2 plasmids, containing multiple copies of ISs, facilitates gene rearrangements and the interchange of antibiotic resistance platforms linked to resistance to first line antibiotics between plasmids of the same and different families. This notion, inferred from our present analysis, has already been proposed [17], [78]–[80] and is of great concern nowadays because of the increasing risk of encountering *E. coli* isolates carrying *bla*_KPC_ of *bla*_NDM_ genes predominant in *Klebsiella* (http://www.cdc.gov/drugresistance/threat-report-2013/) [81]. Beyond F2 plasmids, the presence of other plasmid groups (N, I1/K/BO, I2, A/C, X) carrying antibiotic resistance genes is observed at variable rates in this and other studies, clearly influenced by local ecology. Most of these antibiotic resistant non-F plasmids occur only rarely in *E. coli* isolates [82]. This could be due to an intrinsic lack of fitness of these plasmids in *E. coli* under natural conditions. Alternatively, they could represent cryptic indigenous plasmids now identifiable because of the acquisition of antibiotic-resistance cassettes. Nevertheless, the acquisition of mosaic regions carrying multiple antibiotic resistance genes by broad host plasmids (e.g. N, I2, A/C) increases the risk to spread resistance to first line antibiotics to different bacterial species in and outside hospitals [79], [83]. Besides antibiotic resistance plasmids, an outstanding finding of this work was the frequent detection other plasmid groups, generally considered cryptic, that are clearly underrepresented in previous ST131 studies, as ColE1, MOB_Q_, and phage-like Rep3 plasmids. All of them are highly heterogeneous plasmid groups able to acquire adaptive traits or contribute to the mobilization of other plasmids (*See supplementary dataset for details*).

Members of the ST131 plasmidome such as MOB_F12_/IncF, MOB_H_/IncA/C and Rep3/phage-like plasmids can also shape the *E. coli* chromosome by facilitating mobilization in trans of genetic islands or integrating new genetic material [32], [70], [84]. Interestingly, recombination of large chromosomal regions occurring at the sites of insertion of either prophages or transferable genomic islands seems to have contributed to the split of the ST131 lineage in different clusters [16]. Although experimental studies on ST131 did not yet associate plasmids with genome structure, the hypothesis is plausible taking into account the frequency at which such events occur in other B2 *E. coli* populations.

On a more general note, our study identifies antibiotic resistance plasmids, which are favored in high density environments, such as the human gastrointestinal tract, and under antibiotic selective pressure, that are predominant in hospitals [85] together with phages (or phage-like cryptic plasmids), apparently predominant in low density environments [86], [87], and other cryptic plasmids (frequently very small and devoid of any possible adaptive gene). This mixed constitution, which is difficult to understand on purely selective grounds, highlights potential roles of plasmids in the context of multilevel selection, a recurrent issue in evolutionary biology. The plasmid flux in ST131 strains occurs while disseminating genes coding for resistance to extended spectrum beta-lactamases (ESBL). The results presented here find a complement in the study of de Been *et al*. (accompanying paper), where the authors document the dissemination of ESBL-carrying epidemic plasmids from animal to human clonally unrelated *E. coli* lineages. Thus, many plasmids appear in a clonal lineage, and many lineages can be infected by a single predominant plasmid. These conceptual notions have relevance in Public Health as they deal with the hierarchical units of selection that contribute to increase the population size of antibiotic resistance genes in human and animal pathogens [69], [88], [89].

In summary, our study reveals the utility of PLACNET in multilevel population genetics analysis, critical to understand the evolutionary processes and dynamics of both bacterial and plasmid lineages. Its application to *E. coli* ST131 allowed us to infer the roles of plasmids in the dissemination of globally spread antibiotic resistance and virulencegenes, some of them being underrepresented in Genbank. It is probable that these plasmids are critically relevant to understand the adaptive evolution of *E. coli* populations and their bacterial exchange communities. Armed with this new tool for plasmid analysis, future scrutiny of a larger number of significant strains will allow us to understand the interplay among different plasmid associations that often appear in bacterial pathogens.

### Conclusion

The evolutionary processes of main bacterial pathogens are often discussed in the context of lineage-associated acquisition of a specific virulence gene set. The present study demonstrates how *E. coli* ST131 strains, even when they are practically identical in their core genomes, contain a striking variety of different plasmids. Many of them remain unnoticed, since they are apparently cryptic. Prevalent plasmids, such as IncFs, undergo frequent recombination, continuously resulting in novel gene repertoires. Our results shed light on the role of plasmids in *E. coli* ST131 evolution. Horizontal transmission of plasmids that carry not only antibiotic resistance and virulence genes, but also other poorly analyzed functions (metabolic genes, colicins and as yet cryptic functions) is common in the ST131 plasmidome and results in frequent and rapid adaptive changes. Arrival to these conclusions has been made possible by the application of PLACNET, a plasmid reconstruction method for WGS datasets.

## Materials and Methods

### Epidemiological background of bacteria and plasmids

Comprehensive plasmidome analysis was carried out for 10 *E. coli* ST131 genomes, representing main ST131 sublineages described to date [13], [16]. They include strains coming from Spain (three *fimH*30, one *fimH*324), USA (three *fimH*30), Australia (one *fimH*30), Denmark (one *fimH*22) and Japan (one *fimH*41). The *fimH*30 strains from Spain were CTX-M-15 producers and belonged to the *H*30-Rx sublineage (additionally, one strain was also CTX-M-14), while those collected in the USA were KPC-2 producers. The four strains from Spain represent predominant ST131 variants on the basis of PFGE patterns and the presence/absence of four putative virulence markers (*afaFM955459*, encoding an Afa/Dr adhesion; *sat*, secreted autotransporter toxin; *ibeA*, invasion of brain endothelium; and *iroN*, salmochelin siderophore receptor) [36], [51], [52] and sequenced for this work.

The ST131 isolates studied represent epidemic variants exhibiting particular combination of putative virulence traits and were previously designed as distinct “virotypes” by capital letters A to D [36]. It should be noted that no correlation exists between these “virotype” designations and “ST131 strain designation” in other studies that also used capital letters to distinguish among ST131 clonal variants [16]. Other genome datasets were taken either from Bioproject NCBI database (https://www.ncbi.nlm.nih.gov/bioproject/), or from NCBI genomes database (*E. coli* JJ1886 [90] and *E. coli* SE15 [91]). In addition, fully sequenced plasmids pEK499, pEK204, pEK516, pJIE186-2 and pJIE143, previously found in other ST131 isolates [56], [92]–[94], were used for plasmid comparisons. Information about all genomes is detailed in Table 3.

**Table 3:** Human *E. coli* ST131 genomes analyzed in this work.

Strain	Accession^a^	Location	Collection date	Isolation source	Plasmid name (Accession number)^b^	Reference
HVH-177	PRJNA186205	Denmark	2003	Blood	pHVH177_1	PRJNA186413
BIDMC20B	PRJNA202031	USA	-	Urine	pBIDMC20B_1and _2	PRJNA202876
BWH24	PRJNA201983	USA	-	-	pBWH24_1 to _3	PRJNA202876
BIDMC38	PRJNA202050	USA	2012	-	pBIDMC38_1 to _5	PRJNA202876
FV9873	PRJEB6262	Spain	2007	Urine	pFV9873_1 to _6	This study
E35BA	PRJEB6262	Spain	2008	Urine	pE35BA_1 to _3, IME_E35BA	This study
E2022	PRJEB6262	Spain	2006	Urine	pE2022_1 to _5	This study
E61BA	PRJEB6262	Spain	2008	Abscess	pE61BA_1 to _7	This study
SE15	AP009378	Japan	-	Feces	pECSF1 (AP009379)	[91]
JJ1886	NC_022648.1	USA	-	Urine	pJJ1886_1 to _5 (NC_022649; NC_022650; NC_022651; NC_022661; NC_022662)	[90]

a PRJNA and PRJEB6262 accession numbers correspond to SRA datasets. AP009378 and NC_022648.1 correspond to finished genomes.

b Plasmids derived from this study are named according to Table 1.

### DNA sequencing

Total DNA from *E. coli* ST131 strains FV9873, E35BA, E2022 and E61BA was extracted with QIAmp DNA Mini Kit (Qiagen). DNA concentration was measured with Nanodrop 2000 (Thermo Scientific) and Qubit 2.0 Fluorometer (Life Technologies). 1.0 µg DNA was sonicated (20 cycles of 30 s at 4°C, low intensity) with Bioruptor Next Generation (Diagenode). Sample quality was checked in a Bioanalyzer 2100 (Agilent Technologies). DNA samples were preconditioned for sequencing by using the TruSeq DNA Sample Preparation Kit (Illumina) and quantified with Step One Plus Real-Time PCR System (Applied Biosystems). Flow-cells were prepared with TruSeq PE Cluster Kit v5-CS-GA (Illumina). Sequencing was carried out using a standard 2×71 base protocol (300-400 bp insert size) in a Genome Analyzer IIx (Illumina, San Diego, CA) at the sequencing facility of the University of Cantabria. The main statistics of the eight sequence datasets analyzed are shown in Table 4.

**Table 4:** Genomes assembled in this study.

Strain	ID	N° Libraries	Read length	N°Contigs	Total bp	N50	Kmer
HVH177	SRS399685	2	101	81	5035548	242711	83
BIDMC20B	SRS420795	3	101	115	5311918	209342	91
BWH24	SRS420803	2	101	114	5369063	192138	83
BIDMC38	SRS420798	4	101	135	5226831	190988	91
FV9873	ERS450218	3	71	262	5160060	153685	55
E35BA	ERS450219	3	71	419	5243070	159702	57
E2022	ERS450220	2	71	346	5296607	159635	53
E61BA	ERS450221	3	71	246	5168482	198396	57

### Phylogenetic analysis of the ST131 core genome

The ST131 core genome was defined as the collection of genes present in the ten ST131 genomes analyzed, with more than 90% similarity and 90% coverage. CD-HIT-EST [95] was used to cluster genes. A homemade Perl script was created to parse the cluster and define the core genome set. All core genes were concatenated and aligned with progressive Mauve [96]. A tabular list of SNPs was extracted from the Mauve alignment by applying the SNP export tool of Mauve GUI. The tabular list of polymorphic sites was parsed by a homemade script. A given position was counted as an SNP if it varied between two given sequences. The number of SNPs was added for each pair of strains to give the final SNP count. Polymorphic sites with gaps were removed from the SNP count matrix. The Mauve alignment was curated by trimAl [97]. RAxML [98] was used to build the core genome phylogenetic tree, using 100 replicates for bootstrap determination.

### Plasmid Constellation Networks (PLACNET)

**Figure 6:**
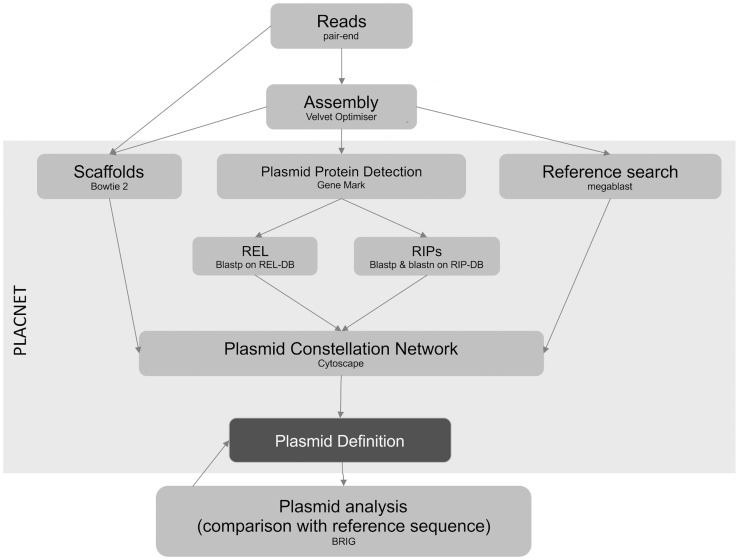
PLACNET flow diagram. The diagram represents the PLACNET workflow to analyze an Illumina bacterial genome dataset. It can be separated in two sub-process: network delineation and plasmid analysis. Network delineation consists on contig assembly, determination of scaffold interactions, reference search of homologous genomes and plasmid protein prediction. Plasmid analysis basically consists in the construction of a dendrogram of plasmid protein profiles, which identifies the most relevant reference sequences, followed by plasmid cluster analysis, which compares query plasmids with its closest references. Plasmid analysis is a feedback process that helps to resolve uncertainties and results in a final definition of plasmid and chromosome content.

PLACNET was developed to associate contigs with specific physical DNA units in WGS experiments. Networks are powerful models that allow visualization and analysis of sequence information. PLACNET networks are composed of two types of nodes (contigs and reference genomes) and two types of edges (similarity to reference sequences and scaffold links). Commonly, network layout algorithms simulate repulsion forces between nodes and attraction forces by the edges that link two nodes. Thus, node distribution in the network will depend on the intensity of forces that define the edges. In such network model, a plasmid will be represented by a connected component (a set of linked nodes) or, in other words, a constellation of contigs. Different physical units (plasmids and chromosomes) should be represented by disjoint connected components (separate constellations). The workflow (Fig. 6) involves the following steps:

#### Assembly

Velvet assembly software [99] and its script VelvetOptimiser.pl were used to determine the best assembly and to scan the optimum parameters. Velvet provides also coverage information for each contig, which adds useful information for network interpretation.

#### Scaffold links

Although assembly programs perform scaffolding between contigs, when the assignation is ambiguous, contigs remain unbound. A method based in the mapping tool Bowtie 2 [100] was used to find all possible scaffold links. All reads were mapped using the contigs as references, using default parameters of Bowtie 2, with the option to report all possible hits. The output file was converted to SAM format [101] to give the potential adjacency information for each contig. We considered as potential PLACNET scaffold links those which comply with two rules: (i) the contigs were paired at their extremities, themselves defined as twice the read length, and (ii) the number of pair-end reads linking those two contigs had to be higher than one third of the mean of the total pair-end reads that scaffold all contigs. This procedure was implemented as an in-house Perl script.

#### Reference search

At least for bacteria widely covered by sequencing projects, most contigs in any new sequence are similar to one or more previously published sequences (reference sequences). Our initial hypothesis was that, for any physical DNA unit, its contigs will “BLAST” a related set of reference sequences. Thus, in the PLACNET network representation, they will cluster around the homologous references. The more DNA databases grow, the closer the references will be to the query sequence and the better PLACNET will work. A homemade BLAST [102] database was constructed from the NCBI genomes database by joining all sequences contained in [ftp://ftp.ncbi.nlm.nih.gov/genomes/Bacteria/] and [ftp://ftp.ncbi.nlm.nih.gov/genomes/Plasmids/]. The version used in this work was from March 7^th^ 2013 and contains 6,432 genomes (plasmids and chromosomes). Megablast search of all contigs was carried out against the homemade BLAST-genome database with the objective of selecting a few best matches for network construction. Due to the different length of each contig, fixed thresholds by e-value or score cannot be chosen. Since the score is not a normalized parameter, and varies depending on sequence length, hits were selected by applying a dynamic threshold, based on the number of homologous sequences and the score of each hit. If the threshold is defined as 85%+2*n* of the mean of the *n* previous sequence scores, and *n* is the ranking position of sequence *i* retrieved by megablast with score *S_i_*, then *T_n+1_* is the threshold for sequence *n+1*:
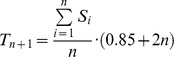



All reference sequences above the threshold were taken as nodes in the PLACNET representation.

#### Protein prediction of replication initiator proteins (RIP) and relaxases (REL)

Some genes are indicative of a plasmid sequence. Among them we selected REL, key proteins in the conjugation process [53], [103] and RIPs, key proteins in the replication of most plasmids [104]. Although not all plasmids contain a RIP and/or REL, their presence in a contig is diagnostic of a plasmid (or ICE) sequence. Some plasmids have more than one RIP (i.e. IncF family plasmids) [9], [105], [106] but plasmids rarely have more than one REL [107]. ORF prediction was carried out by GeneMark [108], which optimizes predictions based on GC content of DNA. The heuristic prediction implemented in this software is especially useful to predict ORFs in plasmid-containing genomes because it takes each contig individually and selects the best prediction model case by case.

To implement specific search protocols for REL and RIPs, three homemade databases (DB) were developed. A REL database (REL-DB) was constructed according to [53], [109]. Similarly, RIP-DB was constructed from all RIPs annotated in UniProt database. RIP sequences were clustered by CD-HIT [95], using 40% identity as a threshold. Next, a Hidden Markov Model was built from each cluster by hmmer3 [110]. Finally, the HMM profiles were used in a search against UniProt. The HMM search and initial dataset were joined in one database (RIP-DB). An additional step was necessary to classify RIPs according to the widely used plasmid classification protocol PBRT [8]. A homemade nucleotide database (INC-DB) was created to identify PBRT types in silico using blastn. Finally, the relevant ORFs (REL and RIPs) identified to specific contigs by using REL-DB and RIP-DB were incorporated to the network as tags. All relevant steps in network construction were implemented as a Perl script available at the following web page: http://placnet.sourceforge.net/.

#### Plasmid constellations

As explained above, each plasmid is represented in PLACNET by a connected component (a constellation). Thus, different physical units (plasmids and chromosomes) should be represented by disjoint (unlinked) connected components. Cytoscape software [111] was used to visualize and analyze plasmid constellations, which incorporate all the information (similarity to reference sequences, scaffold links, and protein tags) in a single network. Node attributes such as contig size, coverage and reference description, are added to the network. At this stage, network pruning is needed to resolve individual plasmids as disjoint components. When a genome has a number of repeated sequences (e.g., insertion sequences (ISs) or transposons), or two very similar plasmids, the assembly process outputs those sequences as contigs with multiple scaffold links. In the network context, they represent hubs, that is, nodes with a high number of connections. This makes the network very dense and complicates the analysis of network connected components. In PLACNET, contigs smaller than 200 bp were directly eliminated from the analysis. Hubs were examined by *blastx* against protein databases (i.e. UniProtKN or NCBI nr). If there was identity to any transposase gene, the hub was duplicated, and scaffold links were partitioned among them, to maximize the number of disjoint components. Contigs that remain unbound are classified as “unassigned sequence” in the contig assignation table.

#### Plasmid definition, dendrograms and cluster analysis

The final steps in plasmid reconstruction involve the definition and verification of each plasmid. This is an iterative process, as shown in Fig. 6. First, each contig was assigned to a putative plasmid (or chromosome) based on visualization of disjoint connected components in the Cytoscape representation. Assignments take into consideration additional types of evidence like the presence of REL and/or RIPs, size of the putative plasmid compared to reference plasmids and coverage of each contig (contigs belonging to the same plasmid must have similar coverage). Taking into account the information provided by related genomes within the same sequencing project can also be helpful (same sequences cluster around the same references). In this respect, PLACNET is more robust in multi-strain collections. The performance of PLACNET was validated by testing a number of previously sequenced and annotated *E. coli* genomes (S2 Table). The ART software (Huang et al., 2012) was used to simulate pair-end Illumina reads from those genomes, which were then analyzed by PLACNET as explained above. Results are shown in S2 Text, S3 Table and S25-S34 Figs.

After PLACNET has defined the plasmids carried in the relevant genomes, the next step in plasmidome analysis is to build a dendrogram that produces a hierarchical clustering of plasmid proteomes similar to those described in [112]–[114]. CD-HIT (thresholds: 70% identity and 80% coverage) was used for clustering references and query plasmids. Based on the output file, a presence/absence table (present or absence of each protein cluster in each plasmid) was built. Each table row represents a plasmid protein profile. Raup-Crick distance method, implemented in *vegan* package for R software [115], was used to calculate the distance matrix of plasmid protein profiles. The Ape package [116] was used to calculate the dendrogram bootstrapping confidence value. Finally, a hierarchical clustering dendrogram was built using the UPGMA algorithm.

Putative plasmids and references belonging to the same dendrogram branch were compared using BRIG [117] or Abacas [118]. While BRIG is not sensitive to contig arrangement, Abacas can be used to order contigs according to a given reference. With these tools, the curator is able to visualize the correspondence between reconstructed plasmids and references, or can go back to dendrograms or PLACNET in the search for missing or extra contigs. This iterative mode of analysis is represented in Fig. 6 by the backward arrow linking plasmid cluster analysis with plasmid definition.

Plasmids were mainly classified according to their REL in MOB families, as described by [53]. Classical Inc families are also given when typing them by in silico PBRT was possible. Plasmids that could not be classified one way or the other were termed no-MOB by exclusion.

## Supporting Information

S1 Fig PLACNET reconstruction for FV9873 genome (*E. coli* ST131/*H*30/virotype A). A total of 262 contigs were classified in chromosome and six plasmids. The black line surrounding the pFV9873_1 plasmid (4.1 bp) indicates that it is a closed plasmid. There are not unassigned contigs.
(PDF)

S2 Fig PLACNET reconstruction for E35BA genome (*E.coli* ST131/*H*30/virotype B). One 14.2 kb MOBP11 Integrative Mobilizable Element (IME) was detected in the chromosome. The pE35BA_1 plasmid (4.1 kb) is closed. There was a conflict of separation between two IncF plasmids (pE35BA_2 and pE35BA_3, total size: 211 kb). The annotation of this particular genome is limited by the quality of the assembly (many small and non-scaffolded contigs). This is the network with the highest number of contigs in our study (total: 419). No contigs remained unassigned.
(PDF)

S3 Fig PLACNET reconstruction for E2022 genome (*E. coli* ST131/*H*30/virotype C). A total of five plasmids, two of them as closed plasmids, and the chromosome were obtained in a 346-contig network. No contigs remained unassigned.
(PDF)

S4 Fig PLACNET reconstruction for E61BA genome (*E. coli* ST131/*H*324/virotype D). Seven different plasmids were obtained in the analysis. The pE61BA_2 plasmid (24.5 kb), containing a single contig, was closed. Two contigs remained unassigned, as no scaffold links were detected for them. One of them (2,953 bp) encodes for a putative DNA primase and a lytic transglycosilase, while another (1,301 bp) encodes for TrbI and TraB partial proteins.
(PDF)

S5 Fig PLACNET reconstruction for BIDMC20B genome (*E. coli* ST131/*H*30/virotype C). A total of 115 contigs were fully assigned to the chromosome and two plasmids. Plasmid pBIDMC20B_2 (109 kb) appeared as a single contig that could be closed.
(PDF)

S6 Fig PLACNET reconstruction for BIDMC38 genome (*E. coli* ST131/*H*30/virotype A). Five plasmids were detected. Three small plasmids (1.6, 4.2 and 5.3 kb) are closed. No contigs remained unassigned.
(PDF)

S7 Fig PLACNET reconstruction for BWH24 genome (*E. coli* ST131/*H*30/virotype C). Three plasmids were detected, only one of them as a closed plasmid (pBWH24_2, 109 kb). No contigs remained unassigned.
(PDF)

S8 Fig PLACNET reconstruction for HVH177 genome (*E. coli* ST131/*H*324/virotype D). Only one plasmid (pHVH177_1, 78.6 kb) composed by three contigs was detected in the HVH177 genome. No contigs remained unassigned.
(PDF)

S9 Fig Contig coverage in E61BA genome (*E. coli* ST131/*H*324/virotype D). Contigs belonging to each plasmid are shown in different colors, according to the code below the histogram. Plasmid copy numbers are inferred from their contig coverage. Average coverage of chromosomal contigs was 56. All contigs with >2X average are named according to their predicted gene products. When there is more than one contig, annotations are separated by colons, if adequate.
(PDF)

S10 Fig Contig coverage in E35BA genome (*E.coli* ST131/*H*30/virotype B). Contigs belonging to each plasmid are shown in different colors, according to the code below the histogram. Plasmid copy numbers are inferred from their contig coverage. Average coverage of chromosomal contigs was 55. All contigs with >2X average were named according to their predicted gene products. If more than one contig coincided within the same coverage section, annotations of the individual contigs were separated by colons. As is shown in the histogram, contigs corresponging to the MOBF12/IncF plasmids pE35BA_2+3 (in green color) show similar coverage than the chromosome, which indicates that both IncF plasmids have the same copy number than the chromosome.
(PDF)

S11 Fig BRIG comparative analysis of MOBF12/IncF plasmids. These plasmids were subdivided in four groups according to Fig. 5 inset. **S11A**: Group I. Plasmid pJIE186-2 is used as the reference for the BRIG comparison. **S11B**: Group II. Plasmid pJJ1886-5 is the inner reference. **S11C**: Plasmid F is used as a reference. **S11D**: Plasmid pECSF1 is used as a reference.
(PDF)

S12 Fig Comparative analysis of phage-related/RepFIB plasmids. **S12A**: BRIG representation using the 111kb plasmid pECOH89 as reference. **S12B**: Phylogenetic analysis of RepFIB family of RIP proteins. RaxML software (v.7.2.8) was used to infer the Maximum Likelihood tree and MEGA5.2.2 to represent the result. Bootstrap values for 100 replicates are indicated. The tree was rooted with the RepFIB protein of the IncN plasmid N3.
(PDF)

S13 Fig BRIG comparative analysis of MOBP12/IncI-complex. **S13A**: The IncI1 plasmid pEK204 is used as inner ring in the BRIG analysis. **S13B**: Plasmid pCT [62] was used as reference.
(PDF)

S14 Fig Comparative analysis of MOBP6/IncI2 plasmids. **S14A**: Phylogenetic tree of MOBP6 REL proteins, calculated as in S12B Fig. The tree was rooted with MOBP6 REL of Plasmid2 from *Nitrosomonas eutropha* C91. **S14B**: BRIG comparative analysis of pBWH24_3 plasmid, using pChi7122_3 as reference. **S14C**: BRIG comparative analysis of pE61BA_7 plasmid, using pO157_Sal as inner reference.
(PDF)

S15 Fig Comparative analysis of MOBP3/IncX plasmids. **S15A**: Phylogenetic tree of MOBP3 REL proteins, calculated as in S12B Fig. The tree was rooted with VirD2_pSD25 (MOBP2 subfamily). ST131 plasmids are shown in red. IncX subgroups are indicated in different color backgrounds. **S15B**: BRIG comparative analysis of IncX1 plasmids using p2ESCUM as a reference. **S15C**: BRIG comparative analysis of IncX4-like plasmids using pSH696_34 as reference.
(PDF)

S16 Fig Comparative analysis of MOBP11/IncP plasmids. **S16A**: Phylogenetic tree of MOBP11 REL proteins, calculated as in S12B Fig. The tree was rooted with NikB_R64 (MOBP12 subfamily). ST131 plasmids are colored in red. Two clearly separated groups are colored. **S16B**: BRIG comparative analysis of IncP1 plasmids using pJJ1886_4 as a reference. **S16C**: Comparison of JJ1886 and E35BA genomes, showing the genetic map of the inserted IME_E35BA, and its homology to *Bukholderia glumae* IncP island. The figure was drawn with EasyFig [75]. Specific genes are specifically colored according to the code in the lower part of the figure.
(PDF)

S17 Fig Comparative analysis of MOBC12 plasmid. **S17A**: Phylogenetic tree of MOBC12 REL proteins, calculated as in S12B Fig. The tree was rooted with MobC_CloDF13 (MOBC11 subfamily). **S17B**: BRIG comparative analysis of MOBC12 plasmids using pCRY as a reference.
(PDF)

S18 Fig Comparative analysis of MOBP5/ColE1-like plasmids. **S18A**: Phylogenetic tree of MOBP5 REL proteins, calculated as in Fig SF12B. **S18B, C and D**: BRIG comparative analysis of MOBP5 plasmids using ColE1 (SF18B), pJJ1886_3 (SF18C) and pColK-K235 (SF18D) as references.
(PDF)

S19 Fig Comparative analysis of MOBQu plasmids. **S19A**: Phylogenetic tree of MOBQu REL proteins, calculated as in Fig SF12B. The tree was rooted with the MOBQu2 subfamily. ST131 plasmids are colored in red. Different color backgrounds are used to represent MOBQu1, where ST131 MOBQu plasmids are located, and MOBQu2 branches. **S19B**: BRIG comparative analysis of MOBQu plasmids using pSE11-6 as a reference.
(PDF)

S20 Fig Comparative analysis of MOBQ12 plasmids. **S20A**: Phylogenetic tree of MOBQ12 REL proteins, calculated as in Fig SF12B. The tree was rooted with MobA_RSF1010 (MOBQ11 subfamily). **S20B**: BRIG comparative analysis of MOBQ12 plasmids using pCE10B as a reference.
(PDF)

S21 Fig Comparative analysis of small no-MOB plasmids. BRIG comparative analysis of no-MOB plasmids using pCE10D as a reference.
(PDF)

S22 Fig Comparative analysis of MOBF11/IncN plasmids. **S22A**: Phylogenetic tree of MOBF11 REL proteins, calculated as in Fig SF12B. The tree was rooted with R388 (MOBF11/IncW). IncN1 and IncN2 subgroups are indicated with different background colors. **S22B**: BRIG comparative analysis of IncN1 plasmids, using R46 as a reference. **S22C**: BRIG comparative analysis of IncN2 plasmids using p271A as a reference.
(PDF)

S23 Fig PLACNET reconstruction of the genome of *Staphylococcus aureus* strain 118 (ST772) (ID: PRJNA82607). Assembly data: Number of libraries: 1; read length: 75 bp; number of contigs: 73; total bp: 2,798,022 bp; N50: 224673 and Kmer: 73. One 12,819 bp plasmid was identified and reconstructed. No REL or RIP proteins were detected.
(PDF)

S24 Fig PLACNET reconstruction for a genome of *Vibrio cholerae* Pacini 1854 (ID: PRJEB2215). Assembly data: Number of libraries: 1; read length: 75 bp; number of contigs: 149; total bp: 4,022,287 bp; N50: 180089 and Kmer: 65. The two *V. cholerae* chromosomes were fully reconstructed. Chromosome I (2,992,142 bp in our study) was reported to be about 3 million bp and encodes most essential functions [95]. As shown in Fig S24, it harbors a MOBH12 relaxase, identical to that of the integrative and conjugative element (ICE) of the SXT/R391 family [96]. Chromosome II is smaller (1,034,286 bp in our study). Finally, a 26.3 kb plasmid containing a RIP protein (87% identity with *E.coli* pABU plasmid [97]) could be reconstructed.
(PDF)

S25 Fig Cytoscape representation of the reconstructed *E. coli* JJ1886 genome. The network was constructed and codes used (in this and following figures) as explained in Fig. 6. The pruned network (Step 1) was obtained after deleting 19 contigs smaller than 200 bp.
(PDF)

S26 Fig Definition (Step 2) of *E. coli* JJ1886 plasmids p1 to p4. These plasmids contain a RIP and/or REL protein and appeared as single contigs. The inset Table shows some properties of relevant nodes. The nodes are represented in the network surrounded by circles of the same color than the background color in the Table.
(PDF)

S27 Fig Resolution of hubs and definition of plasmid p5 (Step 3). Hub nodes that were duplicated are shown in the inset Table and indicated by red arrows in the Cytoscape network. After hub duplication, the IncF plasmid p5 is shown to contain the 12 contigs surrounded by a red circle.
(PDF)

S28 Fig BRIG comparison of the reconstructed plasmid p5 with the reference plasmid pJJ1886_5. The reference plasmid (inner ring) is compared to the p5 reconstructed plasmid (purple ring). Outer black and white ring sectors represent pJJ1886_5 gene annotations.
(PDF)

S29 Fig Inverse BRIG comparison of reconstructed plasmid p5 vs pJJ1886_5 reference plasmid. The reconstructed plasmid is placed here as the reference inner ring (thin black circle line) to which the reference plasmid (purple ring) and the reconstructed p5 contigs (outer blue and red ring) are compared.
(PDF)

S30 Fig Cytoscape representation of the reconstructed *E. coli* SE15 genome. The network was constructed and codes used as explained in Fig. 6. The pruned network (Step 1) was obtained after deleting 16 contigs smaller than 200 bp.
(PDF)

S31 Fig Plasmid definition (PLACNET steps 2 and 3) of *E. coli* SE15 genome. Three particular nodes in the pruned network (surrounded by the red circle) were scrutinized due to their loose connection to the chromosome. As shown in the inset Table (red background files), a blastx comparison indicates they correspond to “typical” *E. coli* chromosomal segments, so were finally assigned to the chromosome. Three other nodes (surrounded by a green circle in the left Cytoscape representation) corresponded to hubs (green background in the inset) and were thus duplicated. The reconstructed plasmid (p1) in the final network is surrounded by a purple ring.
(PDF)

S32 Fig BRIG comparison of SE15 *E. coli* genome reconstructed IncF plasmid (p1) with the reference plasmid pECSF1. The reference plasmid (inner ring) is compared to the IncF reconstructed plasmid (purple ring). Outer black and white ring sectors represent pECSF1 gene annotations. Three regions (5,709 bp in total) were missing from the p1 reconstruction.
(PDF)

S33 Fig Cytoscape representation of the reconstructed genome of *E. coli* strain MG1655 containing plasmids pEC958 and R46. The network was constructed and codes used as explained in Fig. 6. The pruned network was obtained after deleting 25 contigs smaller than 200 bp and duplicating 2 hubs (surrounded by a red circle). Plasmid p1 is the reconstructed R46 while p2 is the reconstructed pEC958. Nodes surrounded by a blued circle, and described by the blue background files in the inset Table, could not be assigned. See text for further details.
(PDF)

S34 Fig Final Cytoscape representation of reconstructed H0407 *E. coli* genome. The network was constructed as explained in Fig. 6. The pruned network was obtained after deleting 44 contigs smaller than 200 bp. Plasmids p1 and p2, represented by single contigs, are surrounded by red and blue circles, respectively. A single contig (surrounded in an intense blue circle) remained isolated from other genetic units. Nevertheless, blastx analysis demonstrate it correspond to chromosomal background, as shown in the inset Table. Nodes described by the grey background files in the inset Table correspond to one node assigned to the chromosome, plus seven hubs, which were duplicated. The green circle surrounds 52 contigs, adding 119,735 bp, which represent plasmids p3 and p4, the two IncF plasmids that PLACNET was not able to resolve (see text for further details). The red arrow indicates a node containing REL, RIP and backbone genes in common to both IncF plasmids.
(PDF)

S35 Fig Hierarchical clustering dendrogram of ST131 plasmids and relevant references. The left dendrogram shows the complete tree, with references. Dendrogram construction and color codes are as in Fig. 4. The right dendrogram expands the MOB_F12_/IncF branch, with new background colors highlighting plasmid groups within this branch that are mentioned in the text.
(TIF)

S1 Table Complete list of contigs assigned to each plasmid or chromosome.
(XLS)

S2 Table Ten *E. coli* genomes analyzed as examples of PLACNET performance.
(PDF)

S3 Table PLACNET performance on a set of ten *E. coli* genomes.
(PDF)

S4 Table Molecular size of the plasmids estimated by S1-PFGE and PLACNET reconstruction in the four strains sequenced in this study.
(PDF)

S1 Text The plasmidome of *E. coli* ST131.
(PDF)

S2 Text PLACNET validation by reconstruction of finalized genomes.
(PDF)
