# Supplementary figures and images for: Plasmid Flux in Escherichia coli ST131 Sublineages, Analyzed by Plasmid Constellation Network (PLACNET), a New Method for Plasmid Reconstruction from Whole Genome Sequences
Source: PLoS Genet. 2014 Dec 18;10(12):e1004766. doi: 10.1371/journal.pgen.1004766 (PMC4270462; doi:10.1371/journal.pgen.1004766)

Figure S1

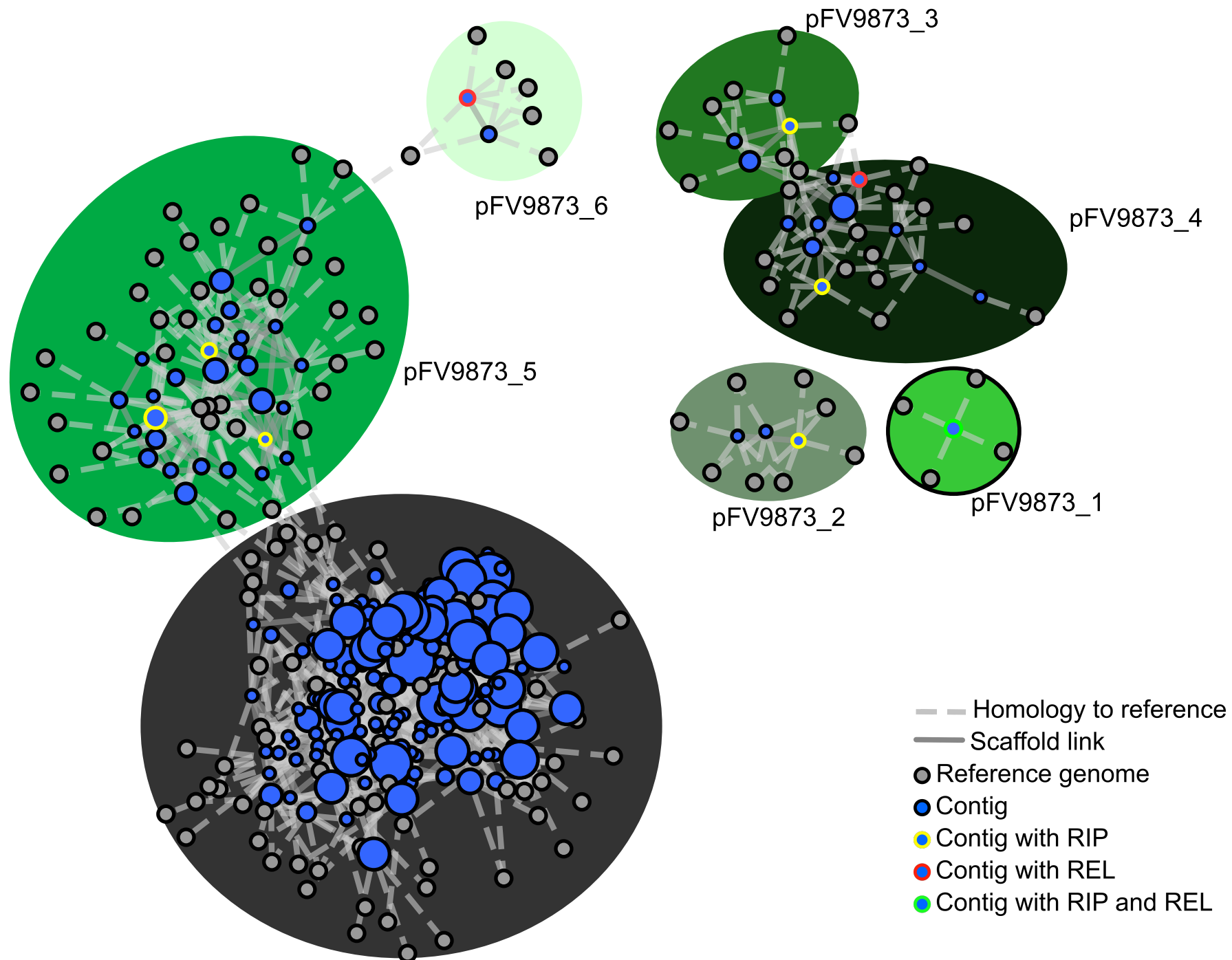

Supplement: S1 Fig — PLACNET reconstruction for FV9873 genome (E. coli ST131/H30/virotype A). A total of 262 contigs were classified in chromosome and six plasmids. The black line surrounding the pFV9873_1 plasmid (4.1 bp) indicates that it is a closed plasmid. There are not unassigned contigs. (PDF) [file pgen.1004766.s001.pdf]

Figure S2

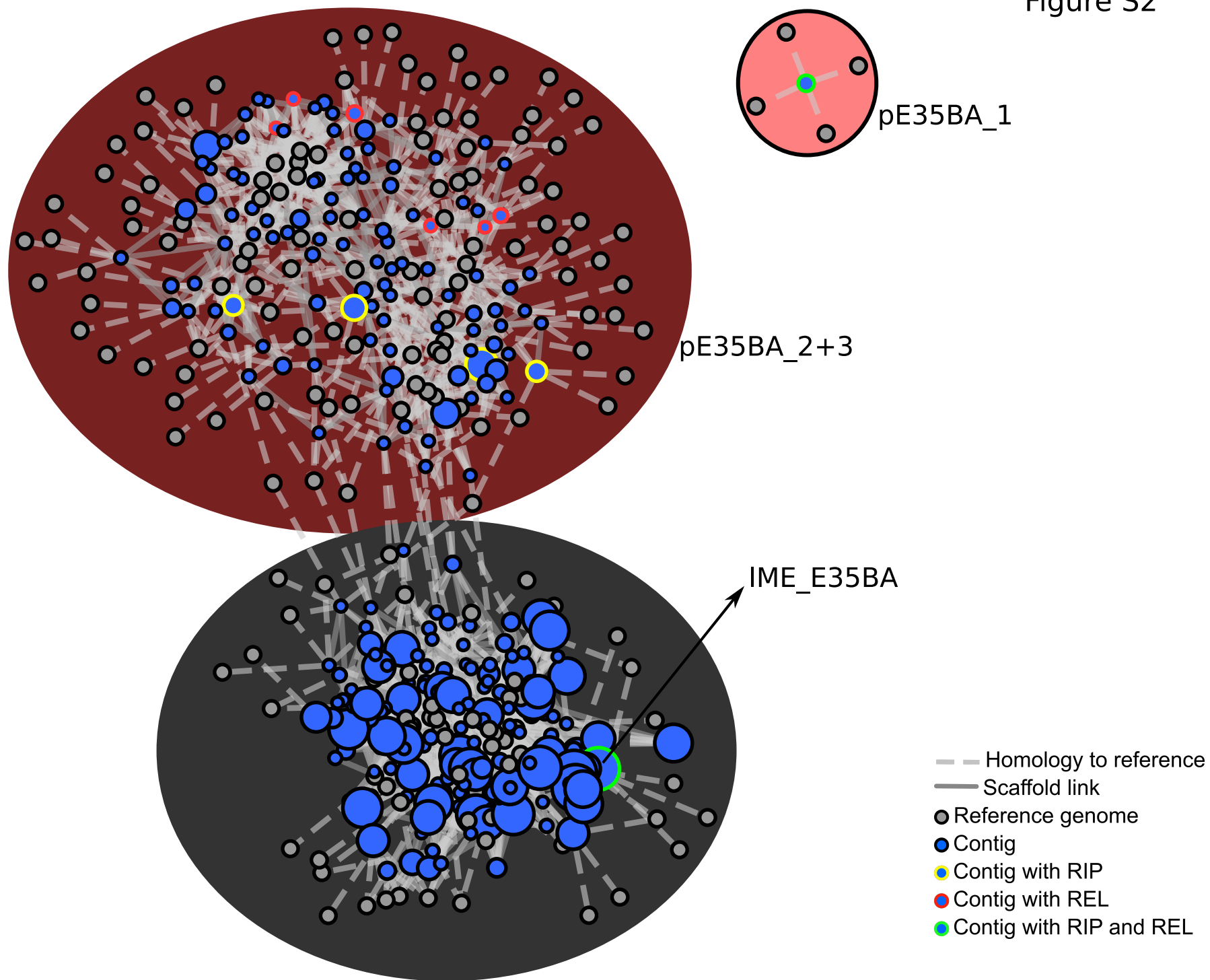

Supplement: S2 Fig — PLACNET reconstruction for E35BA genome (E.coli ST131/H30/virotype B). One 14.2 kb MOBP11 Integrative Mobilizable Element (IME) was detected in the chromosome. The pE35BA_1 plasmid (4.1 kb) is closed. There was a conflict of separation between two IncF plasmids (pE35BA_2 and pE35BA_3, total size: 211 kb). The annotation of this particular genome is limited by the quality of the assembly (many small and non-scaffolded contigs). This is the network with the highest number of contigs in our study (total: 419). No contigs remained unassigned. (PDF) [file pgen.1004766.s002.pdf]

Figure S3

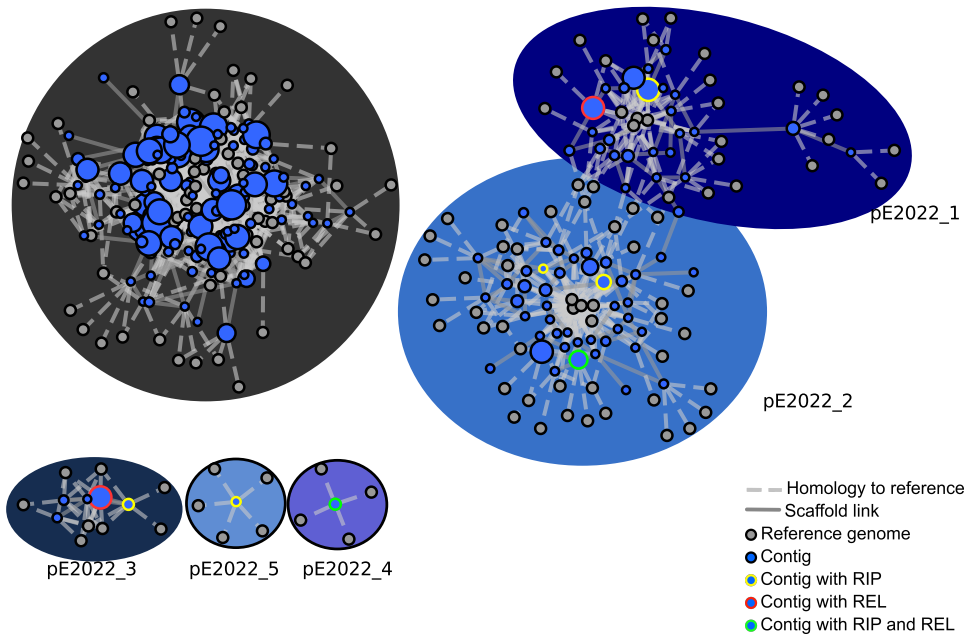

Supplement: S3 Fig — PLACNET reconstruction for E2022 genome (E. coli ST131/H30/virotype C). A total of five plasmids, two of them as closed plasmids, and the chromosome were obtained in a 346-contig network. No contigs remained unassigned. (PDF) [file pgen.1004766.s003.pdf]

Figure S4

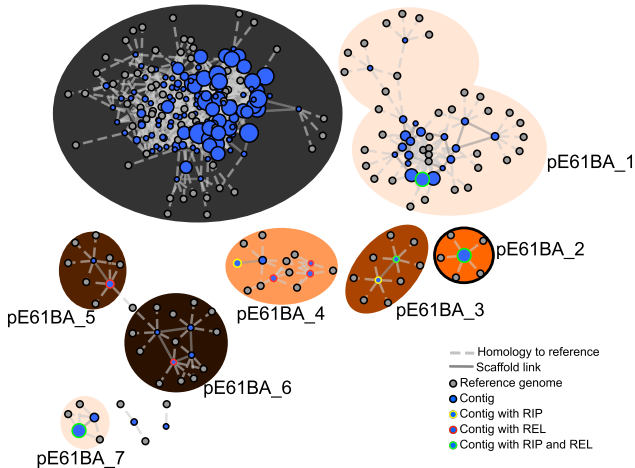

Supplement: S4 Fig — PLACNET reconstruction for E61BA genome (E. coli ST131/H324/virotype D). Seven different plasmids were obtained in the analysis. The pE61BA_2 plasmid (24.5 kb), containing a single contig, was closed. Two contigs remained unassigned, as no scaffold links were detected for them. One of them (2,953 bp) encodes for a putative DNA primase and a lytic transglycosilase, while another (1,301 bp) encodes for TrbI and TraB partial proteins. (PDF) [file pgen.1004766.s004.pdf]

Figure S5

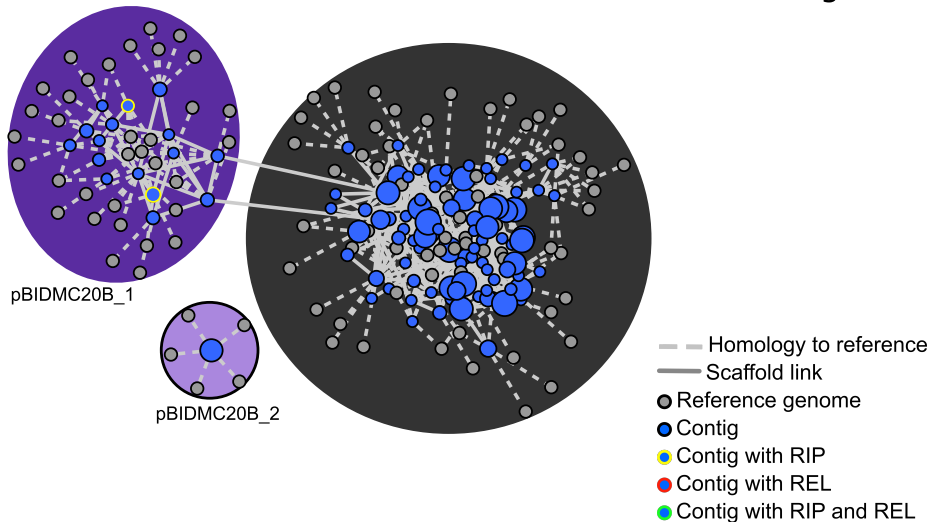

Supplement: S5 Fig — PLACNET reconstruction for BIDMC20B genome (E. coli ST131/H30/virotype C). A total of 115 contigs were fully assigned to the chromosome and two plasmids. Plasmid pBIDMC20B_2 (109 kb) appeared as a single contig that could be closed. (PDF) [file pgen.1004766.s005.pdf]

Figure S6

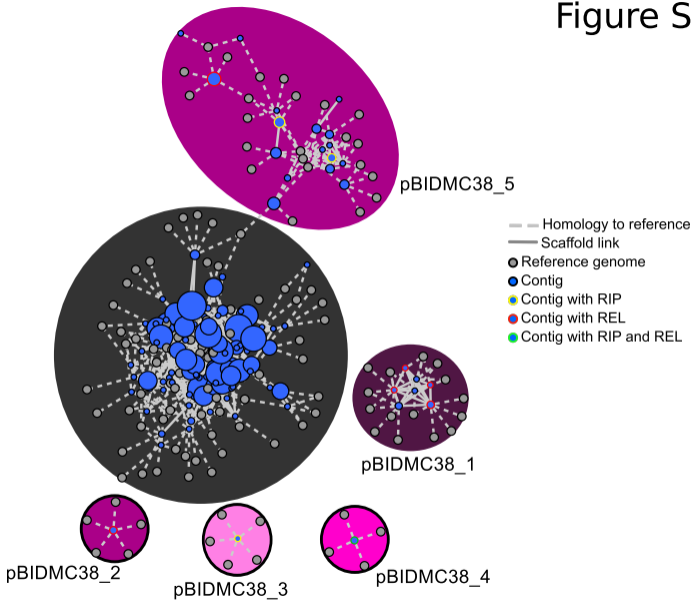

Supplement: S6 Fig — PLACNET reconstruction for BIDMC38 genome (E. coli ST131/H30/virotype A). Five plasmids were detected. Three small plasmids (1.6, 4.2 and 5.3 kb) are closed. No contigs remained unassigned. (PDF) [file pgen.1004766.s006.pdf]

Figure S7

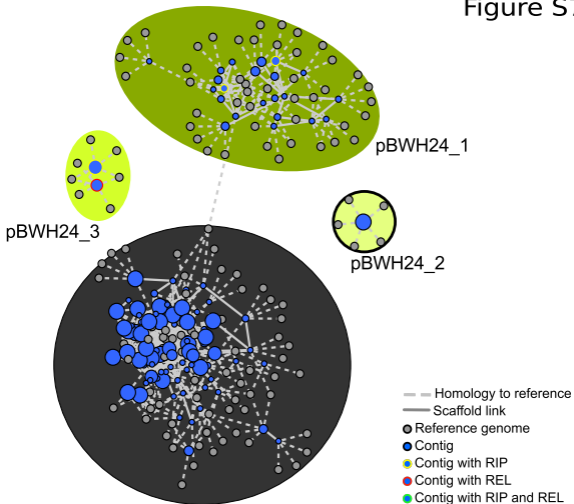

Supplement: S7 Fig — PLACNET reconstruction for BWH24 genome (E. coli ST131/H30/virotype C). Three plasmids were detected, only one of them as a closed plasmid (pBWH24_2, 109 kb). No contigs remained unassigned. (PDF) [file pgen.1004766.s007.pdf]

Figure S8

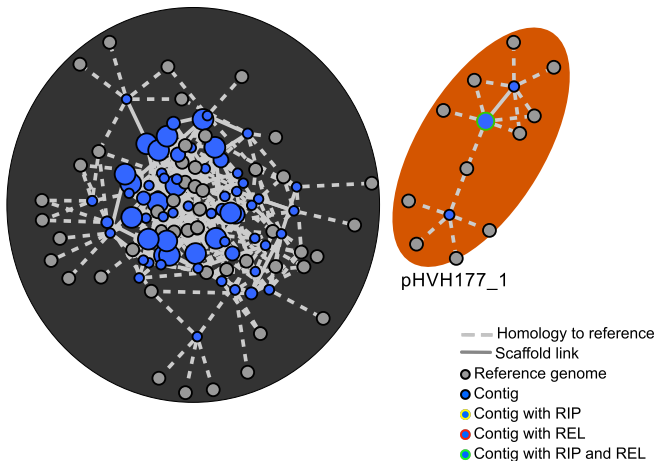

Supplement: S8 Fig — PLACNET reconstruction for HVH177 genome (E. coli ST131/H324/virotype D). Only one plasmid (pHVH177_1, 78.6 kb) composed by three contigs was detected in the HVH177 genome. No contigs remained unassigned. (PDF) [file pgen.1004766.s008.pdf]

Figure S9

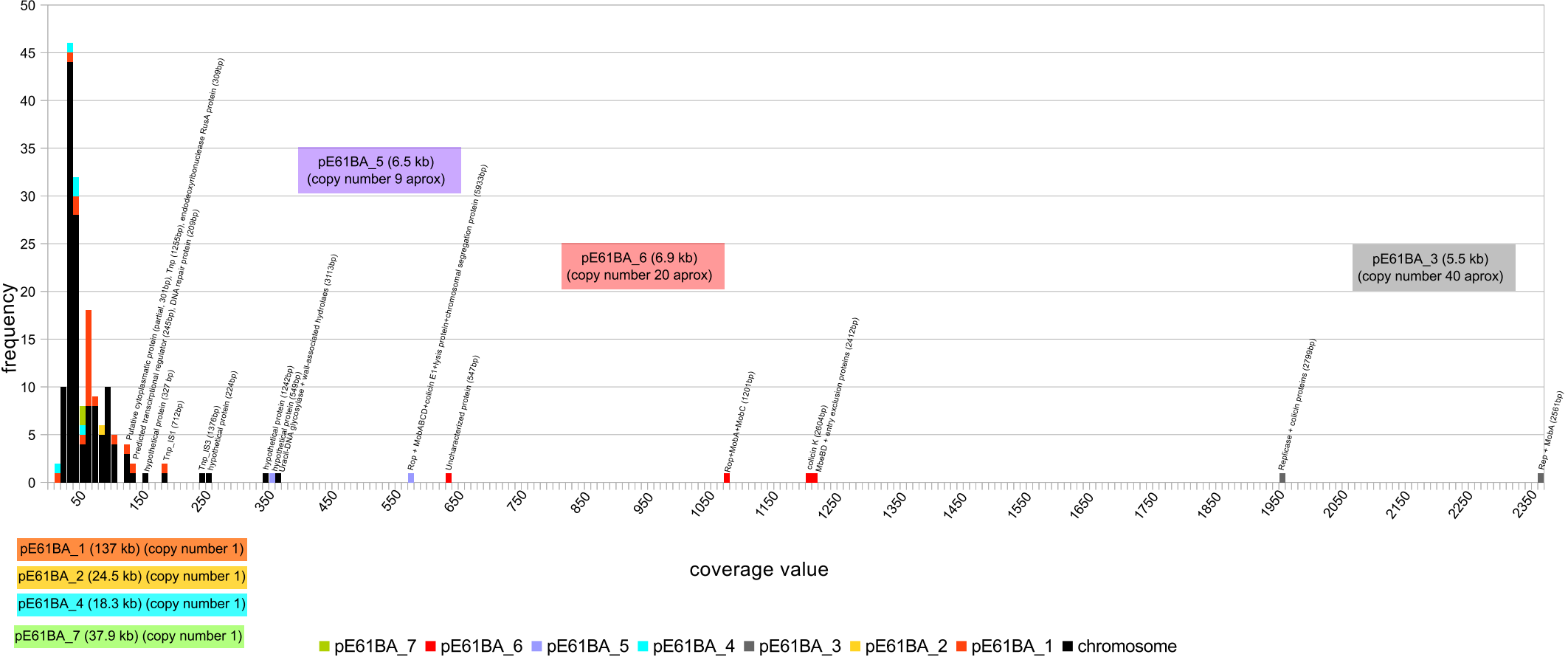

Supplement: S9 Fig — Contig coverage in E61BA genome (E. coli ST131/H324/virotype D). Contigs belonging to each plasmid are shown in different colors, according to the code below the histogram. Plasmid copy numbers are inferred from their contig coverage. Average coverage of chromosomal contigs was 56. All contigs with >2X average are named according to their predicted gene products. When there is more than one contig, annotations are separated by colons, if adequate. (PDF) [file pgen.1004766.s009.pdf]

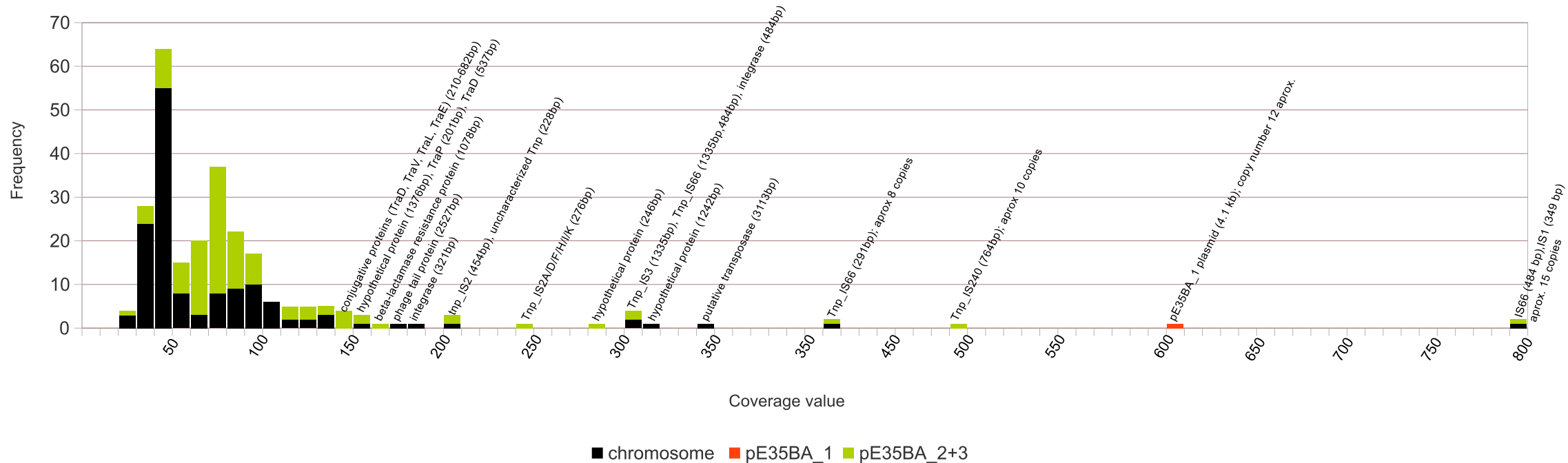

Supplement: S10 Fig — Contig coverage in E35BA genome (E.coli ST131/H30/virotype B). Contigs belonging to each plasmid are shown in different colors, according to the code below the histogram. Plasmid copy numbers are inferred from their contig coverage. Average coverage of chromosomal contigs was 55. All contigs with >2X average were named according to their predicted gene products. If more than one contig coincided within the same coverage section, annotations of the individual contigs were separated by colons. As is shown in the histogram, contigs corresponging to the MOBF12/IncF plasmids pE35BA_2+3 (in green color) show similar coverage than the chromosome, which indicates that both IncF plasmids have the same copy number than the chromosome. (PDF) [file pgen.1004766.s010.pdf]

pSSAP03302A

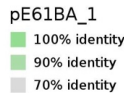

Figure S11B

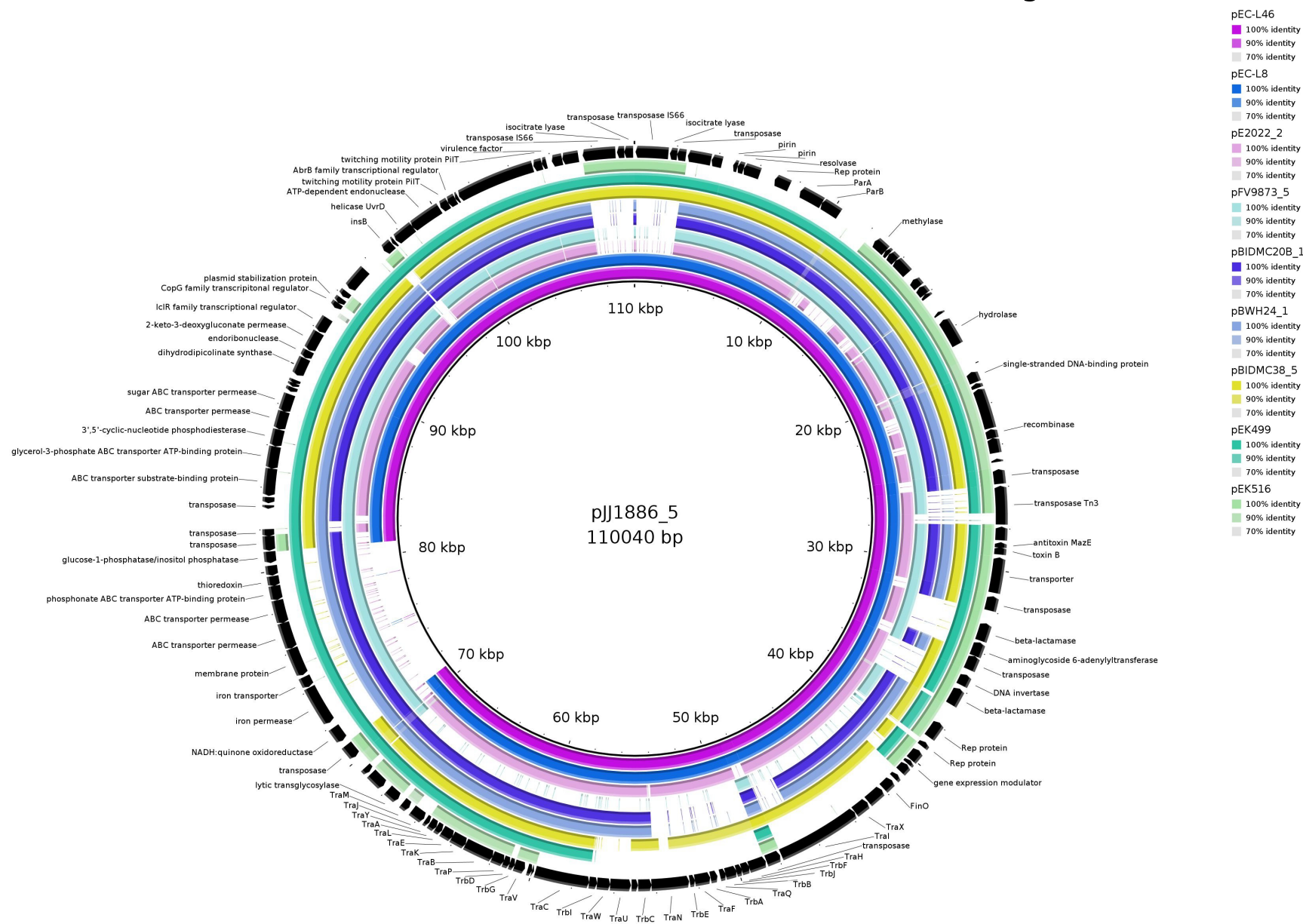

Figure S11C

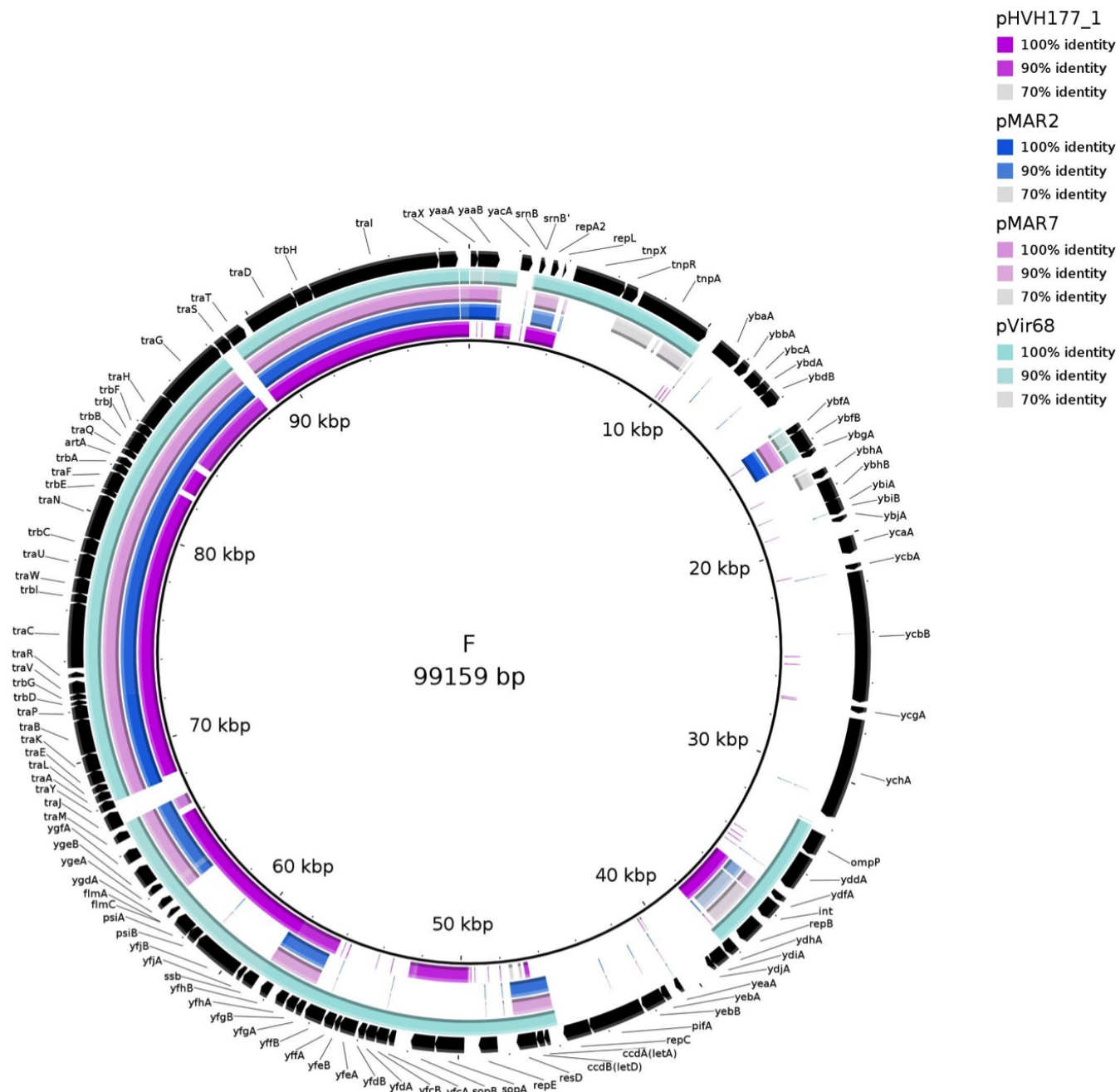

Supplement: S11 Fig — BRIG comparative analysis of MOBF12/IncF plasmids. These plasmids were subdivided in four groups according to Fig. 5 inset. S11A: Group I. Plasmid pJIE186-2 is used as the reference for the BRIG comparison. S11B: Group II. Plasmid pJJ1886-5 is the inner reference. S11C: Plasmid F is used as a reference. S11D: Plasmid pECSF1 is used as a reference. (PDF) [file pgen.1004766.s011.pdf]

Figure S12A

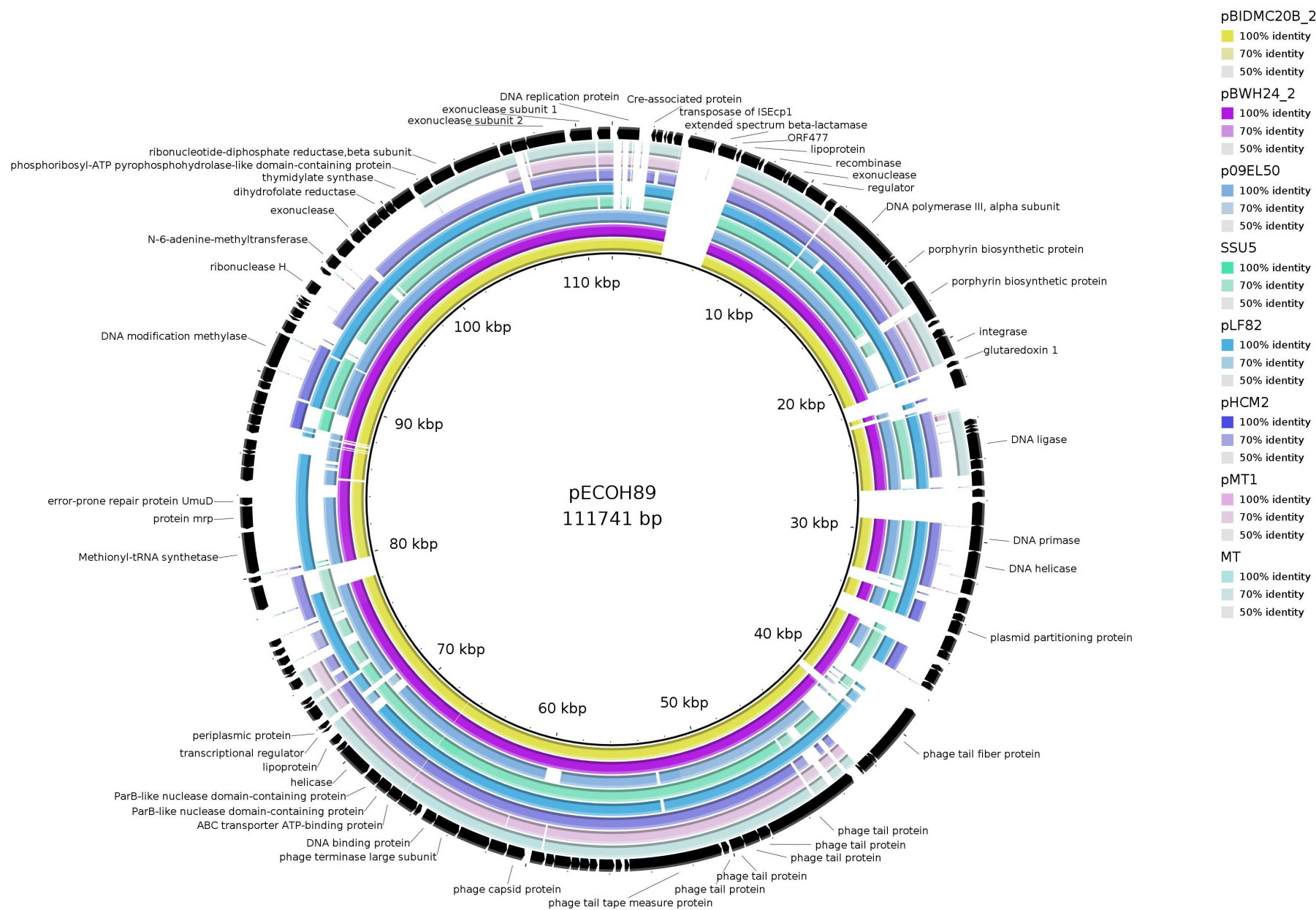

Figure S12B

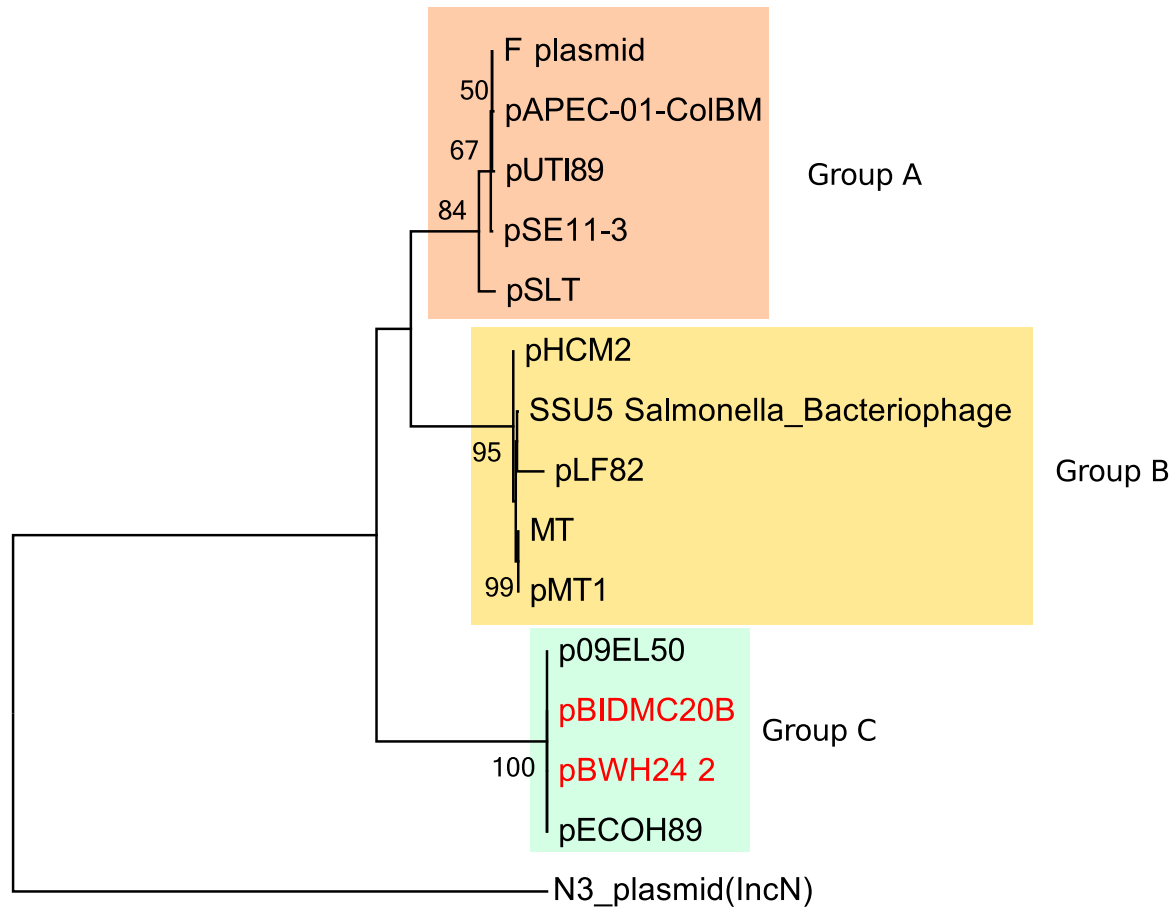

Supplement: S12 Fig — Comparative analysis of phage-related/RepFIB plasmids. S12A: BRIG representation using the 111kb plasmid pECOH89 as reference. S12B: Phylogenetic analysis of RepFIB family of RIP proteins. RaxML software (v.7.2.8) was used to infer the Maximum Likelihood tree and MEGA5.2.2 to represent the result. Bootstrap values for 100 replicates are indicated. The tree was rooted with the RepFIB protein of the IncN plasmid N3. (PDF) [file pgen.1004766.s012.pdf]

Figure S14A

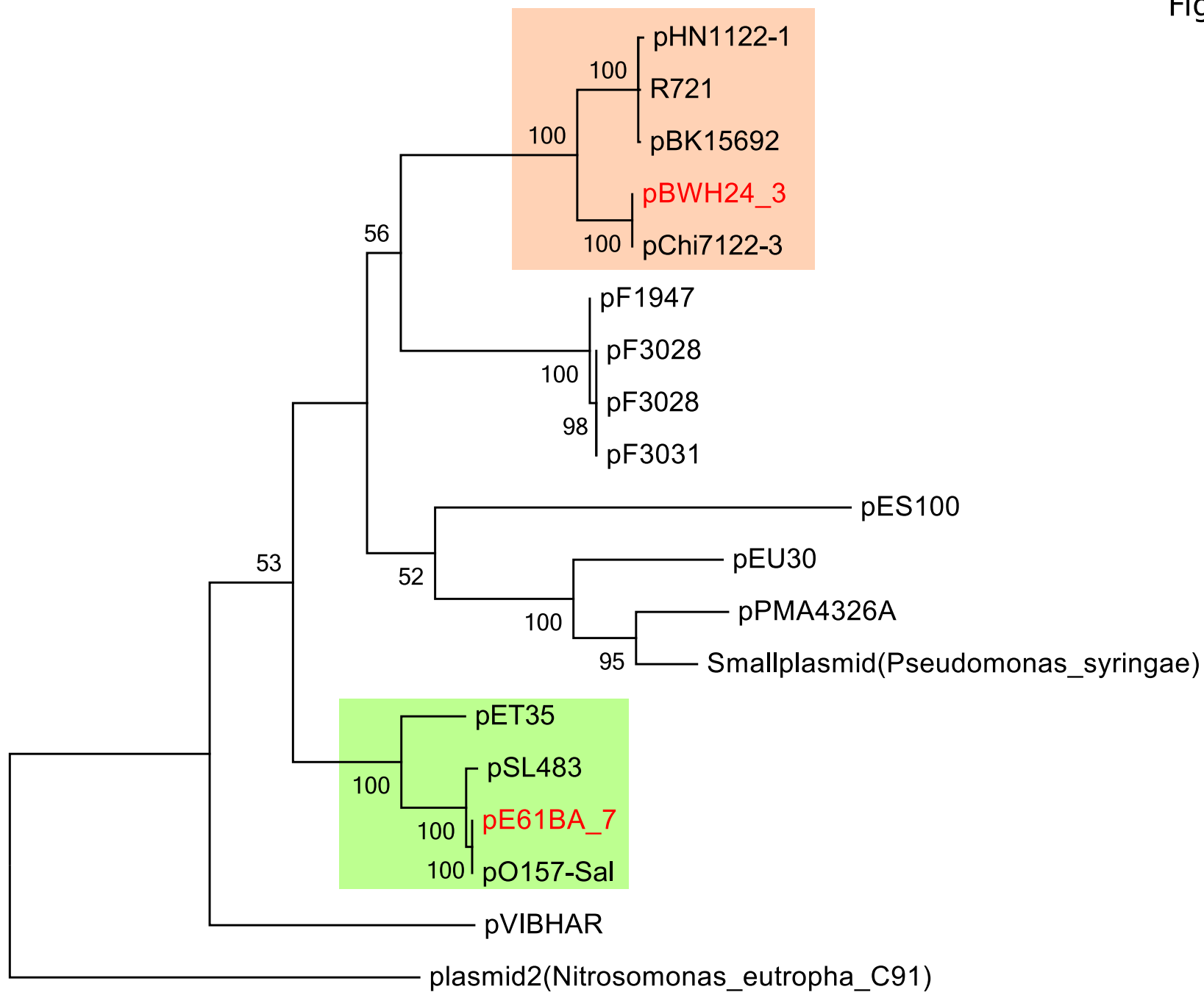

0.1

# Figure S14B

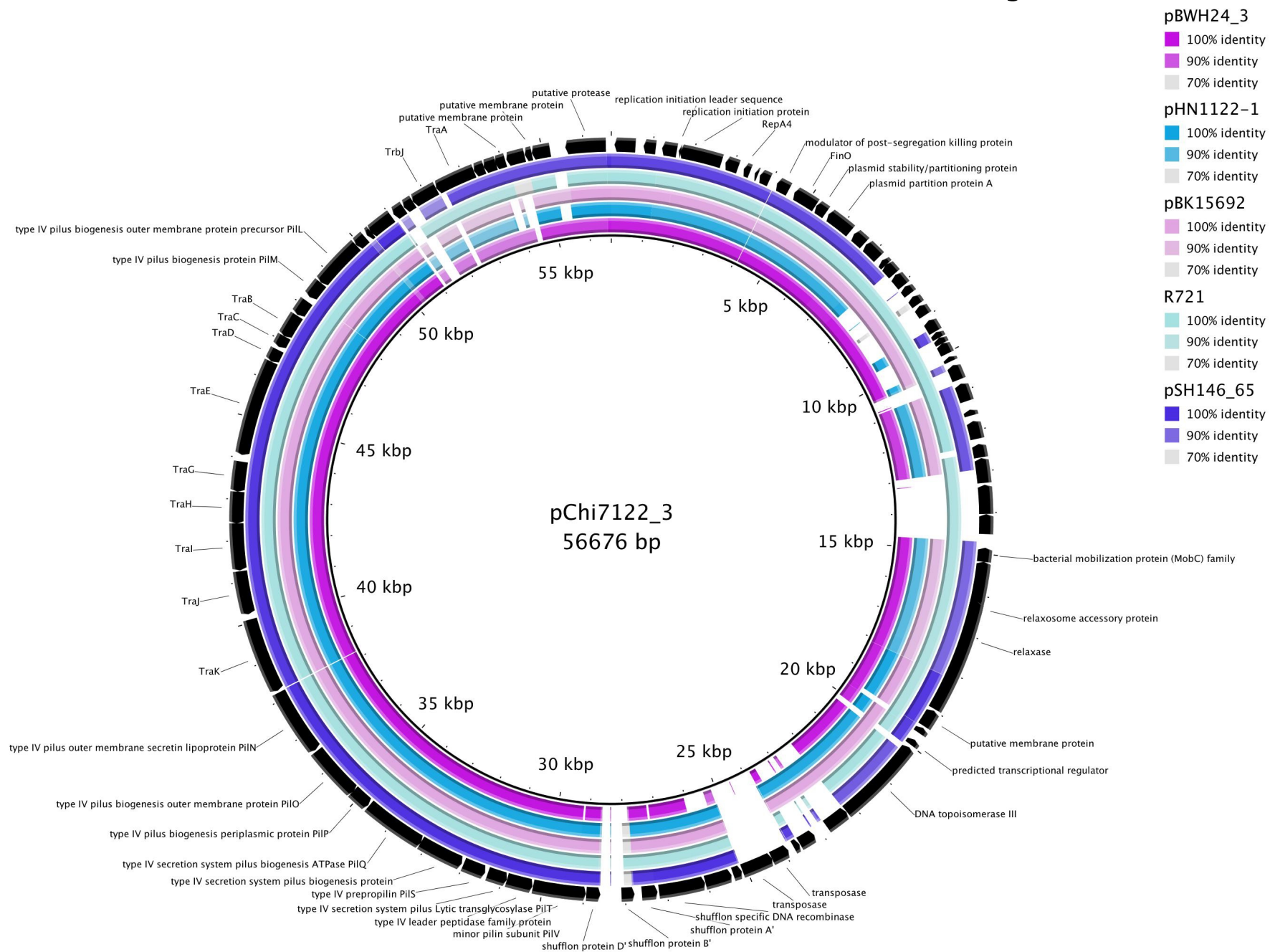

Figure S14C

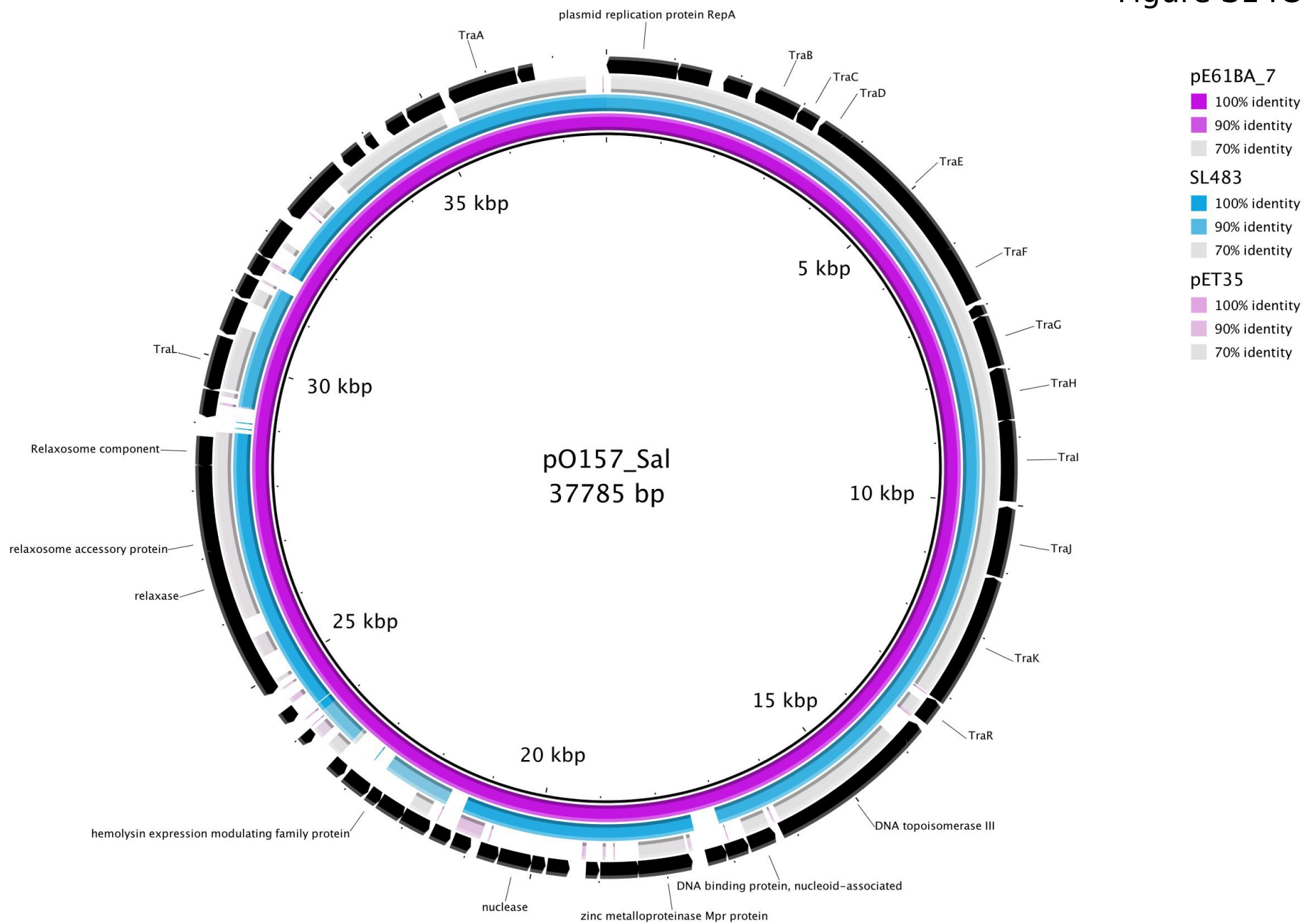

Supplement: S14 Fig — Comparative analysis of MOBP6/IncI2 plasmids. S14A: Phylogenetic tree of MOBP6 REL proteins, calculated as in S12B Fig. The tree was rooted with MOBP6 REL of Plasmid2 from Nitrosomonas eutropha C91. S14B: BRIG comparative analysis of pBWH24_3 plasmid, using pChi7122_3 as reference. S14C: BRIG comparative analysis of pE61BA_7 plasmid, using pO157_Sal as inner reference. (PDF) [file pgen.1004766.s014.pdf]

Figure S15A

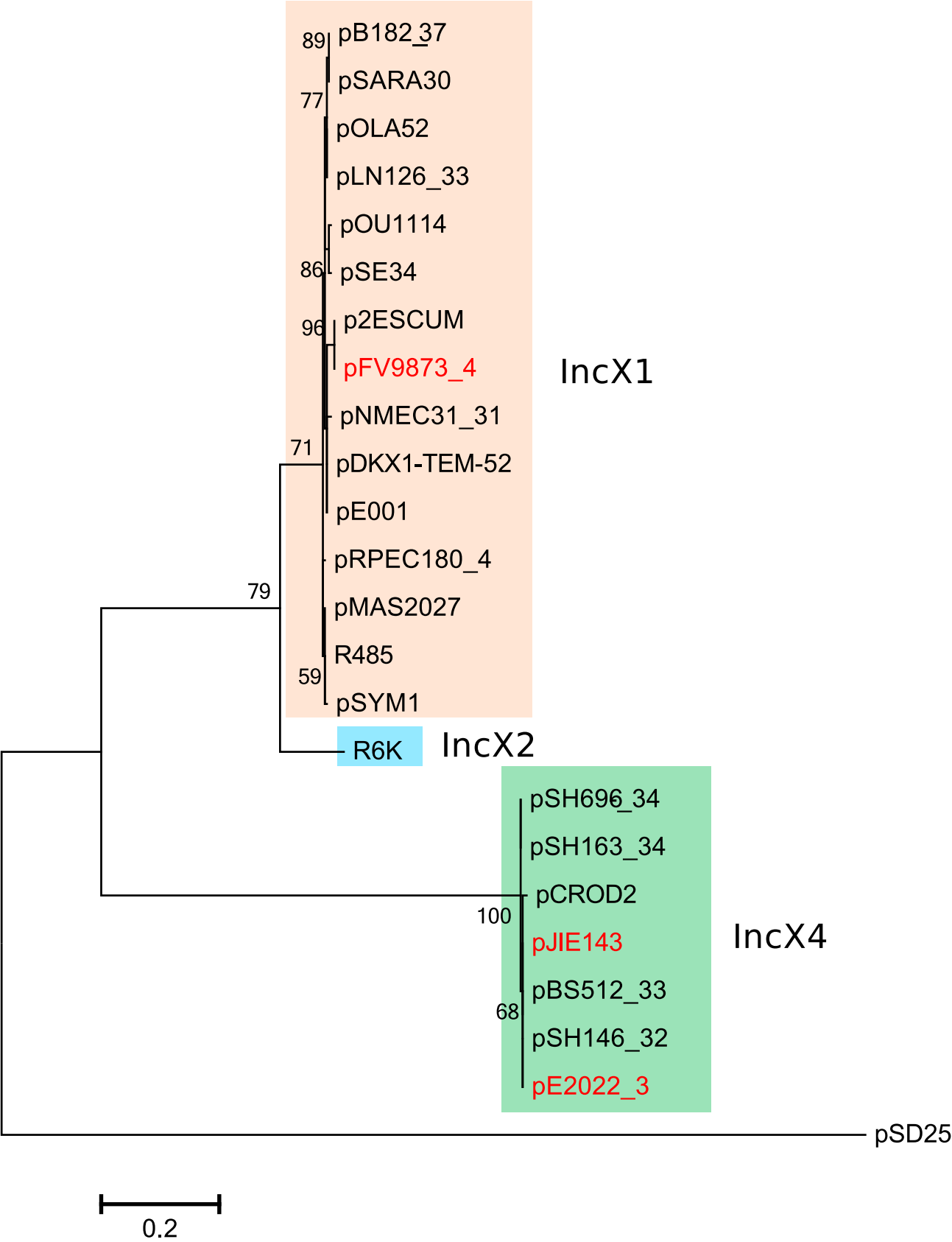

Figure S15B

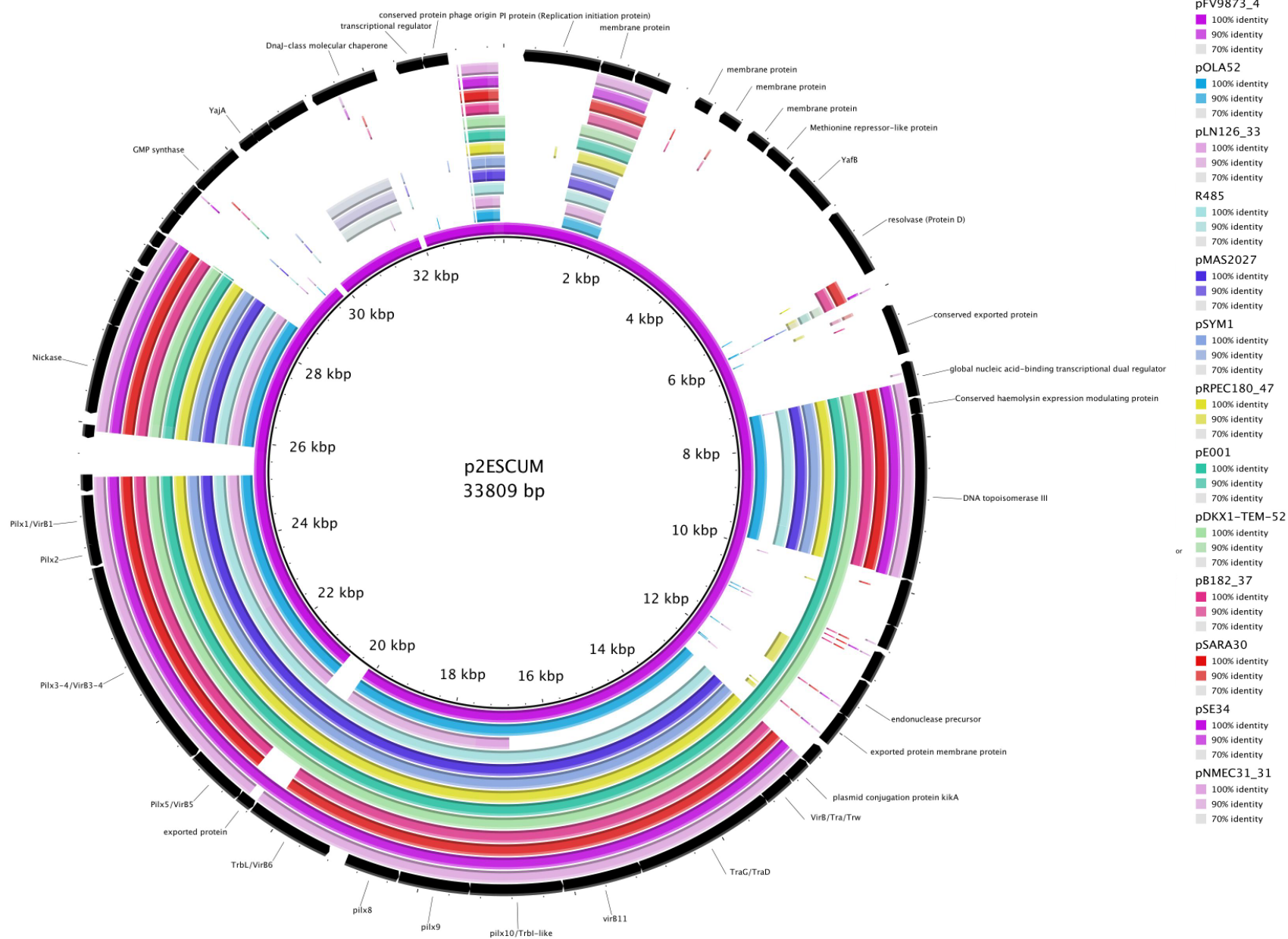

Figure S15C

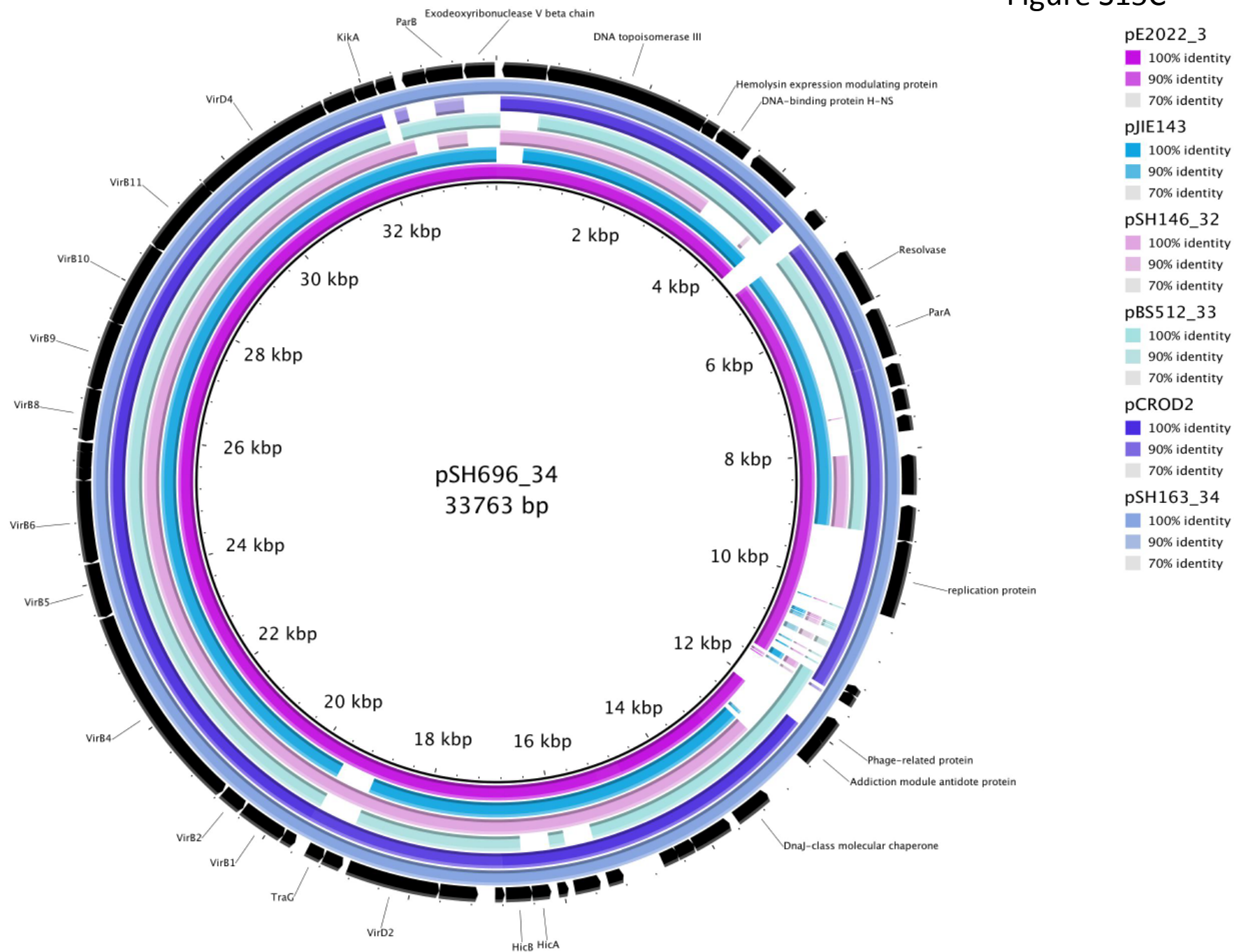

Supplement: S15 Fig — Comparative analysis of MOBP3/IncX plasmids. S15A: Phylogenetic tree of MOBP3 REL proteins, calculated as in S12B Fig. The tree was rooted with VirD2_pSD25 (MOBP2 subfamily). ST131 plasmids are shown in red. IncX subgroups are indicated in different color backgrounds. S15B: BRIG comparative analysis of IncX1 plasmids using p2ESCUM as a reference. S15C: BRIG comparative analysis of IncX4-like plasmids using pSH696_34 as reference. (PDF) [file pgen.1004766.s015.pdf]

Figure S16A

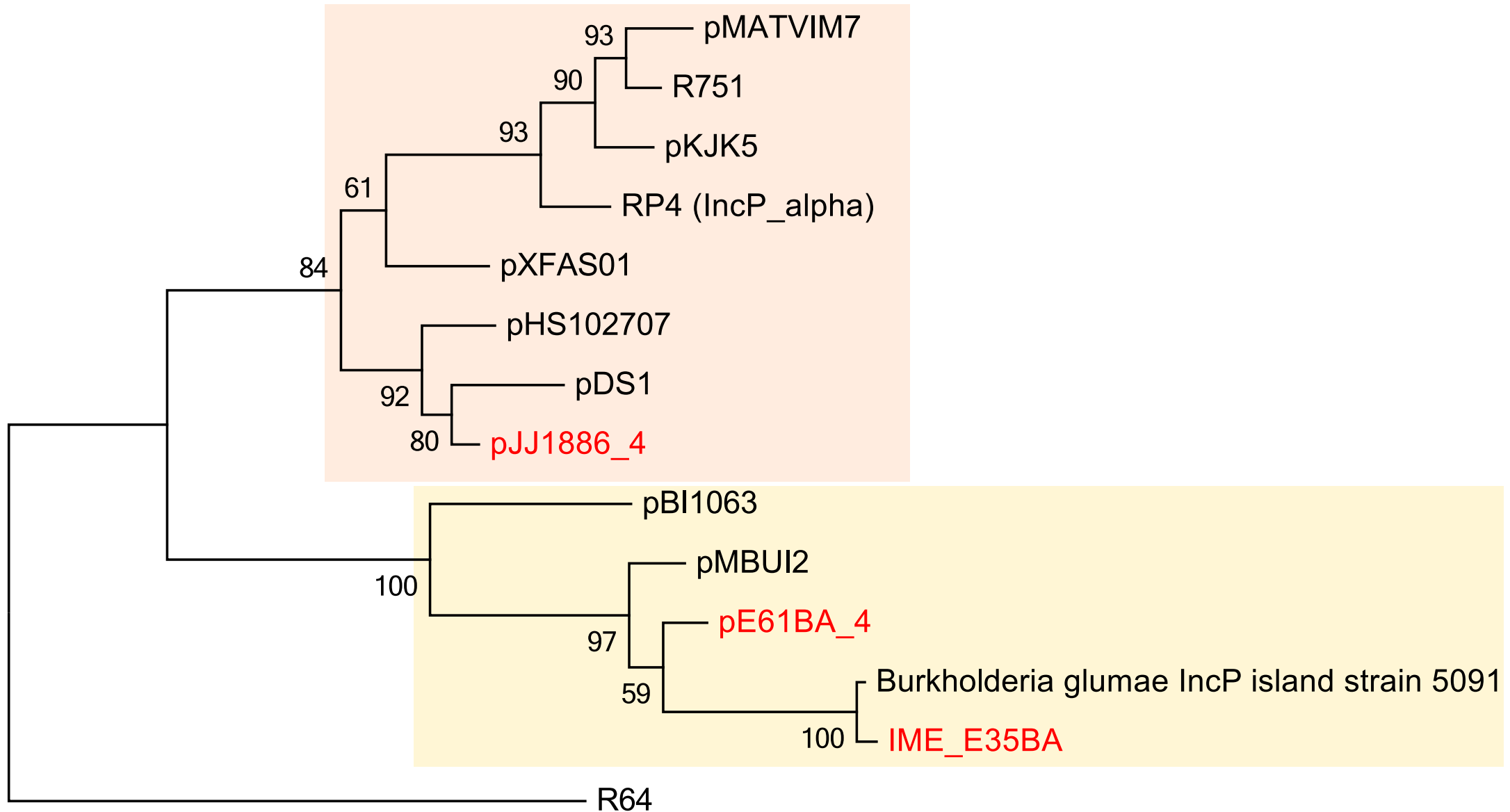

0.1

Figure S16B

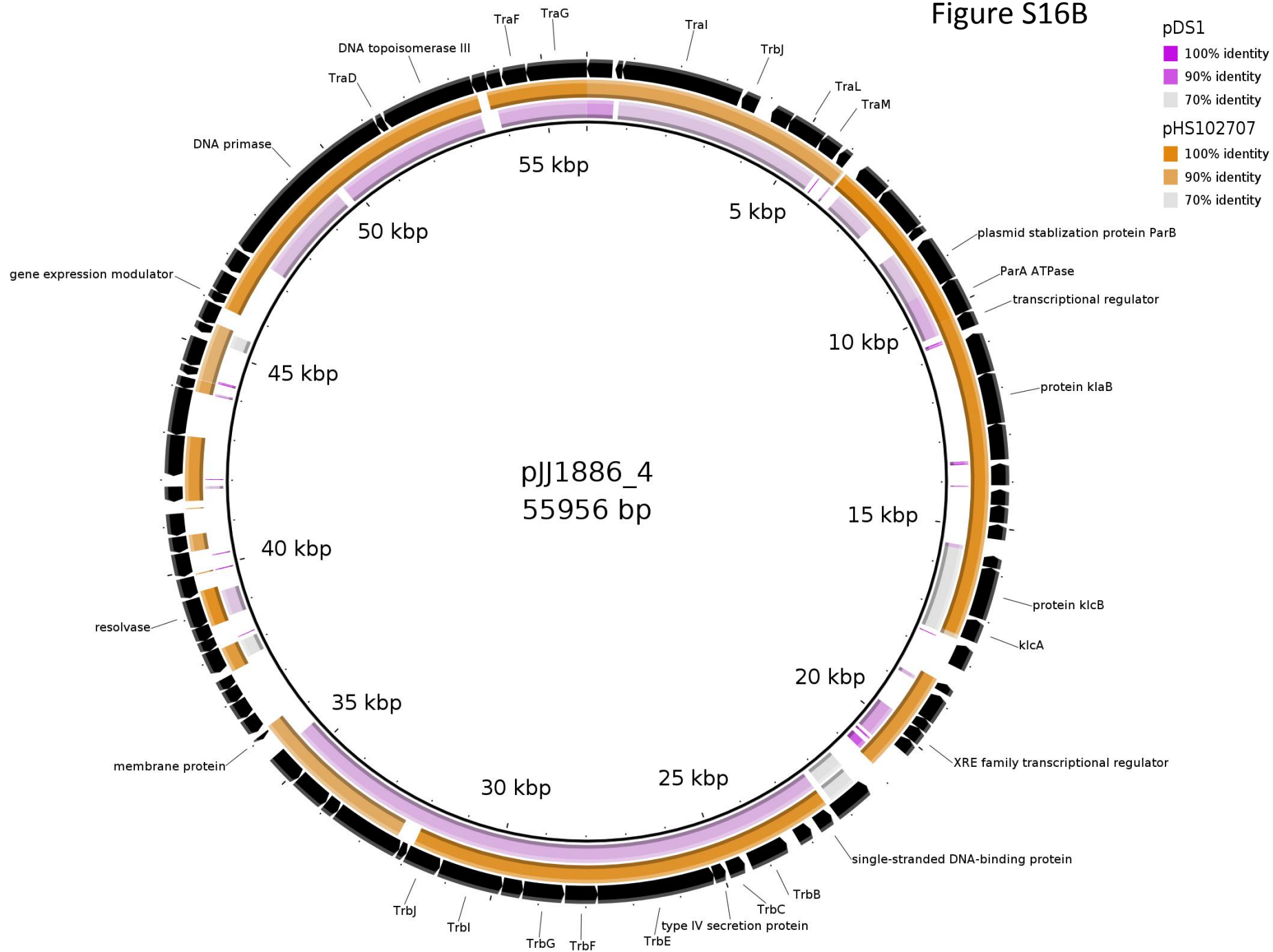

Figure S16C

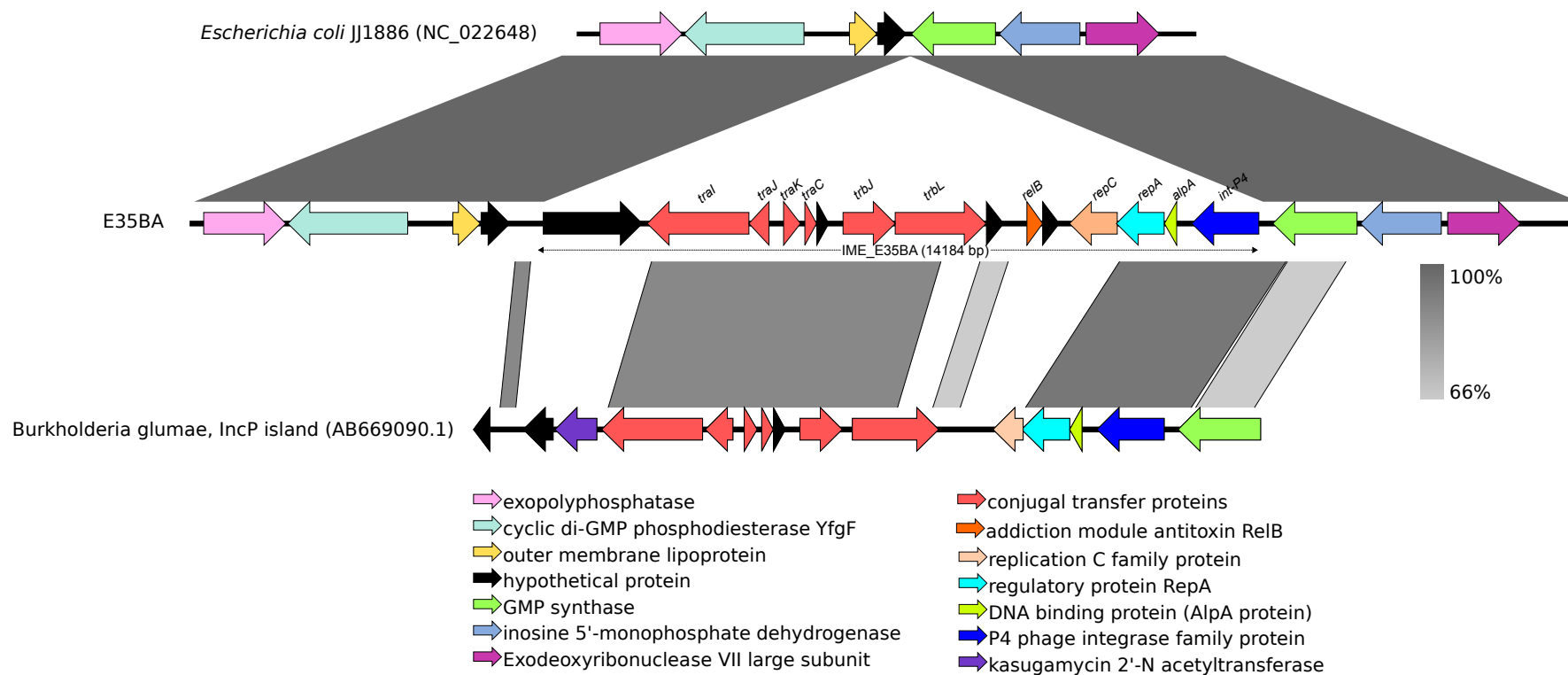

Supplement: S16 Fig — Comparative analysis of MOBP11/IncP plasmids. S16A: Phylogenetic tree of MOBP11 REL proteins, calculated as in S12B Fig. The tree was rooted with NikB_R64 (MOBP12 subfamily). ST131 plasmids are colored in red. Two clearly separated groups are colored. S16B: BRIG comparative analysis of IncP1 plasmids using pJJ1886_4 as a reference. S16C: Comparison of JJ1886 and E35BA genomes, showing the genetic map of the inserted IME_E35BA, and its homology to Bukholderia glumae IncP island. The figure was drawn with EasyFig [75]. Specific genes are specifically colored according to the code in the lower part of the figure. (PDF) [file pgen.1004766.s016.pdf]

Figure S17A

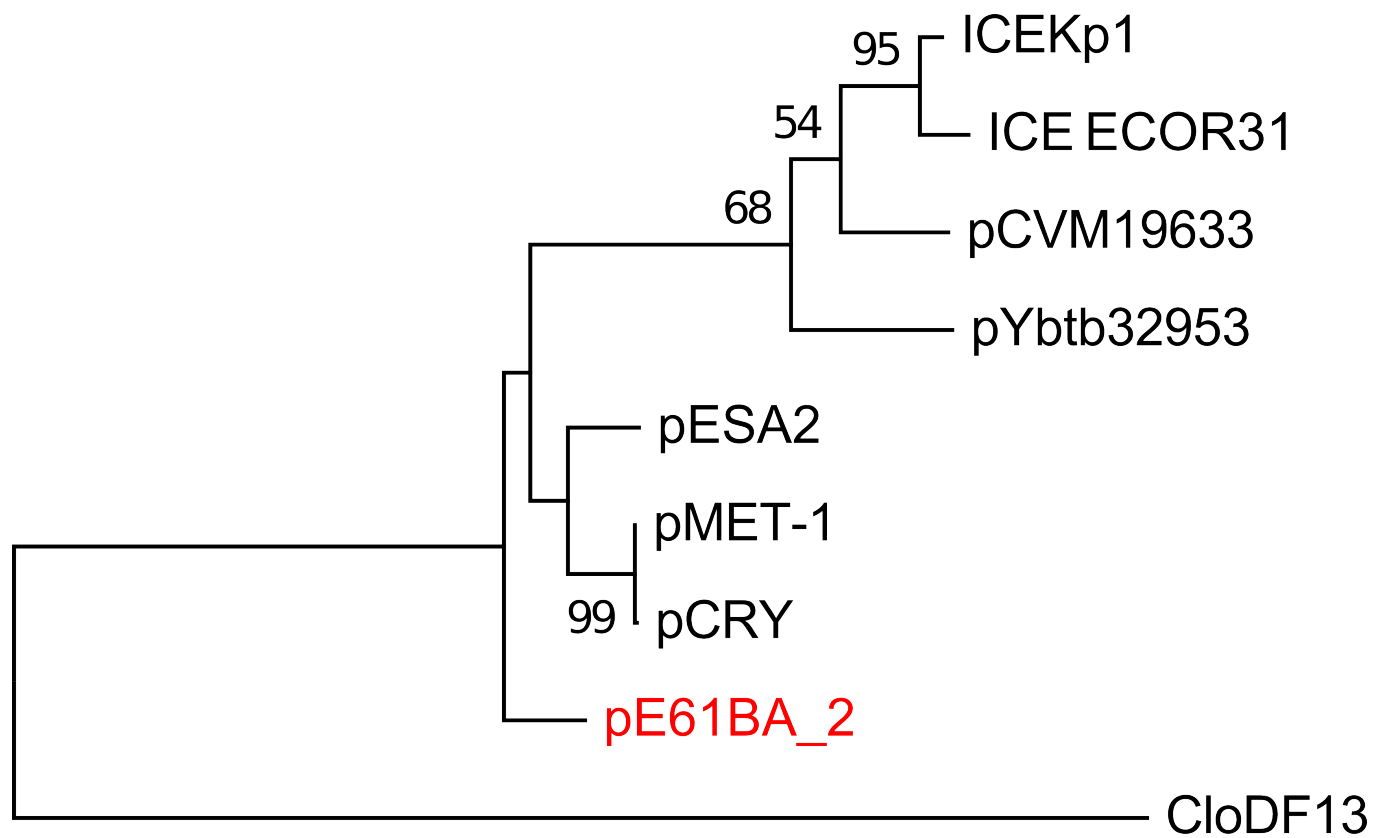

H  
0.1

Figure S17B

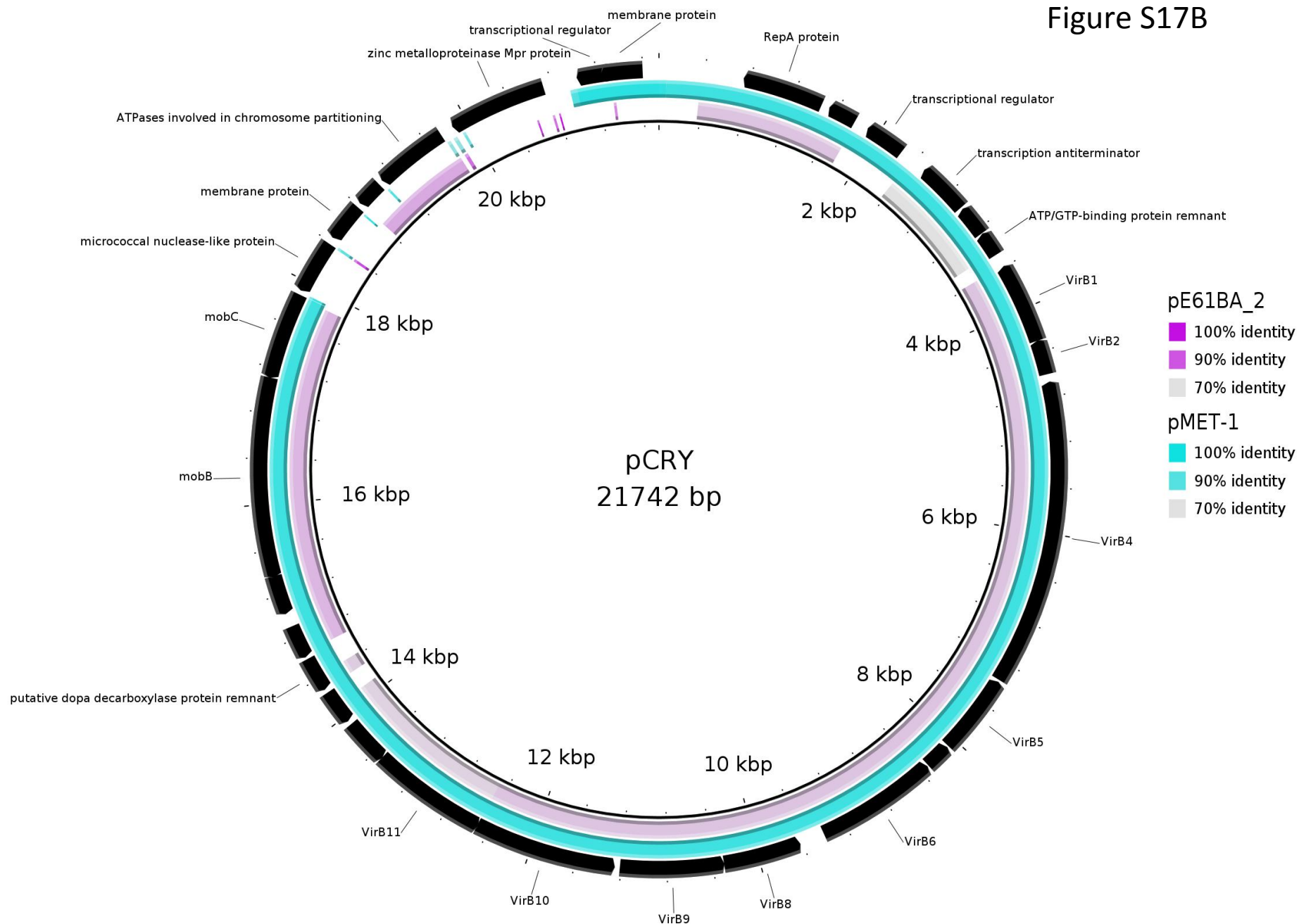

Supplement: S17 Fig — Comparative analysis of MOBC12 plasmid. S17A: Phylogenetic tree of MOBC12 REL proteins, calculated as in S12B Fig. The tree was rooted with MobC_CloDF13 (MOBC11 subfamily). S17B: BRIG comparative analysis of MOBC12 plasmids using pCRY as a reference. (PDF) [file pgen.1004766.s017.pdf]

Figure S18A

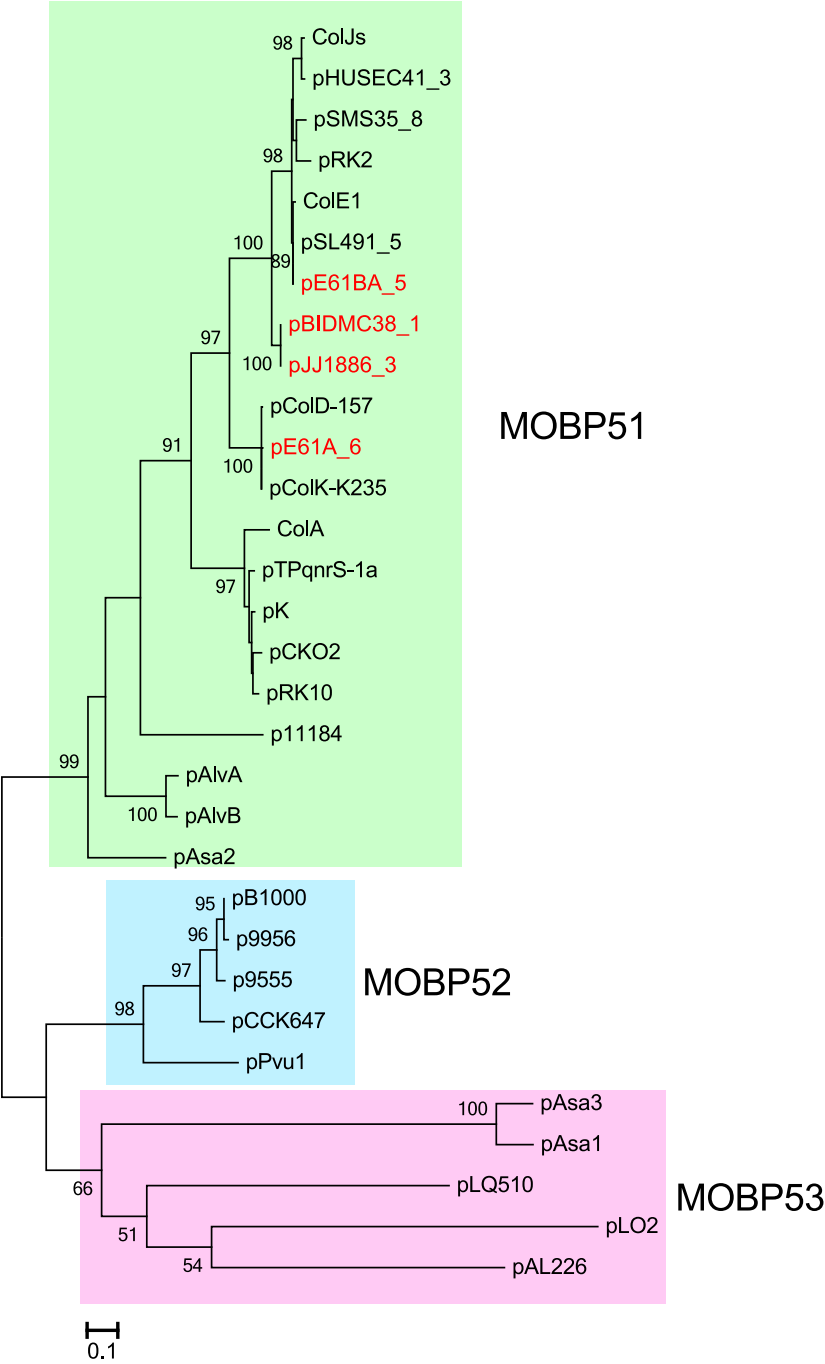

Figure S18B

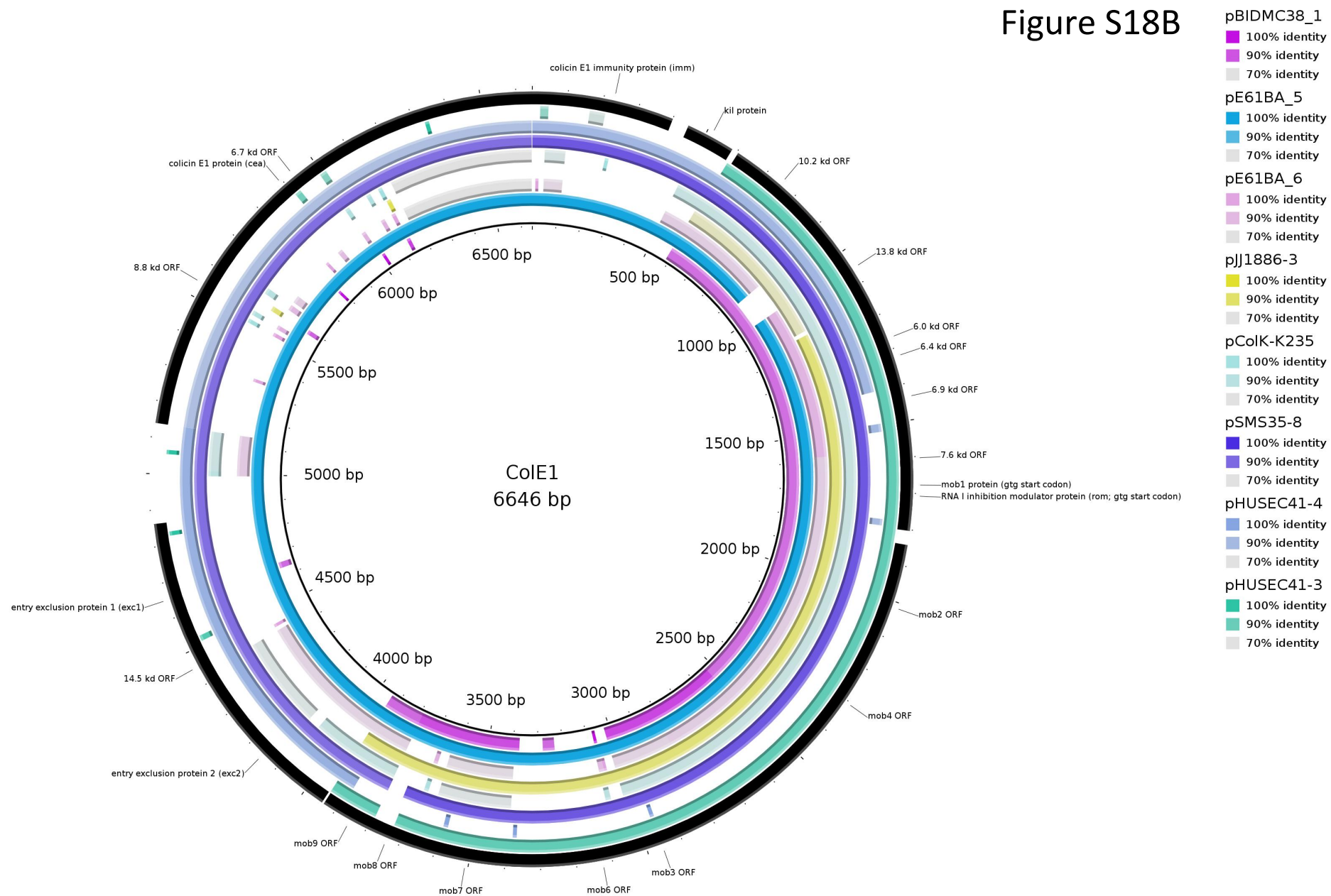

Figure S18C

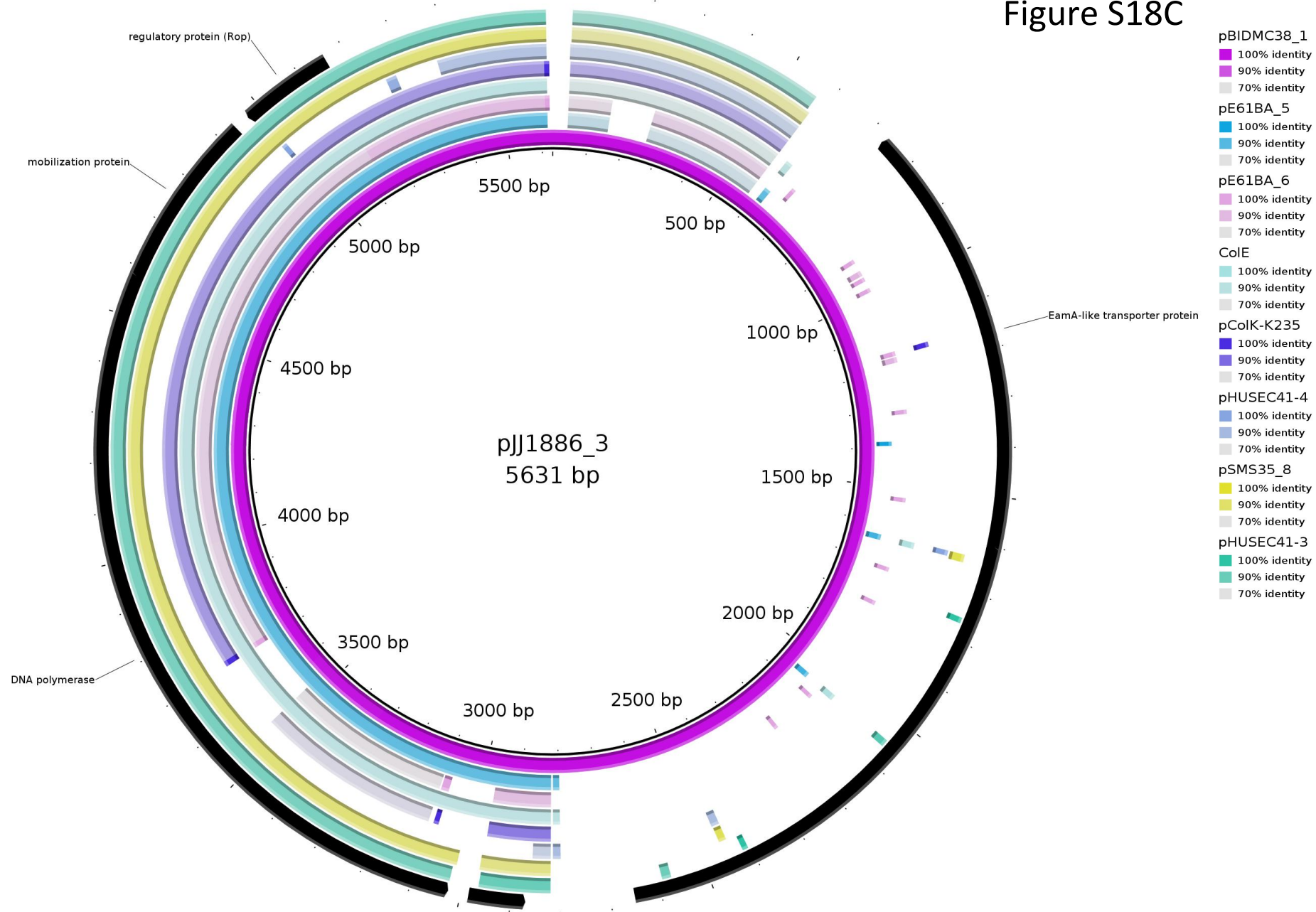

Figure S18D

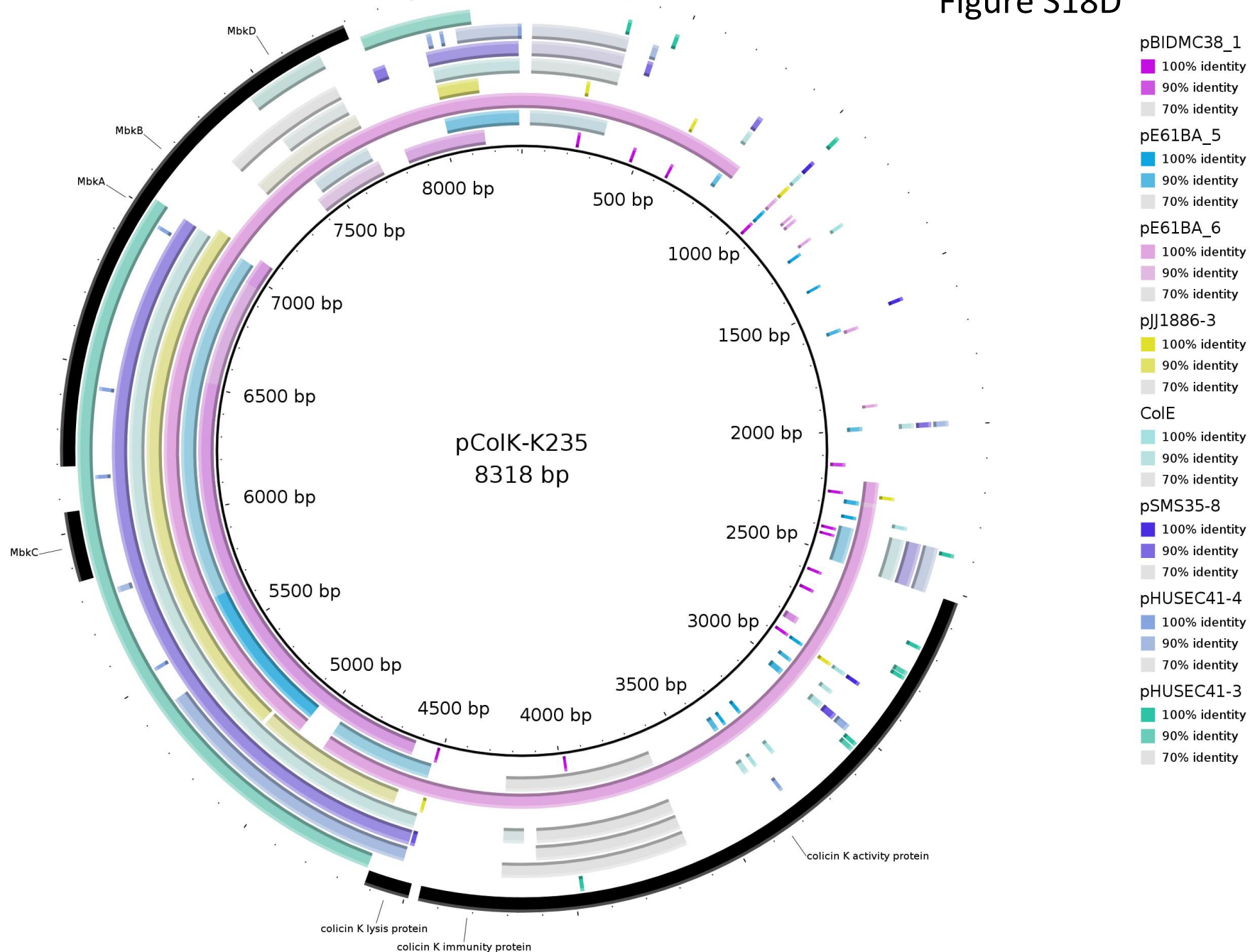

Supplement: S18 Fig — Comparative analysis of MOBP5/ColE1-like plasmids. S18A: Phylogenetic tree of MOBP5 REL proteins, calculated as in Fig SF12B. S18B, C and D: BRIG comparative analysis of MOBP5 plasmids using ColE1 (SF18B), pJJ1886_3 (SF18C) and pColK-K235 (SF18D) as references. (PDF) [file pgen.1004766.s018.pdf]

Figure S19A

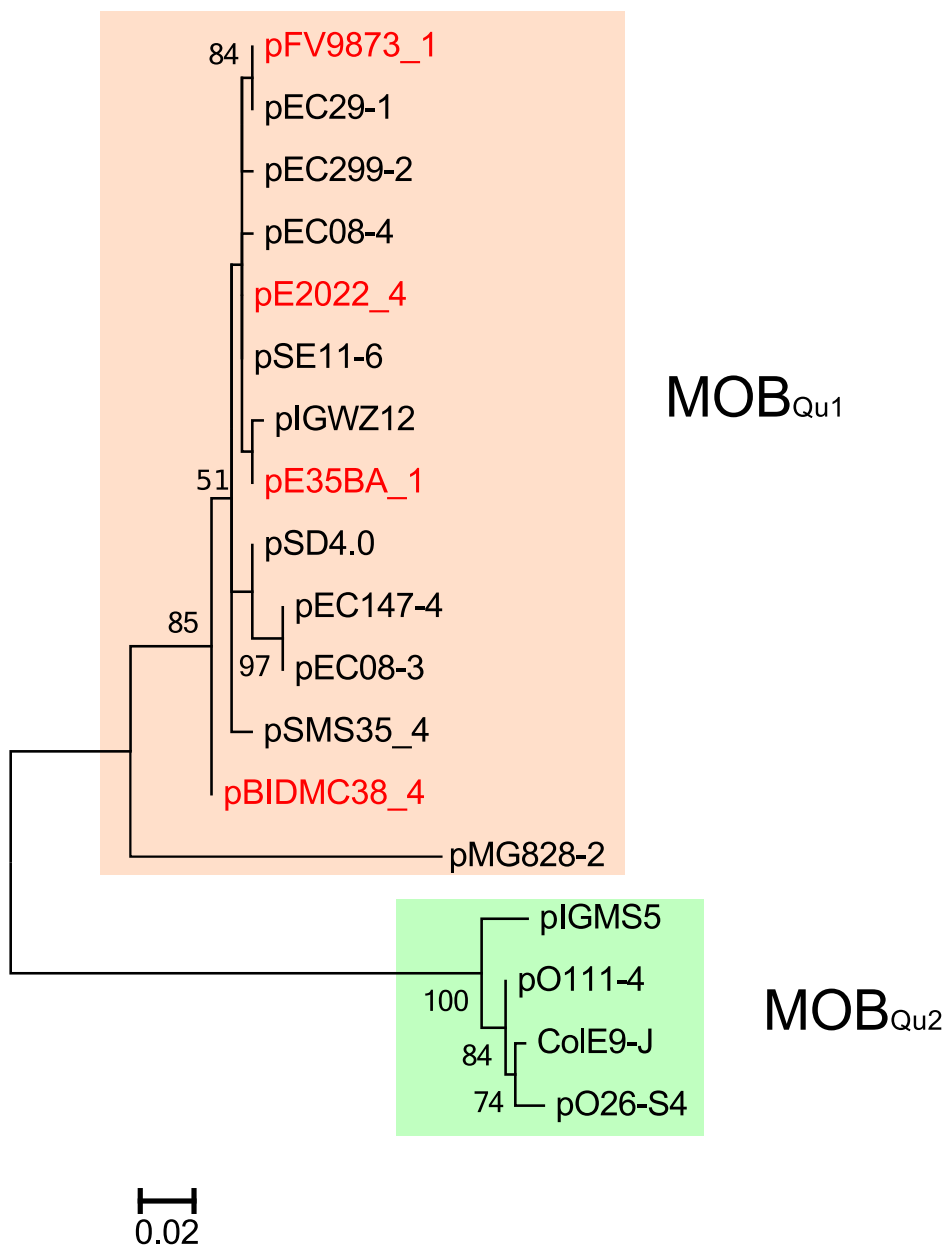

Figure S19B

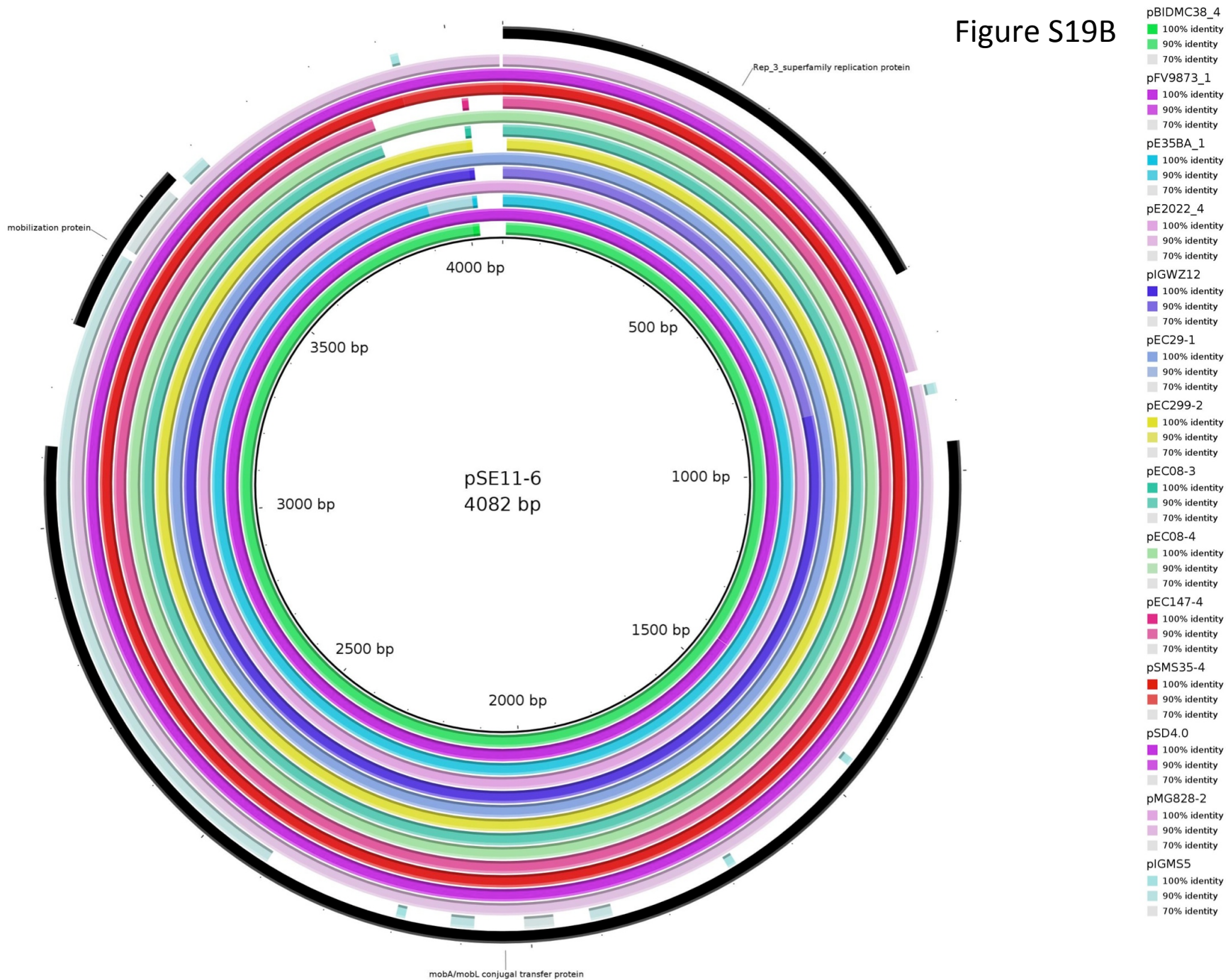

Supplement: S19 Fig — Comparative analysis of MOBQu plasmids. S19A: Phylogenetic tree of MOBQu REL proteins, calculated as in Fig SF12B. The tree was rooted with the MOBQu2 subfamily. ST131 plasmids are colored in red. Different color backgrounds are used to represent MOBQu1, where ST131 MOBQu plasmids are located, and MOBQu2 branches. S19B: BRIG comparative analysis of MOBQu plasmids using pSE11-6 as a reference. (PDF) [file pgen.1004766.s019.pdf]

Figure S20A

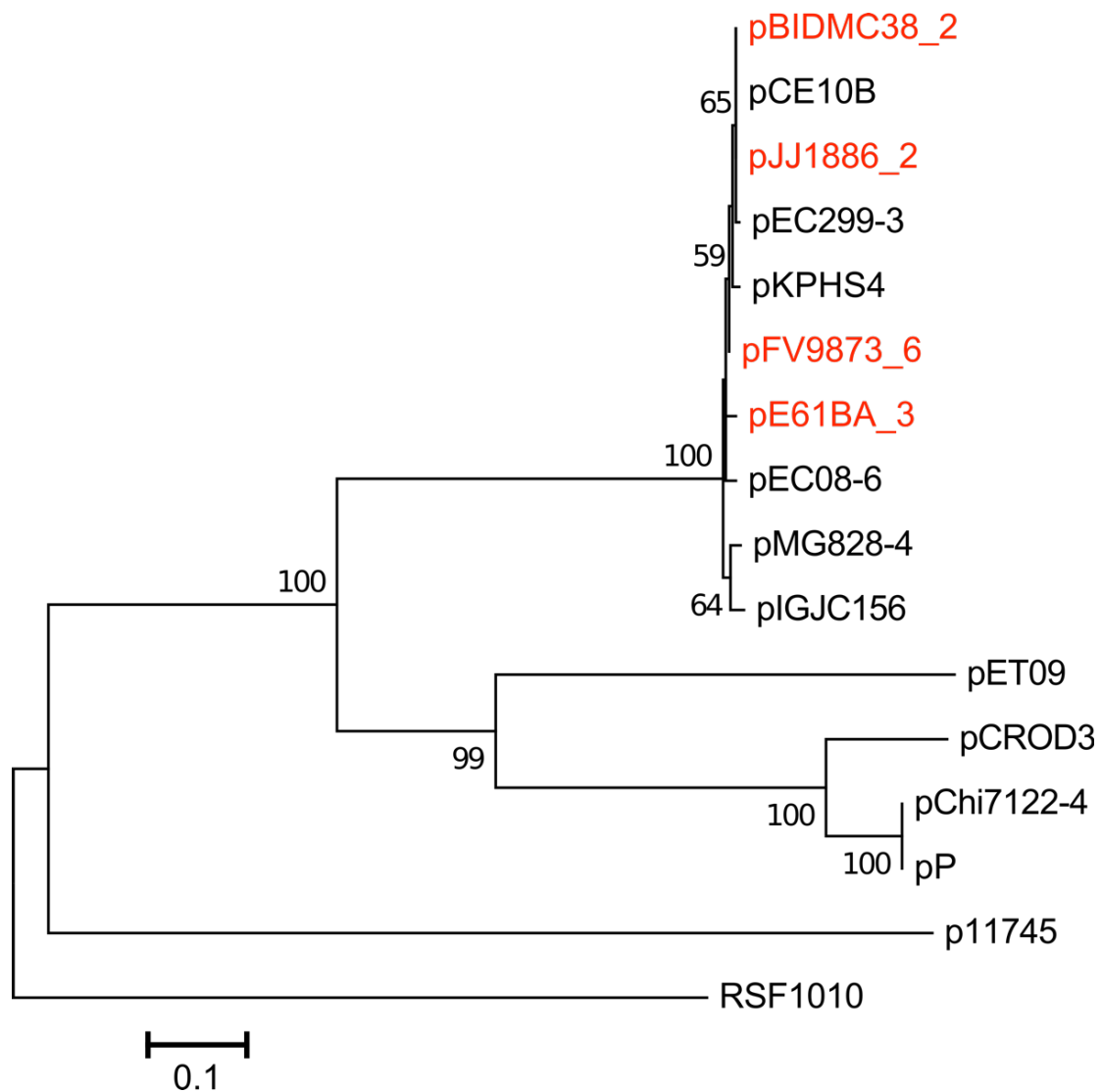

Figure S20B

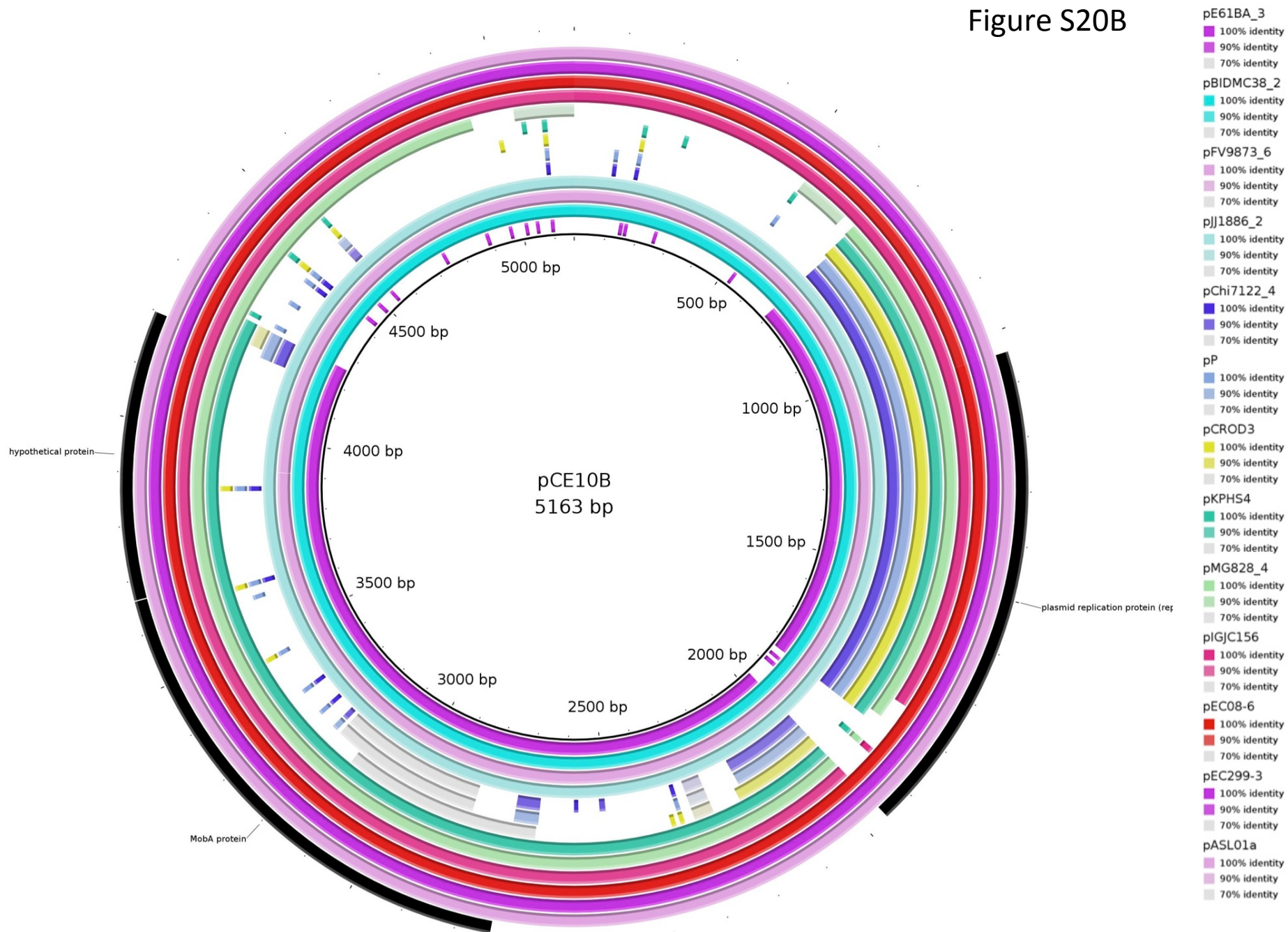

Supplement: S20 Fig — Comparative analysis of MOBQ12 plasmids. S20A: Phylogenetic tree of MOBQ12 REL proteins, calculated as in Fig SF12B. The tree was rooted with MobA_RSF1010 (MOBQ11 subfamily). S20B: BRIG comparative analysis of MOBQ12 plasmids using pCE10B as a reference. (PDF) [file pgen.1004766.s020.pdf]

Figure S21

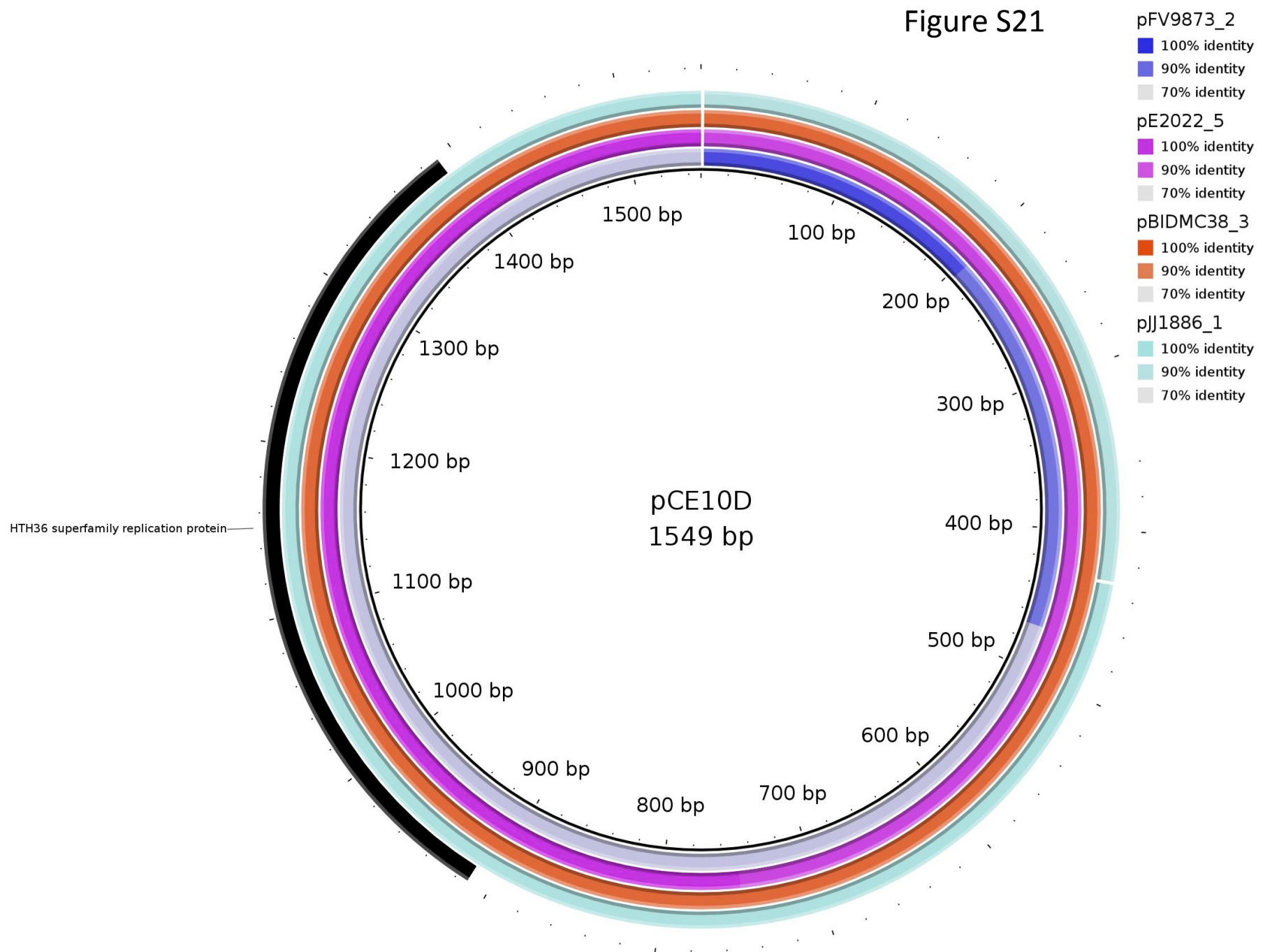

Supplement: S21 Fig — Comparative analysis of small no-MOB plasmids. BRIG comparative analysis of no-MOB plasmids using pCE10D as a reference. (PDF) [file pgen.1004766.s021.pdf]

Figure S22A

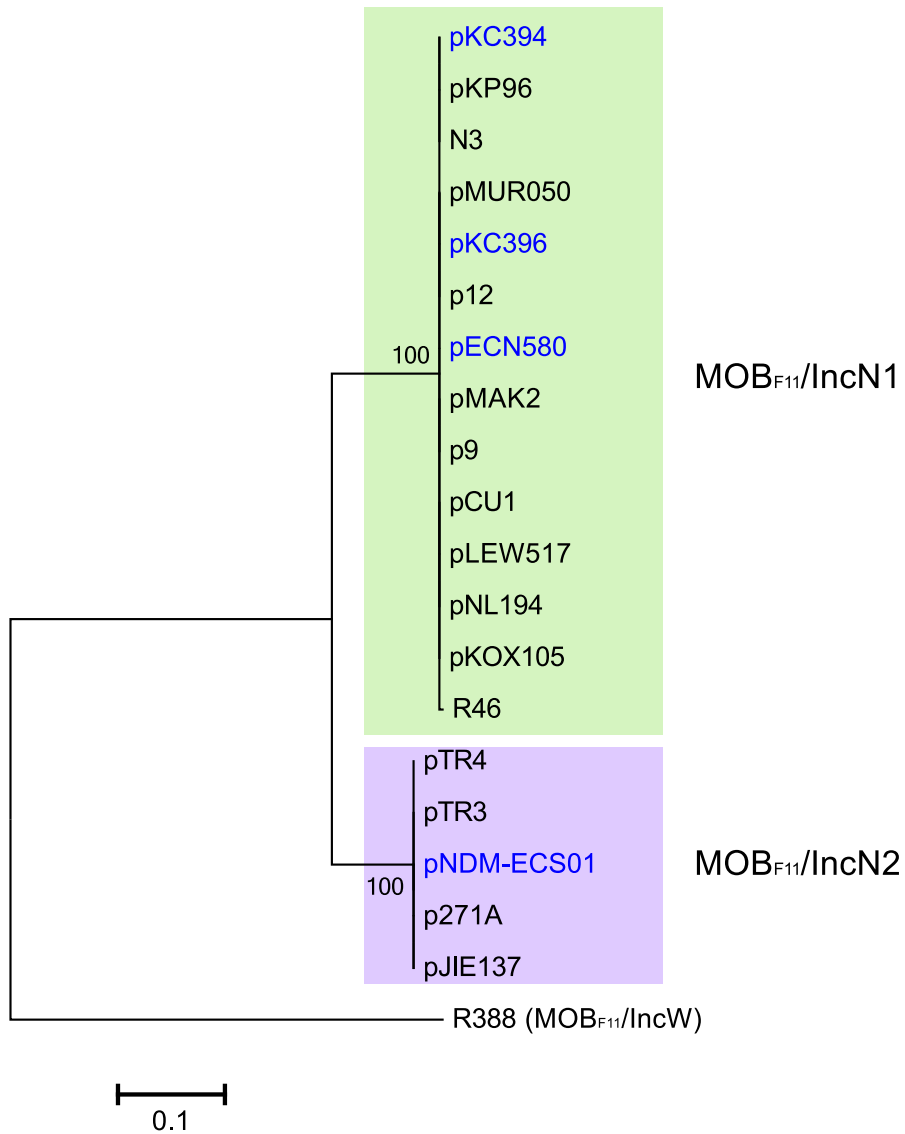

Figure S22B

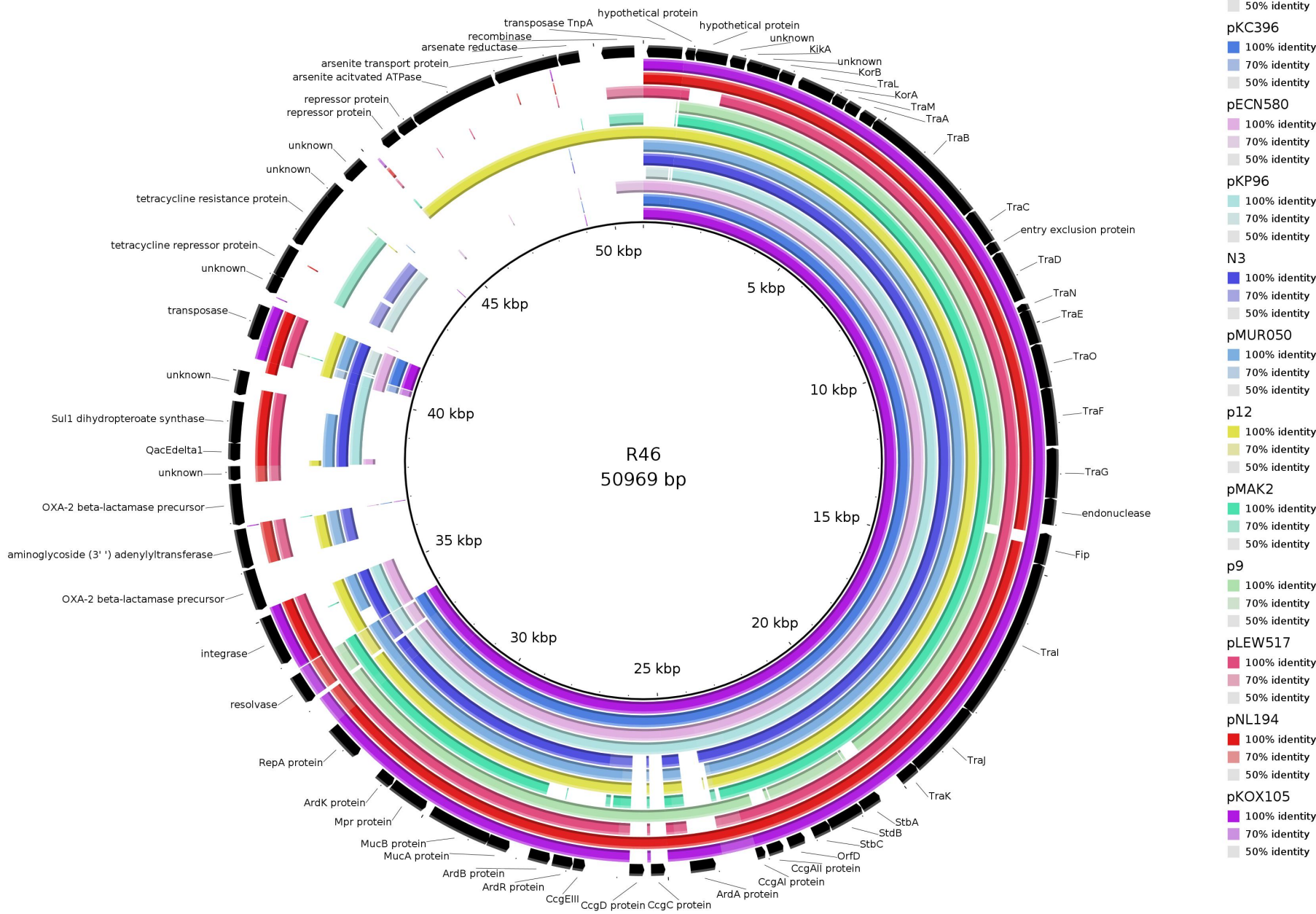

Figure S22C

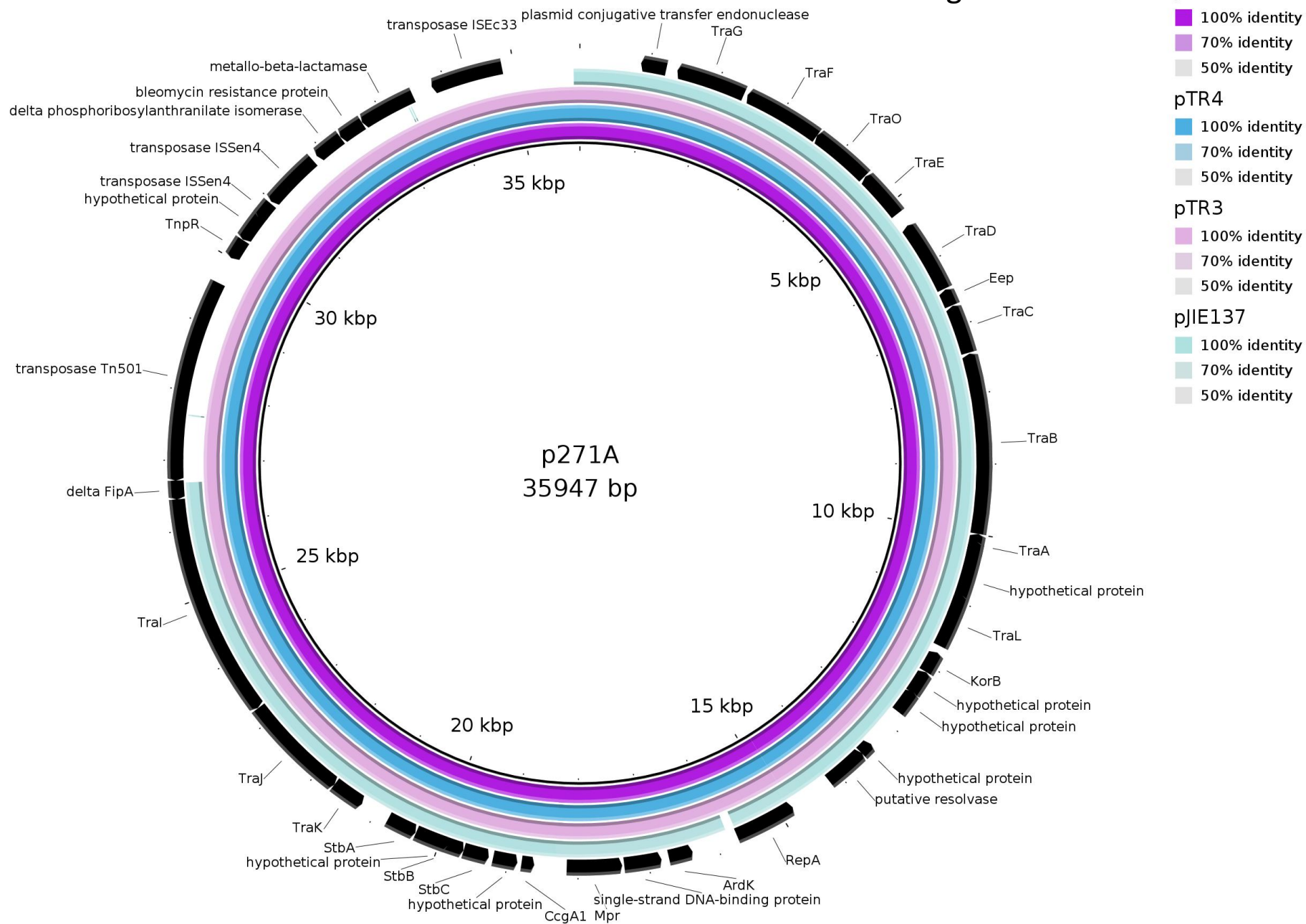

Supplement: S22 Fig — Comparative analysis of MOBF11/IncN plasmids. S22A: Phylogenetic tree of MOBF11 REL proteins, calculated as in Fig SF12B. The tree was rooted with R388 (MOBF11/IncW). IncN1 and IncN2 subgroups are indicated with different background colors. S22B: BRIG comparative analysis of IncN1 plasmids, using R46 as a reference. S22C: BRIG comparative analysis of IncN2 plasmids using p271A as a reference. (PDF) [file pgen.1004766.s022.pdf]

Figure S24

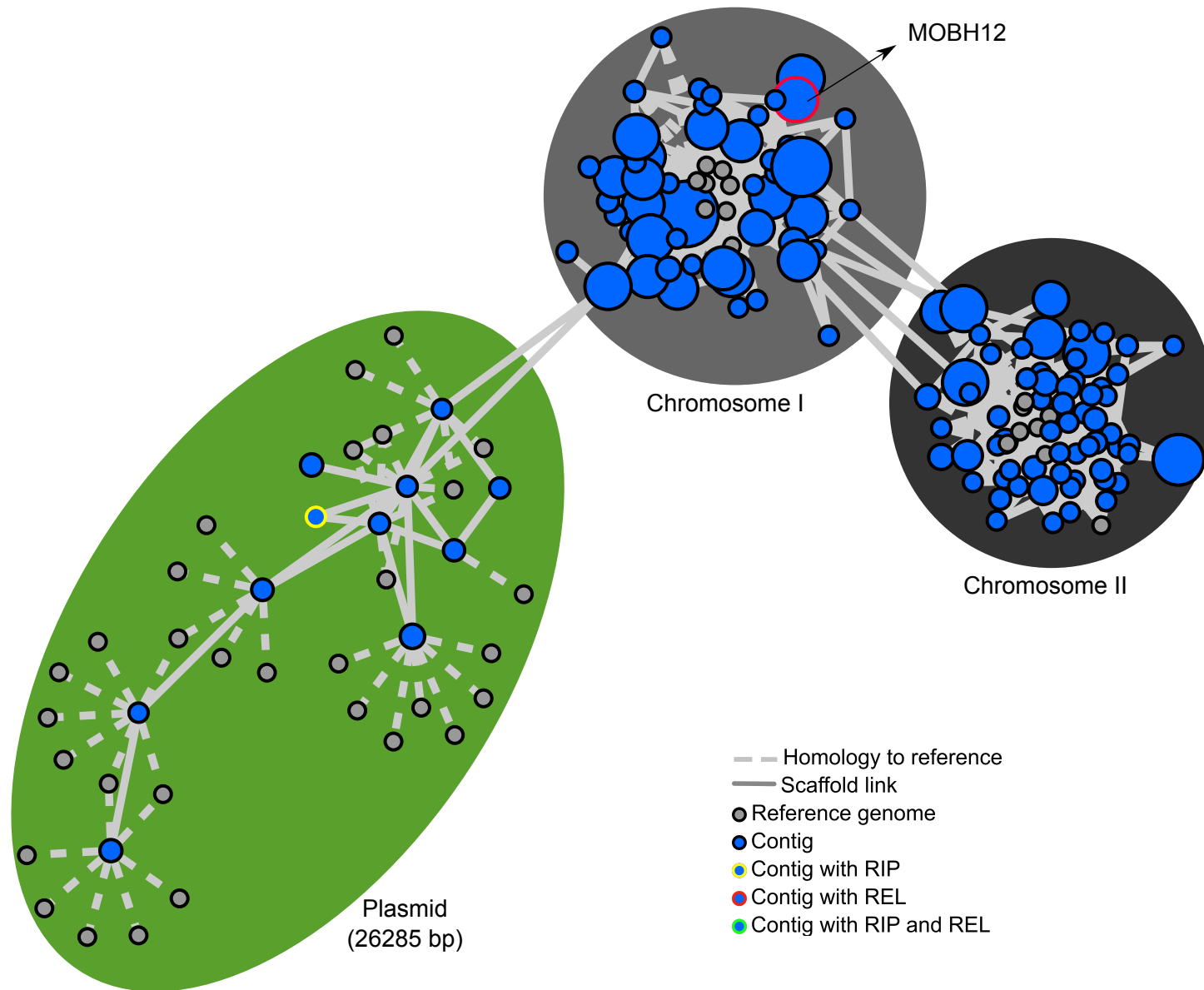

Supplement: S24 Fig — PLACNET reconstruction for a genome of Vibrio cholerae Pacini 1854 (ID: PRJEB2215). Assembly data: Number of libraries: 1; read length: 75 bp; number of contigs: 149; total bp: 4,022,287 bp; N50: 180089 and Kmer: 65. The two V. cholerae chromosomes were fully reconstructed. Chromosome I (2,992,142 bp in our study) was reported to be about 3 million bp and encodes most essential functions [95]. As shown in Fig S24, it harbors a MOBH12 relaxase, identical to that of the integrative and conjugative element (ICE) of the SXT/R391 family [96]. Chromosome II is smaller (1,034,286 bp in our study). Finally, a 26.3 kb plasmid containing a RIP protein (87% identity with E.coli pABU plasmid [97]) could be reconstructed. (PDF) [file pgen.1004766.s024.pdf]

STEP 1: 19 nodes 157-200 bp deleted

Original network

Pruned network

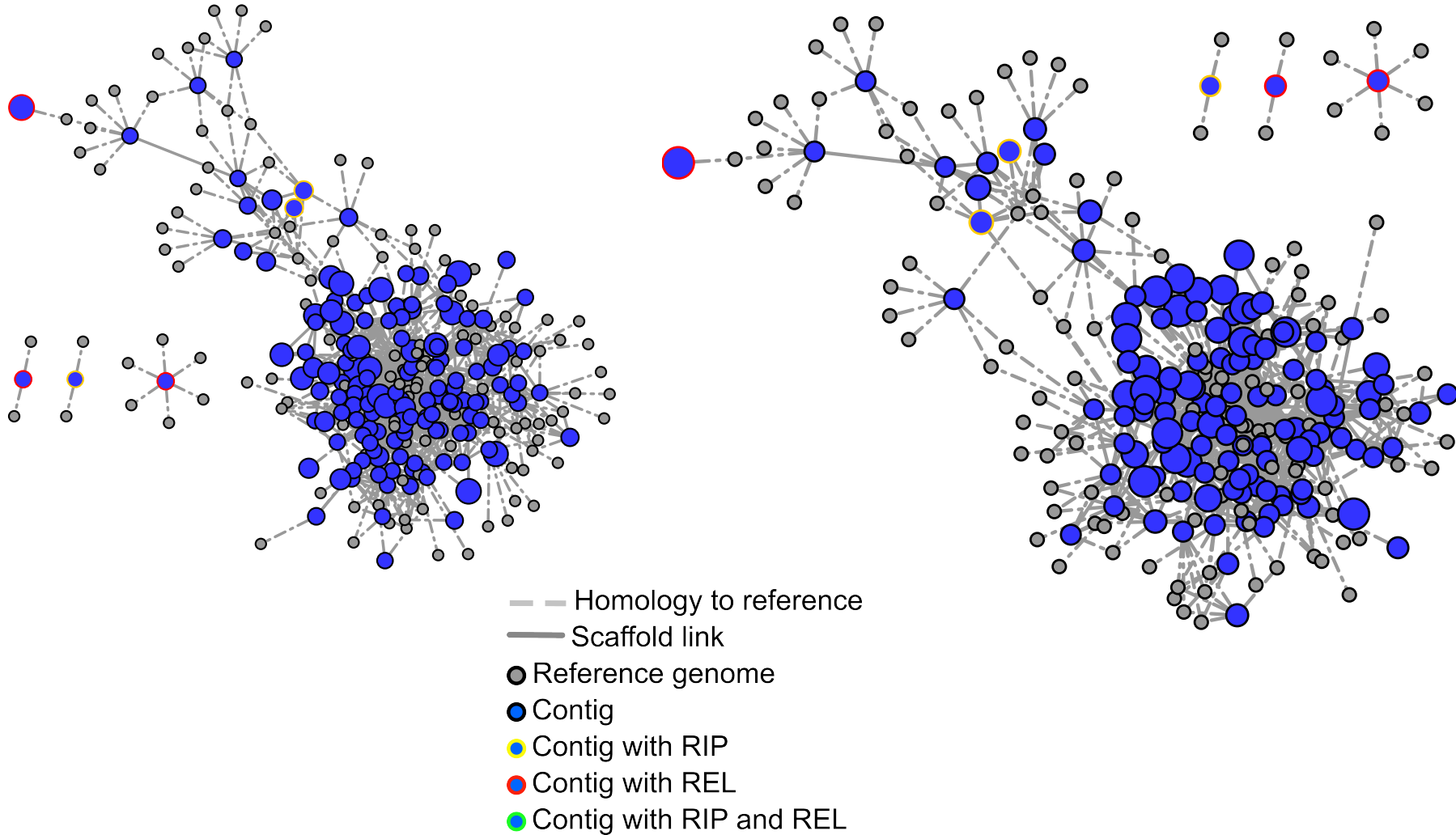

Supplement: S25 Fig — Cytoscape representation of the reconstructed E. coli JJ1886 genome. The network was constructed and codes used (in this and following figures) as explained in Fig. 6. The pruned network (Step 1) was obtained after deleting 19 contigs smaller than 200 bp. (PDF) [file pgen.1004766.s025.pdf]

Figure S29

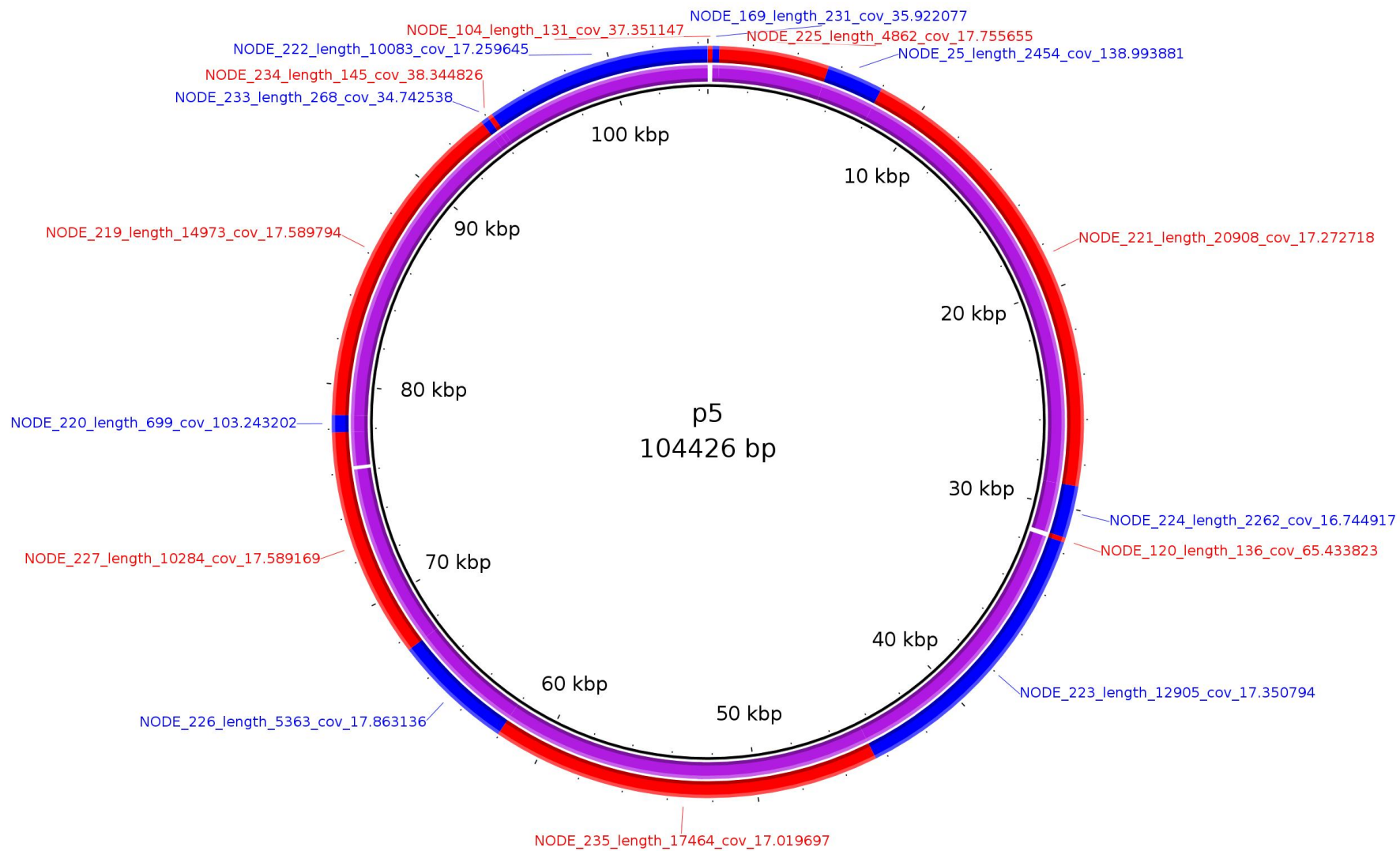

Supplement: S29 Fig — Inverse BRIG comparison of reconstructed plasmid p5 vs pJJ1886_5 reference plasmid. The reconstructed plasmid is placed here as the reference inner ring (thin black circle line) to which the reference plasmid (purple ring) and the reconstructed p5 contigs (outer blue and red ring) are compared. (PDF) [file pgen.1004766.s029.pdf]

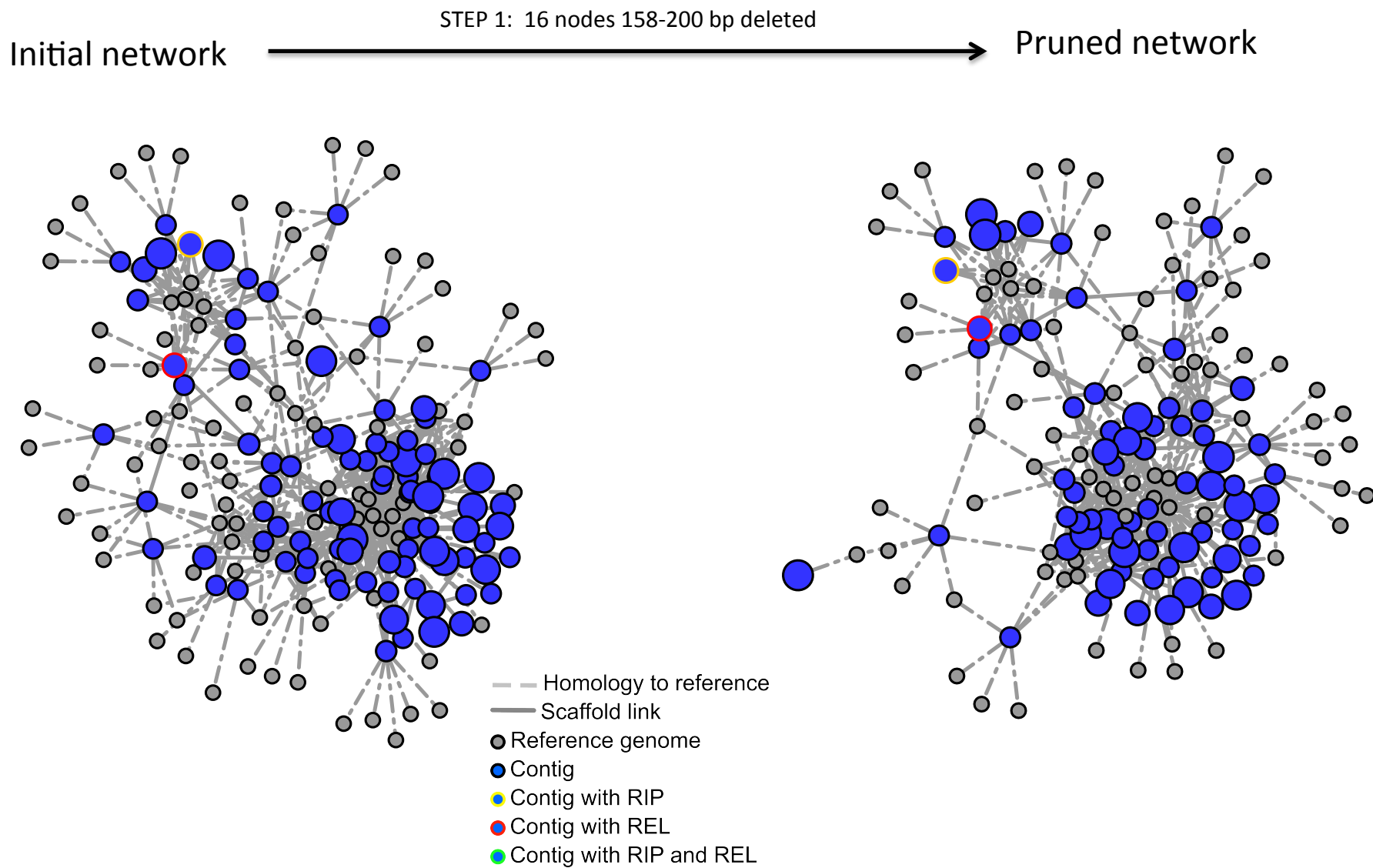

Supplement: S30 Fig — Cytoscape representation of the reconstructed E. coli SE15 genome. The network was constructed and codes used as explained in Fig. 6. The pruned network (Step 1) was obtained after deleting 16 contigs smaller than 200 bp. (PDF) [file pgen.1004766.s030.pdf]
