# Supplementary material for: Plasmid Flux in Escherichia coli ST131 Sublineages, Analyzed by Plasmid Constellation Network (PLACNET), a New Method for Plasmid Reconstruction from Whole Genome Sequences
Source: PLoS Genet. 2014 Dec 18;10(12):e1004766. doi: 10.1371/journal.pgen.1004766 (PMC4270462; doi:10.1371/journal.pgen.1004766)
Supplement: S13 Fig — BRIG comparative analysis of MOBP12/IncI-complex. S13A: The IncI1 plasmid pEK204 is used as inner ring in the BRIG analysis. S13B: Plasmid pCT [62] was used as reference. (PDF) [file pgen.1004766.s013.pdf]

Figure S13A

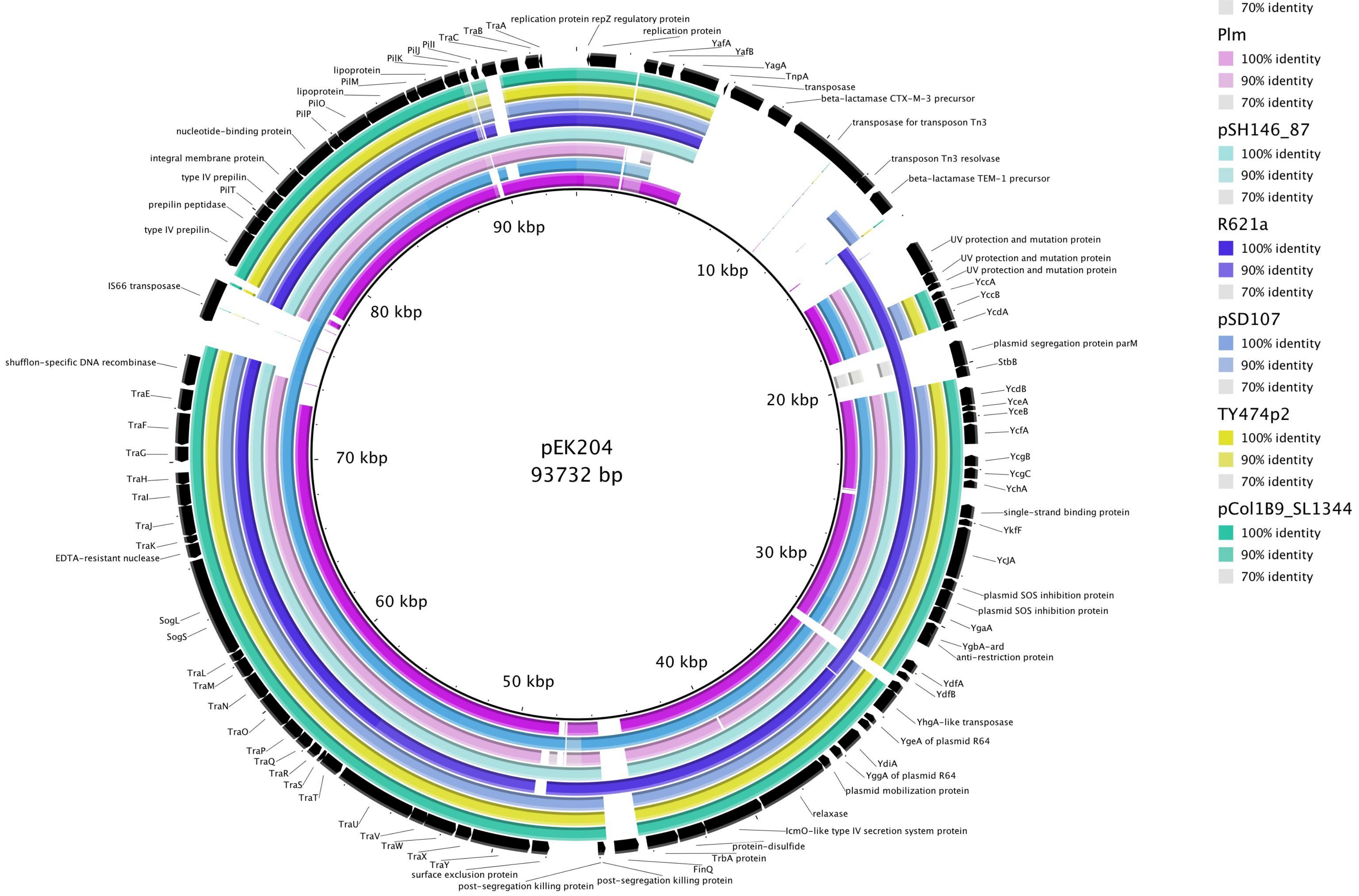
pEK204
93732 bp
10 kbp
20 kbp
30 kbp
40 kbp
50 kbp
60 kbp
70 kbp
80 kbp
90 kbp
replication protein repZ regulatory protein
replication protein
YafA
YafB
YagA
TnpA
transposase
beta-lactamase CTX-M-3 precursor
transposase for transposon Tn3
transposon Tn3 resolvase
beta-lactamase TEM-1 precursor
UV protection and mutation protein
UV protection and mutation protein
UV protection and mutation protein
YccA
YccB
YcdA
plasmid segregation protein parM
StbB
YcdB
YceA
YceB
YcfA
YcgB
YcgC
YchA
single-strand binding protein
YkfF
YcJA
plasmid SOS inhibition protein
plasmid SOS inhibition protein
YgaA
YgbA-ard
anti-restriction protein
YdfA
YdfB
YhgA-like transposase
YgeA of plasmid R64
YdiA
YggA of plasmid R64
plasmid mobilization protein
relaxase
IcmO-like type IV secretion system protein
protein-disulfide
TrbA protein
FinQ
post-segregation killing protein
post-segregation killing protein
surface exclusion protein
TraY
TraX
TraW
TraV
TraU
TraT
TraS
TraR
TraQ
TraP
TraO
TraN
TraM
TraL
SogS
SogL
EDTA-resistant nuclease
TraK
TraJ
TraI
TraH
TraG
TraF
TraE
shufflon-specific DNA recombinase
IS66 transposase
type IV prepilin
prepilin peptidase
PilT
type IV prepilin
integral membrane protein
nucleotide-binding protein
PilP
PilO
lipoprotein
PilM
lipoprotein
PilK
PilJ
PilI
TraC
TraB
TraA
pSE11-1
100% identity
90% identity
70% identity
pPWD4-103
100% identity
90% identity
70% identity
Plm
100% identity
90% identity
70% identity
pSH146_87
100% identity
90% identity
70% identity
R621a
100% identity
90% identity
70% identity
pSD107
100% identity
90% identity
70% identity
TY474p2
100% identity
90% identity
70% identity
pCol1B9_SL1344
100% identity
90% identity
70% identity

Figure S13B

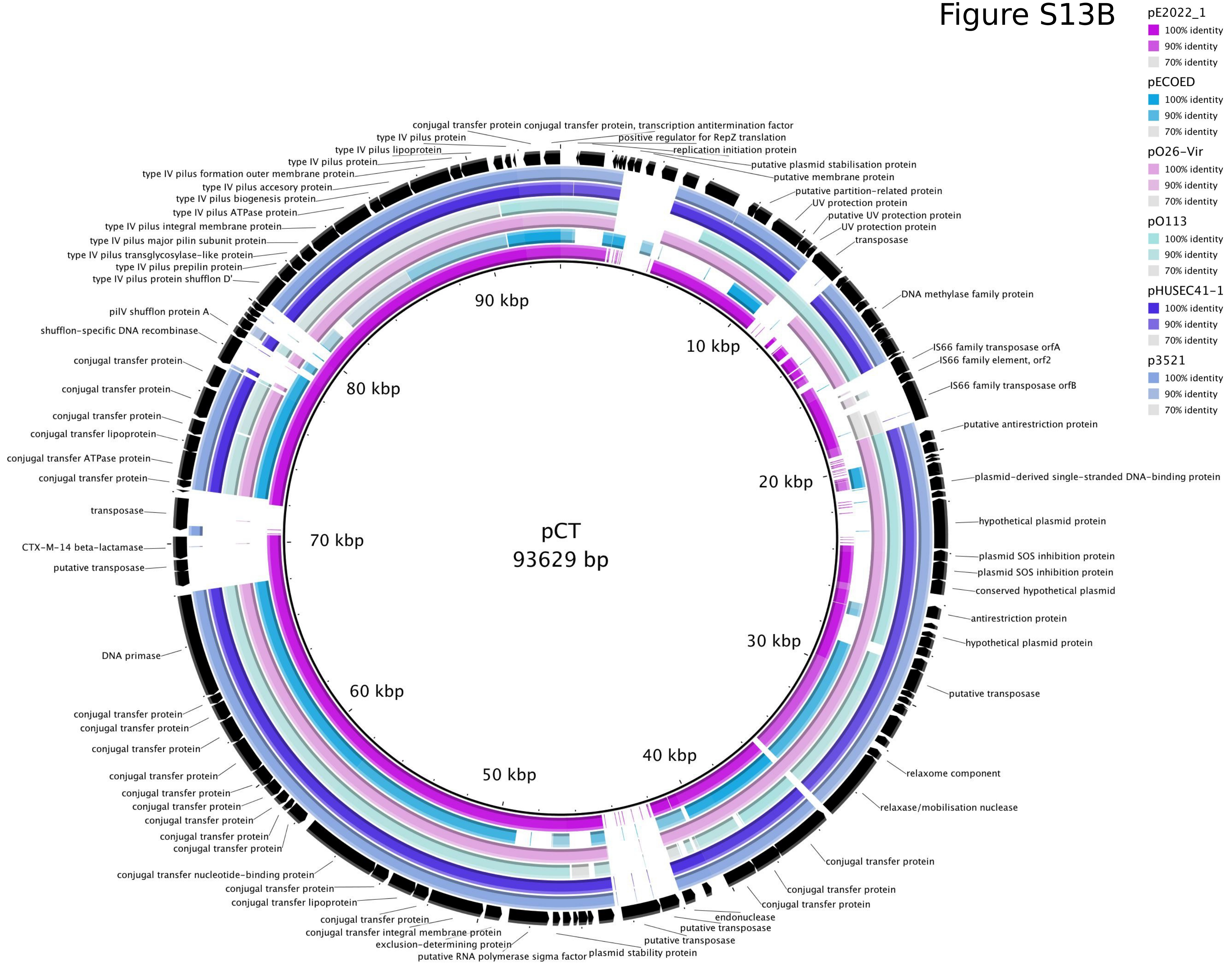
pE2022_1
100% identity
90% identity
70% identity
pECOED
100% identity
90% identity
70% identity
pO26-Vir
100% identity
90% identity
70% identity
pO113
100% identity
90% identity
70% identity
pHUSEC41-1
100% identity
90% identity
70% identity
p3521
100% identity
90% identity
70% identity
pCT
93629 bp
10 kbp
20 kbp
30 kbp
40 kbp
50 kbp
60 kbp
70 kbp
80 kbp
90 kbp
conjugal transfer protein
conjugal transfer protein, transcription antitermination factor
type IV pilus protein
positive regulator for RepZ translation
type IV pilus lipoprotein
replication initiation protein
type IV pilus protein
putative plasmid stabilisation protein
type IV pilus formation outer membrane protein
putative membrane protein
type IV pilus accesory protein
putative partition-related protein
type IV pilus biogenesis protein
UV protection protein
type IV pilus ATPase protein
putative UV protection protein
type IV pilus integral membrane protein
UV protection protein
type IV pilus major pilin subunit protein
transposase
type IV pilus transglycosylase-like protein
type IV pilus prepilin protein
type IV pilus protein shufflon D'
DNA methylase family protein
pilV shufflon protein A
shufflon-specific DNA recombinase
IS66 family transposase orfA
conjugal transfer protein
IS66 family element, orf2
IS66 family transposase orfB
conjugal transfer protein
conjugal transfer protein
putative antirestriction protein
conjugal transfer lipoprotein
conjugal transfer ATPase protein
plasmid-derived single-stranded DNA-binding protein
conjugal transfer protein
transposase
hypothetical plasmid protein
CTX-M-14 beta-lactamase
plasmid SOS inhibition protein
putative transposase
plasmid SOS inhibition protein
conserved hypothetical plasmid
antirestriction protein
DNA primase
hypothetical plasmid protein
putative transposase
conjugal transfer protein
conjugal transfer protein
conjugal transfer protein
relaxome component
conjugal transfer protein
relaxase/mobilisation nuclease
conjugal transfer protein
conjugal transfer protein
conjugal transfer protein
conjugal transfer protein
conjugal transfer protein
conjugal transfer protein
conjugal transfer protein
conjugal transfer nucleotide-binding protein
conjugal transfer protein
conjugal transfer protein
endonuclease
conjugal transfer lipoprotein
putative transposase
conjugal transfer protein
putative transposase
conjugal transfer integral membrane protein
exclusion-determining protein
plasmid stability protein
putative RNA polymerase sigma factor
