# Supplementary material for: Plasmid Flux in Escherichia coli ST131 Sublineages, Analyzed by Plasmid Constellation Network (PLACNET), a New Method for Plasmid Reconstruction from Whole Genome Sequences
Source: PLoS Genet. 2014 Dec 18;10(12):e1004766. doi: 10.1371/journal.pgen.1004766 (PMC4270462; doi:10.1371/journal.pgen.1004766)
Supplement: S23 Fig — PLACNET reconstruction of the genome of Staphylococcus aureus strain 118 (ST772) (ID: PRJNA82607). Assembly data: Number of libraries: 1; read length: 75 bp; number of contigs: 73; total bp: 2,798,022 bp; N50: 224673 and Kmer: 73. One 12,819 bp plasmid was identified and reconstructed. No REL or RIP proteins were detected. (PDF) [file pgen.1004766.s023.pdf]

Figure S23

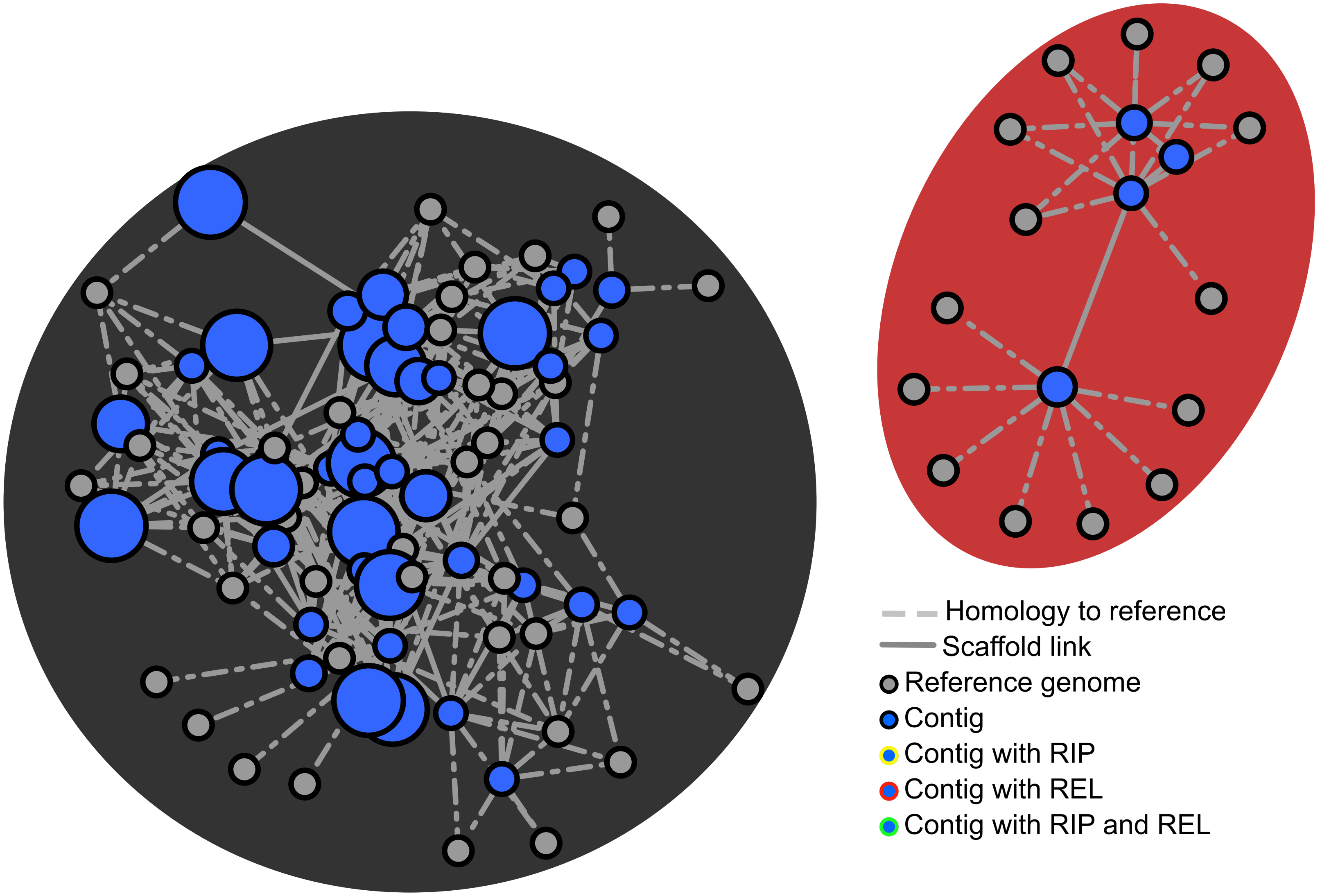
Homology to reference
Scaffold link
Reference genome
Contig
Contig with RIP
Contig with REL
Contig with RIP and REL
