# Supplementary material for: Plasmid Flux in Escherichia coli ST131 Sublineages, Analyzed by Plasmid Constellation Network (PLACNET), a New Method for Plasmid Reconstruction from Whole Genome Sequences
Source: PLoS Genet. 2014 Dec 18;10(12):e1004766. doi: 10.1371/journal.pgen.1004766 (PMC4270462; doi:10.1371/journal.pgen.1004766)

Figure S26

STEP 2: Initial plasmid analysis

Node (chr. 17x aprox.)	Blastn/Blastx	Copy number (cov. based)	Decision
NODE_215_length_1552_cov_360.108551	RepA	21	p1
NODE_216_length_5167_cov_176.524261	RepA , Mobilization protein (MOBQ12)	10	p2
NODE_218_length_55955_cov_105.076561	Conjugal transfer proteins (TraI: MOBP11), plasmid stabilization proteins (parAB), RepA.	6	p4
NODE_217_length_5601_cov_175.993118	RepA, Mobilization protein (MOBP5)	10	p3

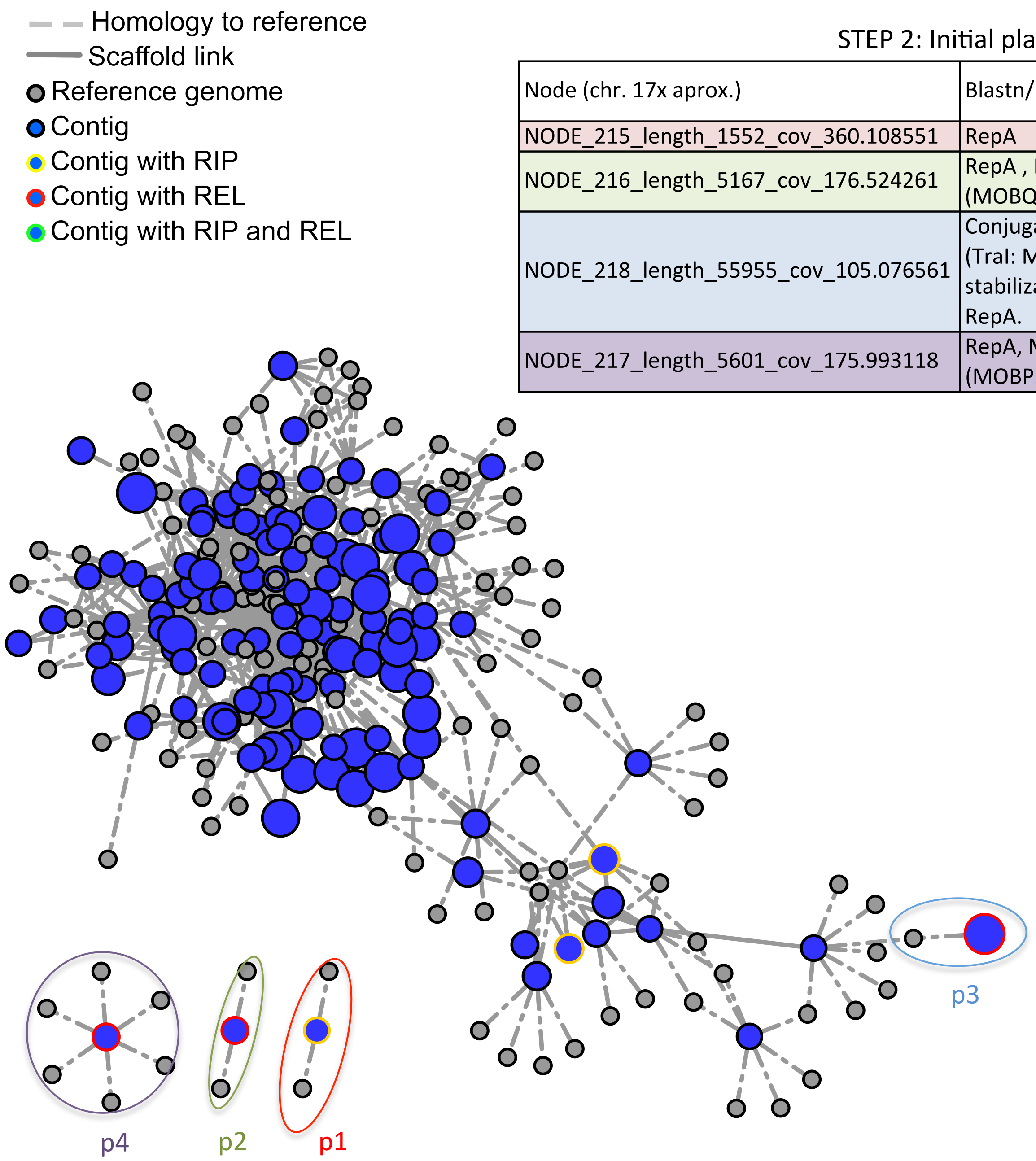

Supplement: S26 Fig — Definition (Step 2) of E. coli JJ1886 plasmids p1 to p4. These plasmids contain a RIP and/or REL protein and appeared as single contigs. The inset Table shows some properties of relevant nodes. The nodes are represented in the network surrounded by circles of the same color than the background color in the Table. (PDF) [file pgen.1004766.s026.pdf]
