# Supplementary material for: Plasmid Flux in Escherichia coli ST131 Sublineages, Analyzed by Plasmid Constellation Network (PLACNET), a New Method for Plasmid Reconstruction from Whole Genome Sequences
Source: PLoS Genet. 2014 Dec 18;10(12):e1004766. doi: 10.1371/journal.pgen.1004766 (PMC4270462; doi:10.1371/journal.pgen.1004766)
Supplement: S27 Fig — Resolution of hubs and definition of plasmid p5 (Step 3). Hub nodes that were duplicated are shown in the inset Table and indicated by red arrows in the Cytoscape network. After hub duplication, the IncF plasmid p5 is shown to contain the 12 contigs surrounded by a red circle. (PDF) [file pgen.1004766.s027.pdf]

Figure S27

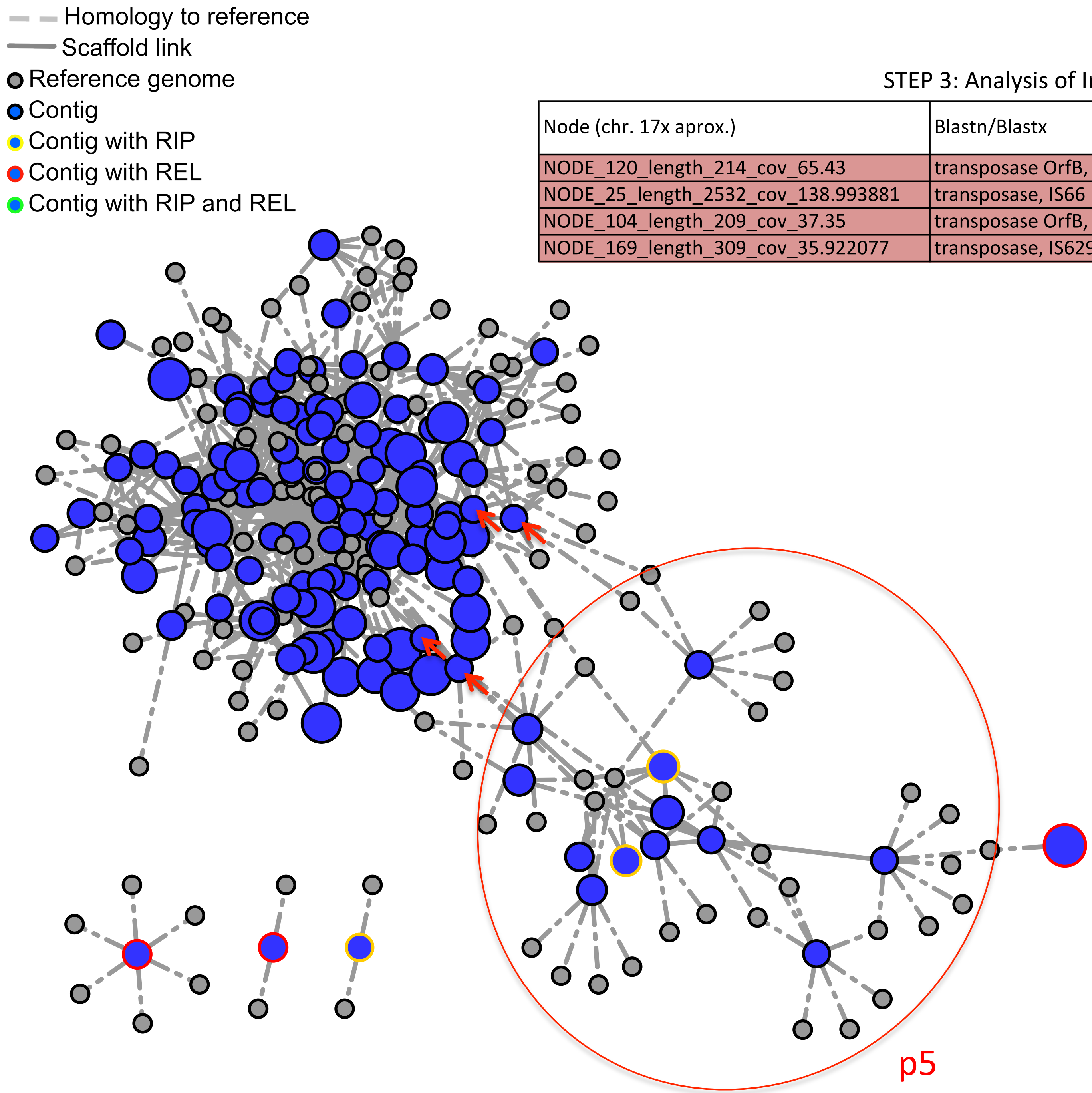

STEP 3: Analysis of IncF plasmid

Node (chr. 17x aprox.)	Blastn/Blastx	Copy number (cov. based)	Decision
NODE_120_length_214_cov_65.43	transposase OrfB, IS911	4	Hub*
NODE_25_length_2532_cov_138.993881	transposase, IS66	8	Hub*
NODE_104_length_209_cov_37.35	transposase OrfB, IS911	2	Hub*
NODE_169_length_309_cov_35.922077	transposase, IS629	2	Hub*
