# Supplementary material for: Plasmid Flux in Escherichia coli ST131 Sublineages, Analyzed by Plasmid Constellation Network (PLACNET), a New Method for Plasmid Reconstruction from Whole Genome Sequences
Source: PLoS Genet. 2014 Dec 18;10(12):e1004766. doi: 10.1371/journal.pgen.1004766 (PMC4270462; doi:10.1371/journal.pgen.1004766)

# Figure S31

STEP 2: Initial plasmid and hub analysis

| Node (chr. 17x aprox.) | Blastn/Blastx | Copy number (cov. based) | Decision |
|---|---|---|---|
| NODE_18_length_88778_cov_17.254745 | putative DNA helicase, conserved hypothetical protein | 1 | Chr |
| NODE_39_length_339_cov_36.088123 | transposase IS1 | 2 | Chr |
| NODE_23_length_708_cov_35.866665 | MATE family multidrug exporter | 2 | Chr |
| NODE_116_length_939_cov_35.117306 | transposase IS100 | 2 | Hub* |
| NODE_5_length_2581_cov_103.488617 | putative phage tail protein, transposase | 6 | Hub* |
| NODE_145_length_559_cov_37.085239 | hypothetical protein, reverse transcriptase | 2 | Hub* |

Hub* : duplicated

Chr : assigned to chromosome

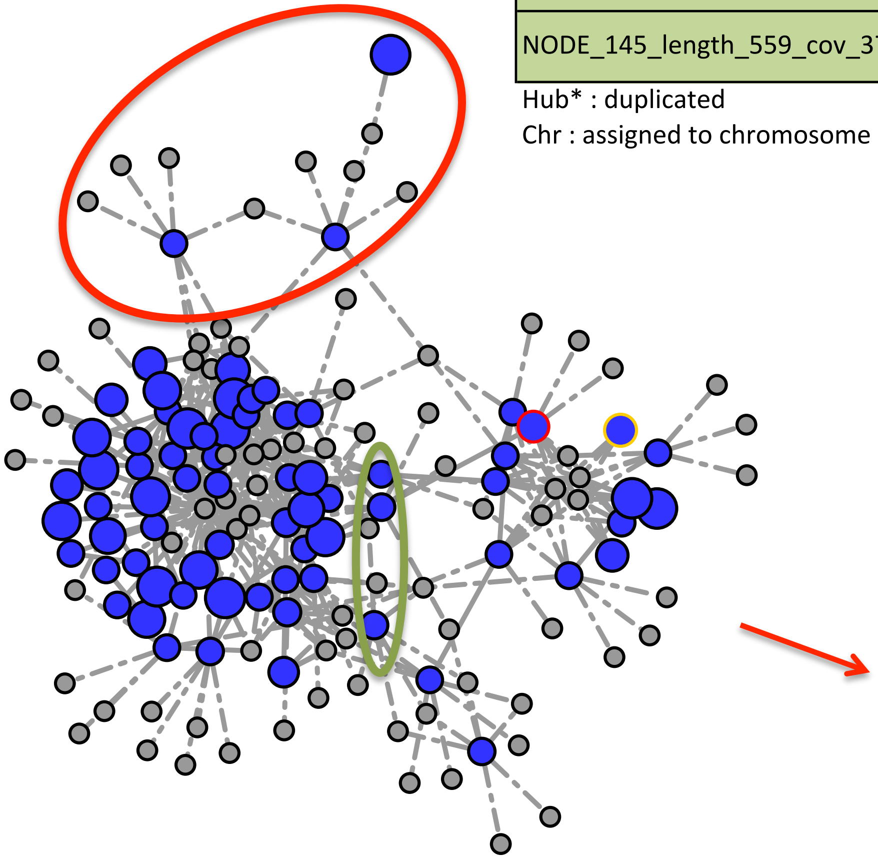

STEP 3: Hub duplication and definition of p1 plasmid

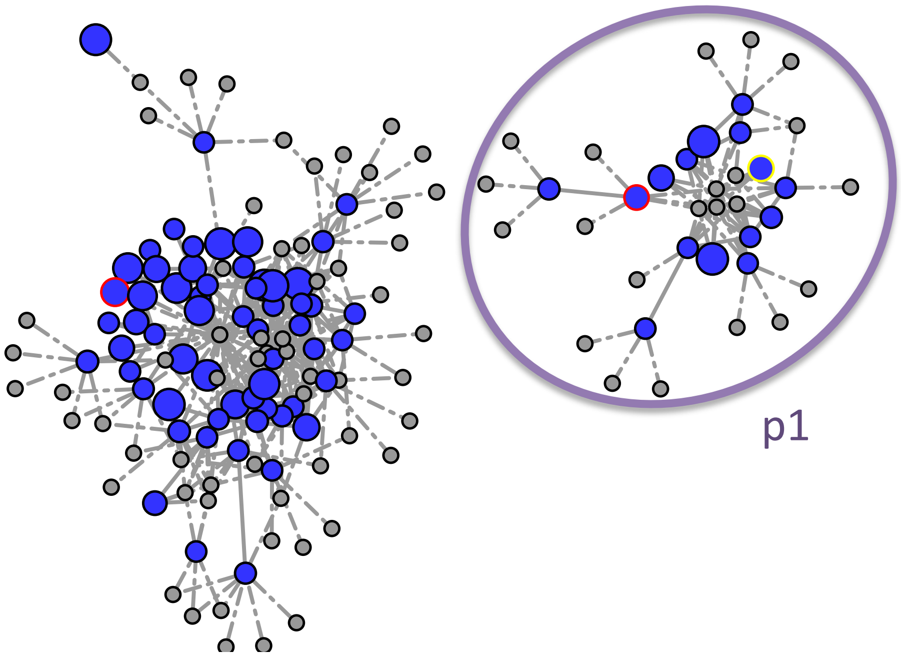

Supplement: S31 Fig — Plasmid definition (PLACNET steps 2 and 3) of E. coli SE15 genome. Three particular nodes in the pruned network (surrounded by the red circle) were scrutinized due to their loose connection to the chromosome. As shown in the inset Table (red background files), a blastx comparison indicates they correspond to “typical” E. coli chromosomal segments, so were finally assigned to the chromosome. Three other nodes (surrounded by a green circle in the left Cytoscape representation) corresponded to hubs (green background in the inset) and were thus duplicated. The reconstructed plasmid (p1) in the final network is surrounded by a purple ring. (PDF) [file pgen.1004766.s031.pdf]
