# Supplementary material for: Plasmid Flux in Escherichia coli ST131 Sublineages, Analyzed by Plasmid Constellation Network (PLACNET), a New Method for Plasmid Reconstruction from Whole Genome Sequences
Source: PLoS Genet. 2014 Dec 18;10(12):e1004766. doi: 10.1371/journal.pgen.1004766 (PMC4270462; doi:10.1371/journal.pgen.1004766)
Supplement: S32 Fig — BRIG comparison of SE15 E. coli genome reconstructed IncF plasmid (p1) with the reference plasmid pECSF1. The reference plasmid (inner ring) is compared to the IncF reconstructed plasmid (purple ring). Outer black and white ring sectors represent pECSF1 gene annotations. Three regions (5,709 bp in total) were missing from the p1 reconstruction. (PDF) [file pgen.1004766.s032.pdf]

Figure S32

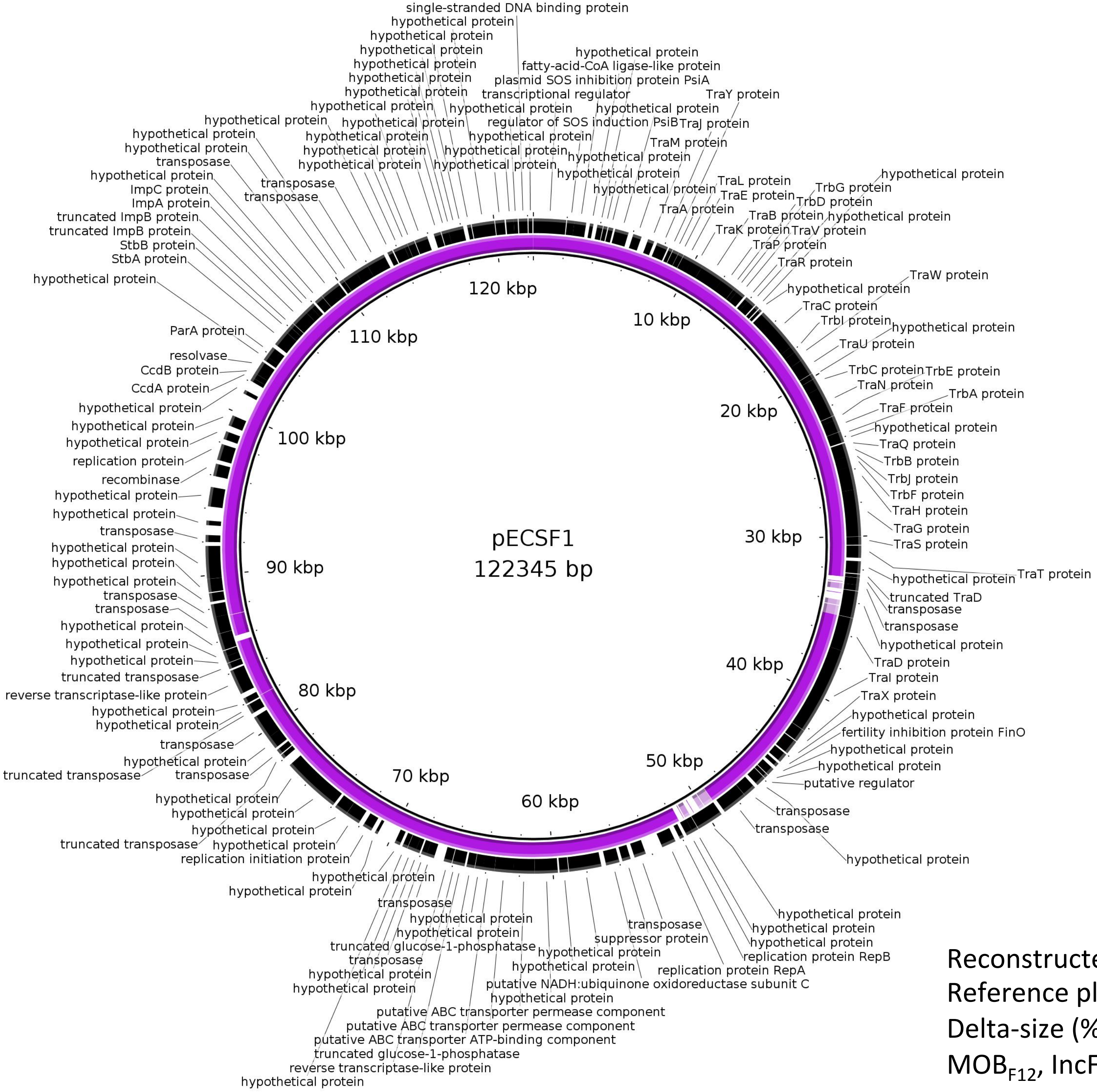

Reconstructed plasmid (p1) : 116,636 bp

Reference plasmid pECSF1 : 122,345 bp

Delta-size (% error): 5,709 bp (5 %)

MOB_{F12}, IncF plasmid
