# Supplementary material for: Plasmid Flux in Escherichia coli ST131 Sublineages, Analyzed by Plasmid Constellation Network (PLACNET), a New Method for Plasmid Reconstruction from Whole Genome Sequences
Source: PLoS Genet. 2014 Dec 18;10(12):e1004766. doi: 10.1371/journal.pgen.1004766 (PMC4270462; doi:10.1371/journal.pgen.1004766)
Supplement: S33 Fig — Cytoscape representation of the reconstructed genome of E. coli strain MG1655 containing plasmids pEC958 and R46. The network was constructed and codes used as explained in Fig. 6. The pruned network was obtained after deleting 25 contigs smaller than 200 bp and duplicating 2 hubs (surrounded by a red circle). Plasmid p1 is the reconstructed R46 while p2 is the reconstructed pEC958. Nodes surrounded by a blued circle, and described by the blue background files in the inset Table, could not be assigned. See text for further details. (PDF) [file pgen.1004766.s033.pdf]

Figure S33

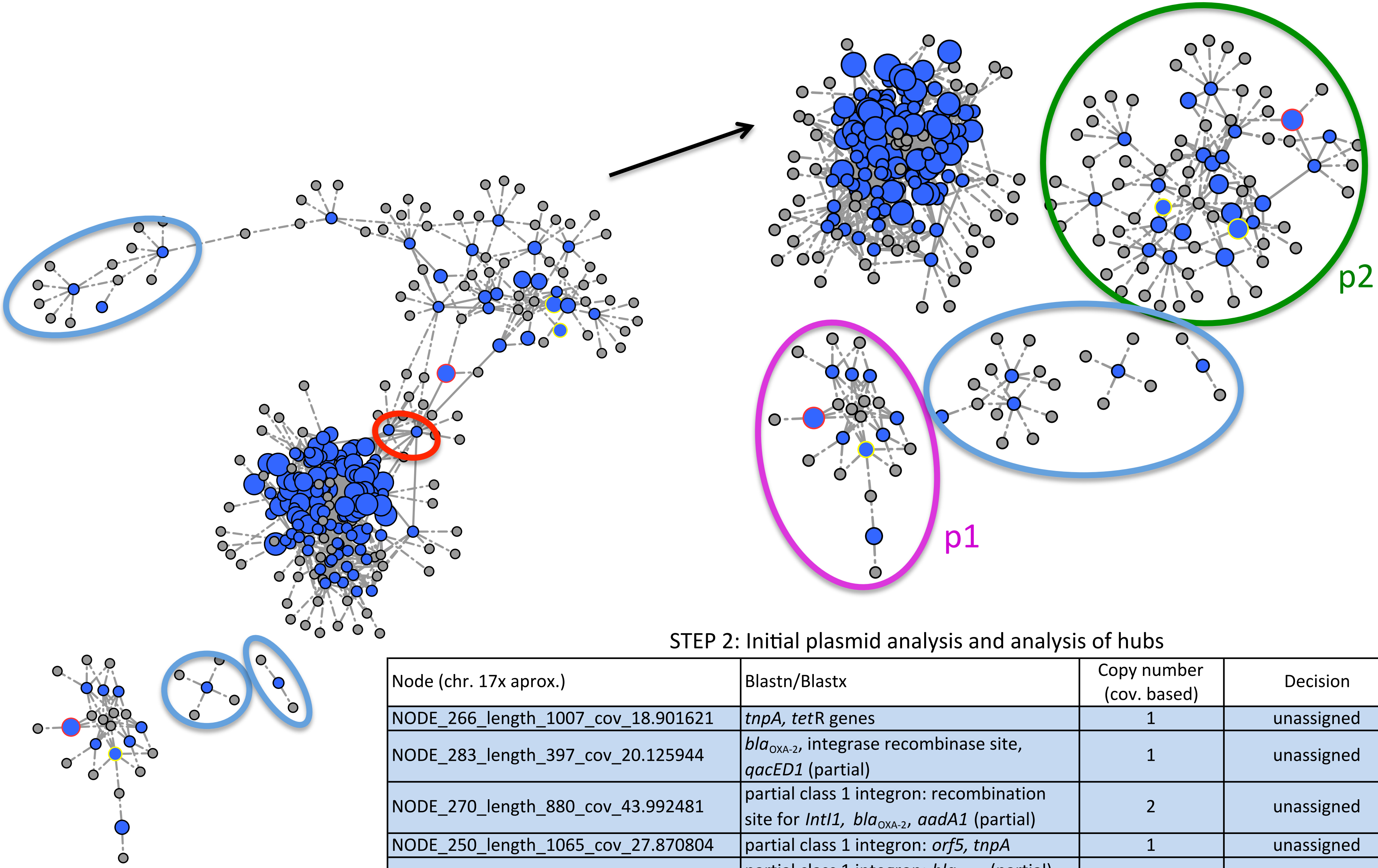

STEP 2: Initial plasmid analysis and analysis of hubs

Node (chr. 17x aprox.)	Blastn/Blastx	Copy number (cov. based)	Decision
NODE_266_length_1007_cov_18.901621	*tnpA, tet*R genes	1	unassigned
NODE_283_length_397_cov_20.125944	bla_{OXA-2}, integrase recombinase site, *qacED1* (partial)	1	unassigned
NODE_270_length_880_cov_43.992481	partial class 1 integron: recombination site for *IntI1*, bla_{OXA-2}, *aadA1* (partial)	2	unassigned
NODE_250_length_1065_cov_27.870804	partial class 1 integron: *orf5, tnpA*	1	unassigned
NODE_276_length_1016_cov_23.614561	partial class 1 integron: bla_{OXA-2} (partial), *aadA1*, bla_{OXA-2}(partial)	1	unassigned
NODE_144_length_247_cov_202.624237	TnpA_IS1	9	Hub*
NODE_100_length_259_cov_204.949158	TnpA_IS1	9	Hub*

Hub* : duplicated
