# Supplementary material for: Plasmid Flux in Escherichia coli ST131 Sublineages, Analyzed by Plasmid Constellation Network (PLACNET), a New Method for Plasmid Reconstruction from Whole Genome Sequences
Source: PLoS Genet. 2014 Dec 18;10(12):e1004766. doi: 10.1371/journal.pgen.1004766 (PMC4270462; doi:10.1371/journal.pgen.1004766)
Supplement: S34 Fig — Final Cytoscape representation of reconstructed H0407 E. coli genome. The network was constructed as explained in Fig. 6. The pruned network was obtained after deleting 44 contigs smaller than 200 bp. Plasmids p1 and p2, represented by single contigs, are surrounded by red and blue circles, respectively. A single contig (surrounded in an intense blue circle) remained isolated from other genetic units. Nevertheless, blastx analysis demonstrate it correspond to chromosomal background, as shown in the inset Table. Nodes described by the grey background files in the inset Table correspond to one node assigned to the chromosome, plus seven hubs, which were duplicated. The green circle surrounds 52 contigs, adding 119,735 bp, which represent plasmids p3 and p4, the two IncF plasmids that PLACNET was not able to resolve (see text for further details). The red arrow indicates a node containing REL, RIP and backbone genes in common to both IncF plasmids. (PDF) [file pgen.1004766.s034.pdf]

Figure S34

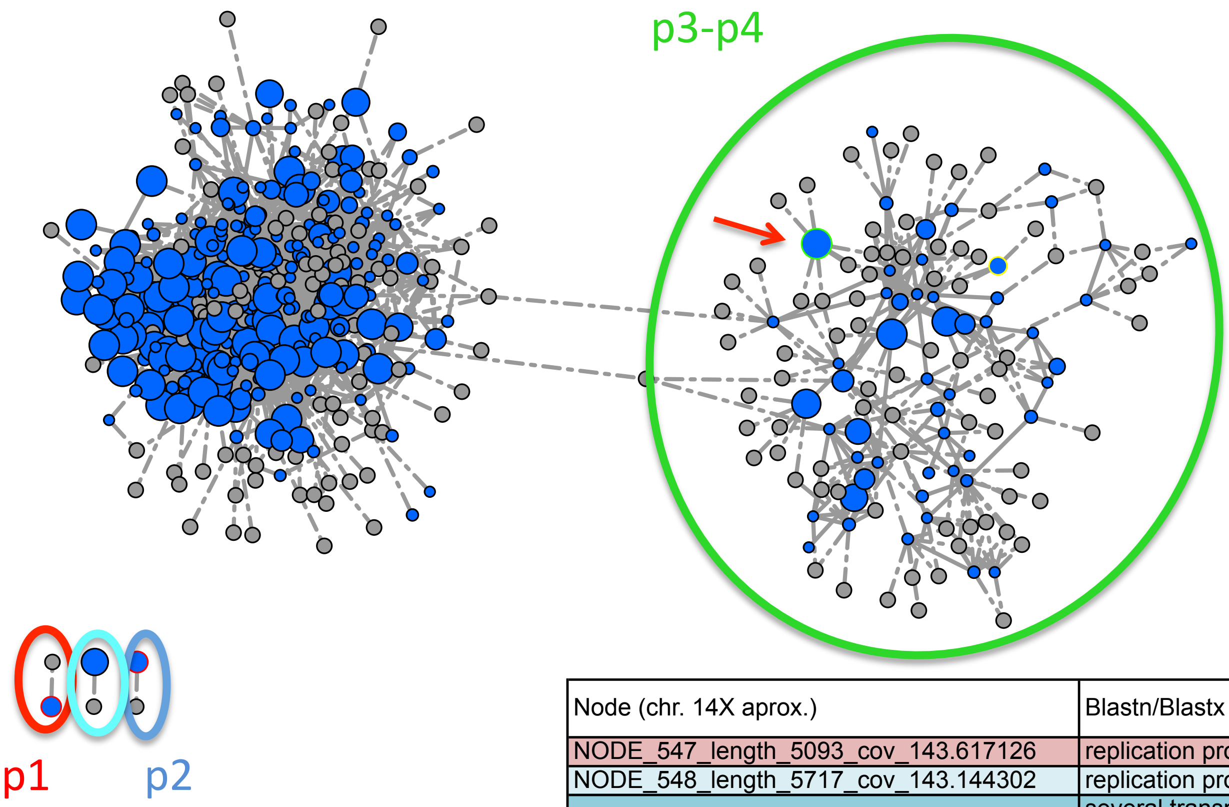

Node (chr. 14X aprox.)	Blastn/Blastx	Copy number (cov. Based)	Decision
NODE_547_length_5093_cov_143.617126	replication protein, mob genes	10	p1
NODE_548_length_5717_cov_143.144302	replication protein, mob genes	10	p2
NODE_250_length_8692_cov_13.472733	several transposases, hypothetical proteins, copper binding protein, adhesin tibA and the tibA precursor.	1	Chr
NODE_229_length_9069_cov_12.583967	putative terminator protein	1	Chr
NODE_60_length_1232_cov_111.675323	IS1414 transposase	8	Hub*
NODE_188_length_125_cov_199.679993	hypothetical transposase	14	Hub*
NODE_123_length_841_cov_218.697983	putative transposase	16	Hub*
NODE_69_length_552_cov_228.652176	putative transposase	16	Hub*
NODE_390_length_428_cov_54.207943	putative transposase	4	Hub*
NODE_266_length_508_cov_27.196850	putative transposase	2	Hub*
NODE_586_length_198_cov_86.535355	IS629 transposase	6	Hub*
NODE_554_length_15758_cov_14.111690	putative toxin-antitoxin system, hypothetical protein, replication protein, conserved hypothetical protein, finO, traX, traI, traD	1	p3-p4

Hub* : duplicated
Chr: assigned to chromosome
