# Supplementary material for: Plasmid Flux in Escherichia coli ST131 Sublineages, Analyzed by Plasmid Constellation Network (PLACNET), a New Method for Plasmid Reconstruction from Whole Genome Sequences
Source: PLoS Genet. 2014 Dec 18;10(12):e1004766. doi: 10.1371/journal.pgen.1004766 (PMC4270462; doi:10.1371/journal.pgen.1004766)
Supplement: S2 Table — Ten E. coli genomes analyzed as examples of PLACNET performance. (PDF) [file pgen.1004766.s037.pdf]

Table S2. Ten *E. coli* genomes analyzed as examples of PLACNET performance [1]

Genome[2]	Plasmid	Accession no.	Estimated copy number	Coverage	Nº contigs	Total bp	N50 (bp)	Kmer	Longest contig (bp)
**JJ1886 (ST131, ExPEC)**	pJJ1886_1	NC_022661	20	2000x	150	5,211,142	237,023	79	710,527
	pJJ1886_2	NC_022649	10	1000x					
	pJJ1886_3	NC_022662	10	1000x					
	pJJ1886_4	NC_022650	6	600x					
	pJJ1886_5	NC_022651	1	100x					
	chromosome	NC_022648	1	100x					
**SE15 (ST131, ExPEC)**	pECSF1	NC_013655	1	100x	91	4,785,093	367,892	79	659,604
	chromosome	NC_013654	1	100x					
**UTI89 (ExPEC)**	pUTI89	NC_007941	1	100x	124	5,129,511	240,702	79	724,339
	chromosome	NC_007946	1	100x					
**SMS-3-5 (Environmental)**	pSMS35_3	NC_010487	50	5000x	188	5,169,770	200,074	83	377,480
	pSMS35_4	NC_010486	50	5000x					
	pSMS35_8	NC_010485	20	2000x					
	pSMS35_130	NC_010488	1	100x					
	chromosome	NC_010498	1	100x					
**MG1655 + pEC_L46**	pEC_L46	NC_014385	1	100x	146	4,703,719	176,611	83	327,117
	chromosome	NC_000913	1	100x					
**MG1655 + pEC958 + R46**	pEC958	HG941719	1	100x	171	4,761,832	176,611	83	327,117
	R46	NC_003292	1	100x					
	chromosome	NC_000913.3	1	100x					
**O157:H7 str. SS17 (EHEC)**	pO157	CP008807	1	100x	556	5,481,778	179,816	83	386,831
	pSS17	CP008806	2	100x					
	chromosome	CP008805	1	100x					
**O145:H28 str. RM12581 (STEC)**	pRM12581	CP007137	1	100x	646	5,497,629	137,834	79	264,117
	PO145-12581	CP007138	1	100x					
	chromosome	CP007138	1	100x					
**O26:H11 str. 11368 (EHEC)**	pO26_1	NC_013369	1	100x	707	5,616,433	104,130	81	250,500
	pO26_2	NC_013362	1	100x					
	pO26_3	NC_013363	10	1000x					
	pO26_4	NC_014543	10	1000x					
	chromosome	NC_013361	1	100x					
**H0407 (ETEC)**	p52	NC_017721	10	1000x	336	5,205,705	87,499	83	248,902
	p58	NC_017723	10	1000x					
	p666	NC_017722	1	100x					
	p948	NC_017724	1	100x					
	chromosome	NC_017633	1	100x					

[1] The Table shows, for each genome, the accession number for each plasmid and chromosome, the estimated relative copy number of each plasmid, and some details of the simulated assembly of Illumina reads, as explained in the Method section.

[2] ExPEC: Extraintestinal pathogenic *Escherichia coli*, EHEC: Enterohemorrhagic *Escherichia coli*; STEC: Shiga toxin-producing *Escherichia coli;* ETEC: Enterotoxigenic *Escherichia coli.*
