# Supplementary material for: Plasmid Flux in Escherichia coli ST131 Sublineages, Analyzed by Plasmid Constellation Network (PLACNET), a New Method for Plasmid Reconstruction from Whole Genome Sequences
Source: PLoS Genet. 2014 Dec 18;10(12):e1004766. doi: 10.1371/journal.pgen.1004766 (PMC4270462; doi:10.1371/journal.pgen.1004766)
Supplement: S3 Table — PLACNET performance on a set of ten E. coli genomes. (PDF) [file pgen.1004766.s038.pdf]

Table S3. PLACNET performance on a set of ten *E. coli* genomes

Genome[1]	Plasmid	Accession no.	MOB group	Inc group	PLACNET size (bp)	NCBI size (bp)	Delta-size (% error)
**JJ1886 (ST131, ExPEC)**	pJJ1886_1	NC_022661	-	-	1,552	1,552	0 bp (0 %)
	pJJ1886_2	NC_022649	MOB_{Q12}	-	5,167	5,167	0 bp (0 %)
	pJJ1886_3	NC_022662	MOB_{P51}	ColE1-like	5,601	5,631	30 bp (0.5 %)
	pJJ1886_4	NC_022650	MOB_{P11}	IncP1	55,955	55,956	1 bp (0.002 %)
	pJJ1886_5	NC_022651	MOB_{F12}	IncF	104,426	110,040	5,614 bp bp (5 %)
	chromosome	NC_022648	-	-	5,038,008	5,129,938	91,930 bp (2 %)
**UTI89 (ExPEC)**	pUTI89	NC_007941	MOB_{F12}	IncF	113,653	114,230	577 bp (0.5 %)
	chromosome	NC_007946	-	-	5,033,656	5,065,741	32,085 bp (0.6 %)
**SE15 (ST131, ExPEC)**	pECSF1	NC_013655	MOB_{F12}	IncF	116,636	122,345	5,709 bp (5 %)
	chromosome	NC_013654	-	-	4,666,391	4,717,338	50,947 bp (1 %)
**SMS-3-5 (Environmental)**	pSMS35_3	NC_010487	-	-	3,496	3,565	69 bp (2 %)
	pSMS35_4	NC_010486	MOB_{Qu}	-	3,981	4,074	93 bp (2 %)
	pSMS35_8	NC_010485	MOB_{P51}	ColE1-like	8,909	8,909	0 bp (0 %)
	pSMS35_130	NC_010488	MOB_{F12}	IncF	122,954	130,440	7,486 bp (6 %)
	chromosome	NC_010498	-	-	5,039,290	5,068,389	29,099 bp (1 %)
**MG1655 + pEC_L46**	pEC_L46	NC_014385	MOB_{F11} &MOB_{F12}	IncN & IncF	141,163	144,871	3,708 bp (3 %)
	chromosome	NC_000913	-	-	4,582,408	4,641,652	59,244 bp (1 %)
**MG1655 + pEC958 + R46**	pEC958	HG941719	MOB_{F12}	IncF	129,928	135,602	5,674 bp (4 %)
	R46	NC_003292	MOB_{F11}	IncN	41,266	50,969	9,703 bp (19 %)
	chromosome	NC_000913	-	-	4,581,208	4,641,652	60,444 bp (1 %)
**O157:H7 str. SS17 (EHEC)[2]**	pO157	CP008806	$MOB_{P12\text{-}like}$ & ΔTraI-MOB_{F12}	IncF	92,025	94,645	2,620 bp (3 %)
	pSS17	CP008807	MOB_{P6}	IncI2	37,122	37,447	325 bp (0.9 %)
	chromosome	CP008805	-	-	5,293,573	5,523,849	230,276 bp (4 %)
**O145:H28 str. RM12581 (STEC)[3]**	pRM12581	CP007137	-	IncA/C	61,449	64,562	3,113 bp (5 %)
	PO145-12581	CP007138	MOB_{P12}	IncB/O & IncF	74,498	87,120	12,622 bp (15 %)
	chromosome	CP007138	-	-	5,264,714	5,585,611	320,897 bp (6 %)
**O26:H11 str. 11368 (EHEC)[3]**	pO26_1	NC_013369	MOB_{P12}	IncB/O & IncF	74,108	85,167	11,059 bp (13 %)
	pO26_2	NC_013362	MOB_{F12}	IncF	63,357	63,365	8 bp (0.01 %)
	pO26_3	NC_013363	MOB_{P51}	ColE1-like	5,031	5,686	655 bp (12 %)
	pO26_4	NC_014543	-	-	3,690	4,073	383 bp (9 %)
	chromosome	NC_013361	-	-	5,353,548	5,697,240	343,692 bp (6 %)
**H0407 (ETEC)**	p52	NC_017721	MOB_{P51}	ColE1-like	5,175	5,175	0 bp (0 %)
	p58	NC_017723	MOB_{P51}	ColE1-like	5,799	5,800	1 bp (0.02 %)
	p666	NC_017722	MOB_{F12}	IncF	118,831	66,681	-
	p948	NC_017724	-	IncF		94,797	-
	chromosome	NC_017633	-	-	5,070,175	5,153,435	83,260 bp (1.6 %)

[1]ExPEC: Extraintestinal pathogenic Escherichia coli, EHEC: Enterohemorrhagic Escherichia coli; STEC: Shiga toxin-producing Escherichia coli; ETEC: Enterotoxigenic Escherichia coli. [2]In these genomes contigs <350 bp were removed. [3]In this genome contigs <500 bp were removed.
