# Supplementary material for: Plasmid Flux in Escherichia coli ST131 Sublineages, Analyzed by Plasmid Constellation Network (PLACNET), a New Method for Plasmid Reconstruction from Whole Genome Sequences
Source: PLoS Genet. 2014 Dec 18;10(12):e1004766. doi: 10.1371/journal.pgen.1004766 (PMC4270462; doi:10.1371/journal.pgen.1004766)
Supplement: S4 Table — Molecular size of the plasmids estimated by S1-PFGE and PLACNET reconstruction in the four strains sequenced in this study. (PDF) [file pgen.1004766.s039.pdf]

Table S4. Molecular size of the plasmids estimated by S1-PFGE and PLACNET reconstruction in the four strains sequenced in this study.

Strain	S1-PFGE (kb)	PLACNET (kb)
FV9873	80-90; 30	91, 33
E35BA	140; 75	211 (plasmids 1+2)
E2022	100; 95; 35-40	103; 98; 35
E61BA	140; 25; 20; 15	137; 37; 24; 18
