# Supplementary material for: Plasmid Flux in Escherichia coli ST131 Sublineages, Analyzed by Plasmid Constellation Network (PLACNET), a New Method for Plasmid Reconstruction from Whole Genome Sequences
Source: PLoS Genet. 2014 Dec 18;10(12):e1004766. doi: 10.1371/journal.pgen.1004766 (PMC4270462; doi:10.1371/journal.pgen.1004766)
Supplement: S1 Text — The plasmidome of E. coli ST131. (PDF) [file pgen.1004766.s040.pdf]

Text S1: The plasmidome of *E. coli* ST131

This work describes the remarkable heterogeneity of plasmids found in ten *E. coli* ST131 sequenced genomes. We find members of 8 out of the 17 main MOB plasmid groups found in γ-proteobacteria [11,53]. Specifically, we find members of Inc groups F12 (IncF), P3 (IncX), P5 (ColE1), P6 (IncI2), P11 (IncP), P12 (IncI/K/BO), Q12 (Rep_pSC101-like), Qu (Rep_pMG828-2/IGWZ12-like) plus others lacking relaxases that were identifiable as phage-related/RepFIB and no-MOB small plasmids. To date, IncF [15,17,29,56,80,92,93,119, Suzuki et al., 2009, Matsumura et al., 2013], IncFIIK [81], IncI1 [93, Curiao et al., 2011]; IncN [79,120, Suzuki et al., 2009, Zong et al., 2011, Novais et al., 2012] and, sporadically, IncA/C [Naseer et al., 2010, Novais et al., 2012], IncX [94], IncU [Matsumura et al., 2013], IncY [Dhanji et al., 2011], IncK [Calhau et al., 2013], IncIγ [Calhau et al., 2013], IncB/O [Matsumura et al., 2013] and ColE [Matsumura et al., 2012] plasmids were identified in *E. coli* ST131 by PBRT. They were associated with the spread of class A [CTXM (-1,-2, -3, -9, -14, -14, -15, -27, -32, -61), TEM (-4, -24, -116), SHV (-2, -5, -7, -12), NDM (-1), KPC (-2, -3, -4)], class B (VIM-1), class C (CMY-2) and class D (OXA-1) beta lactamases [12,13,23-29] [26,81,83,121, Rogers et al., 2011, Ma et al., 2013, Matsumura et al., 2013, Accogli et al., 2014, Cai et al., 2014] and genes coding for resistance to different antibiotic families (tetracyclines, macrolides, aminoglycosides, trimethoprim and sulfonamides), which are located in composite genomic islands, mostly derived from IncF plasmids (see main text). Despite extensive plasmid analysis of ST131 isolates in different studies, the ST131 plasmidome was not described comprehensively, as most information comes uniquely from identification of antibiotic resistant plasmid replicons by PBRT. As of April 2014, ten plasmids were sequenced from ST131 strains, as shown in Table 3. They include four IncFII, four IncN, one IncI1 and one IncX4 plasmids. Here we provide an individualized discussion of the 8 plasmid groups (39 plasmids) found in our ST131 survey, together with the ten previously sequenced plasmids. This information underscores the relevance of the ST131 plasmidome in the adaptation of this *E. coli* clonal group by providing emergent functions (e.g., antibiotic resistance, virulence, colonization enhancement, ability to outcompete other microbial species) and thus, the usefulness of PLACNET in bacterial population studies, as explained in the main body text of the manuscript.

MOB_{F12}/IncF plasmids (Suppl. Fig. S11). Recent plasmid history suggests that IncF plasmids represent the most abundant family among *E. coli* metapopulations. They are drivers of *E. coli* evolution because of their ability to acquire multiple adaptive traits coding for antibiotic resistance and virulence [2,3,9,82,

Hanni et al., 1982, Womble et al., 1988]. Figure 5 (inset) shows that IncF plasmids identified in the ST131 genomes correspond to four dendrogram groups. The two main groups were Group I (FIB), frequent among avian pathogenic *E. coli* (APEC) and extranintestinal pathogenic *E. coli* (ExPEC), and linked to putative virulence traits and Group II (FII/FIA), widely disseminated among ExPEC and extensively associated with antibiotic resistance (see Table 4). Besides, there were two outlier groups, Group III and Group IV.

Group I (FIB) plasmids mostly comprises virulence plasmids (such as pAPEC-like ones) and four ST131 plasmids containing numerous virulence genes. They are compared in Fig S11A, using ST131 plasmid pJIE186_2 as a reference. Three FIB (ΔFIA:B1) plasmids, pE61BA_1 and pE35BA2+3, were identified in two strains corresponding to ST9/H22/virotype D and ST43/H30/virotype B, respectively. To date, only one report [56] documented FIB plasmid carriage in *E. coli* ST131, despite the identification of putative virulence markers associated with ColV plasmids often appeared in surveys of ST131 *E. coli*[29,36, Kudinha et al., 2013]. Fig S11A emphasizes that all ST131 FIB plasmids (and relatives) share a 80kb genetic island (coordinates 93 kb to 21 kb in the map of Fig S11A) that includes a conserved ColV region comprising *iss, iroBCDEN, iucABCD, iutA, cvaBC* and *sitC* and an *ompT-hlyF-mig14* cassette[55]. While some traits of the conserved region can also be located in the chromosome (linked to PAIs), the cassette *ompT-hlyF-mig14* has only been detected in plasmids[2, Billard-Pomares et al., 2011].

Group II (FII/FIA/FIB) plasmids are compared in Fig S11B, using plasmid pJJ1886_5 as a reference. The seven plasmids analyzed show extensive sequence similarity over at least 100 kb, which includes not only backbone genes but also metabolic, transport and genes associated with mobile genetic elements (MGE). Interestingly, five of the seven plasmids show extensive deletions within their Tra region (see Fig S11B from coord 40 kb to 70 kb). A wide diversity of IncFII/FIA/FIB plasmids carrying *bla* genes have been described among ST131 clonal variants[17,92,93, Fiett et al., 2014], some plasmid types being apparently overrepresented at a global [29] or local level [Doumith et al., 2012, Matsumura et al., 2013] as F2:A1:B- (similar to pEK399) and F2:A-:B- (similar to pC15-1a, pHK01, pEK516), or F29:B10 (identified in ST131 from South Europe). The diversity of replicons, RFLP patterns and sequence mosaicism of *E. coli* ST131 IncF plasmids, even within situations that resemble plasmid outbreak scenarios [17,80,92,119, Smet et al., 2010] suggest that multiple DNA rearrangements among IncF2 plasmids of Enterobacteriaceae are extremely frequent under selection, as described earlier[Hanni et al., 1982, Womble et al., 1988]. More recently, it has been shown that homologous recombination mediated by

IS*26* and Tn*2* between particular ST131 IncF2 plasmids occurs often and partly explains the mentioned diversity[80, Smet et al., 2010]. Recombination of F plasmids among them or with other plasmid groups in enterobacterial populations involved in multispecies outbreaks of *bla*$_{CTX-M-15}$ and *bla*$_{KPC-2}$, confirms rearrangements among large composite regions containing antibiotic resistance genes, transposons and ISs and/or transfer regions of F plasmids is extraordinarily common[Sandegren et al., 2012]. The overrepresentation of F2:A1:B- (pEK499 derivatives) [93, Dhanji et al., 2011]and F2:A-:B- plasmids (e.g. pC15-1a, pHK01, pEK516), the last group only inferred in this study in an H30 strain of "virotype B"[17,29,92, Ho et al., 2012], can reflect the expansion of different *fimH30* ST131 sublineages (here designated as virotypes A, B and C) in different areas.

Group III contains only plasmid pHVH177_1, without antibiotic resistance genes and low content of virulence genes. The BRIG representation (Fig S11C), which compares pHVH177_1 with its closest relatives in the dendrogram of Figure 5, shows conservation of just the MOB$_{F12}$ backbone genes (about 32 kb).

Group IV is represented by plasmid pECSF1, from the commensal ST131 strain SE15. No resistance genes and low content of virulence genes were detected. The most similar plasmids (Fig S11D) are the 122 kb plasmid p1ESCUM[66], the 115 kb plasmid pUM146 (GenBank acc NC_017630) and the 114 kb plasmid pEC14_114[DebRoy et al., 2010]. Besides backbone genes, these plasmids share more than 60 additional kb that includes a number of potential transposable elements. Other close relatives are the 168 kb plasmid pIP1206 [Perichon et al., 2008] and the *K. pneumoniae* MDR plasmid pKF3-140[Zhao et al., 2010].

All these data taken together underscore the complex evolution of MOB$_{F12}$/IncF plasmids, facilitated by the recombinogenic potential of transposable elements[92, Hanni et al., 1982, Womble et al., 1988, Partridge et al., 2004, Johnson et al., 2010].

Phage-related/ RepFIB plasmids (Suppl Fig S12). The ST131 *E.coli* plasmids pBIDMC20B_2 and pBWH24_2, found in two ST43/H30/virotype C isolates, are closely related among them and to the antibiotic resistance plasmid pECOH89 [57] and the STEC plasmid p09EL50[5]. They all share the same RepFIB replication and ParB proteins as backbone remnants, and an extensive set of phage-related proteins (Fig S12A). This group also comprises other phage-related plasmids such as plasmid pLF82 [58] from the prototype strain of adherent invasive *E. coli* (AIEC), the *Salmonella* plasmid pHCM2[59], the

Salmonella bacteriophage SSU5 [60] and the *Yersinia pestis* pMT1 and MT plasmids[Hu et al., 1999]. An extensive set of phage-related genes are shared by all seven plasmids. As shown in Fig S12A, only pECOH89 plasmid harbors the *bla*$_{CTX-M-15}$ antibiotic resistance gene. As shown in the phylogenetic tree of Fig S12B, the RIP protein RepFIB (Rep3_superfamily, pfam 01051) from ST131 plasmids is identical to those of plasmids pECOH89 and p09EL50 (here named as Group C). They are only 40% identical to Group B proteins, represented by plasmids pLF82, pHCM2, pMT1 and MT. Group A in the figure is represented by the well-known IncF (FIB) plasmids discussed in the previous section. At this point it must be stressed that, although a majority of plasmids carrying RepFIB belong to the IncF group, RepFIB also appears in more than 20 other RIP groups such as IncN, IncP or IncI groups[Gibbs et al., 1993]. Further work is required to rigorously classify RepFIB proteins (all belonging to the Rep3-superfamily) according to plasmid RIP groups. Moreover, the biology of RepFIB/phage-related plasmids remains largely unexplored, although they might be more extended than previously thought[57].

MOB$_{P12}$/IncI-complex (Suppl Fig S13). The single MOB$_{P12}$/IncI representative in our collection is plasmid pE2022_1, most similar to the IncI1/ST16 plasmid pEK204, carrying *bla*$_{CTX-M-15}$, already isolated from a ST131 strain[93]. Plasmids pEK204 and pE2022_1 are not phylogenetically close among themselves. Rather, they are similar to widespread IncI1 (*bla*$_{CTX-M-15}$) plasmids from Europe and IncK (*bla*$_{CTX-M-14}$) plasmids from Asia, respectively[16,93, Zang et al., 2013, Onnberg et al., 2014].

The IncI1 plasmid pEK204 shares its backbone with the eight IncI1 (also called Incα) plasmids used as references in Fig S13A. While pEK204 carries (*bla*$_{CTX-M-15}$ and *bla*$_{TEM}$), these antibiotic resistance genes are absent in its closest relatives (see coord. 6 kb and 14 kb in S13A). Plasmids in this cluster include the Incγ prototype, the 93.2 kb plasmid R621a from *S. enterica* [Takahashi et al., 2011].

The ST131 plasmid pE2022_1 (98.3 kb) is similar to the IncK prototype 93.6 kb plasmid pCT [61]. They share more than 80 kb including Tra regions and the *bla*$_{CTX-M-14}$ gene coding for resistance to beta-lactam antibiotics. IncK pCT-like plasmids harboring *bla*$_{CTX-M-14}$ are globally spread among animals and humans, being prevalent in UK and Spain among other countries [63,64, Dhanji et al., 2011]. Although widely spread, they have been only sporadically described among ST131 isolates, since IncF plasmids are the main vectors carrying *bla*$_{CTX-M-15}$ and *bla*$_{CTX-M-14}$ genes in ST131 to date.

MOB$_{P6}$/IncI2 plasmids (Suppl Fig S14). The two IncI2 plasmids found in our ST131 genomes (pBWH24_3 and pE61BA_7) belong to different clusters of a large plasmid family that, however, contains few

sequenced plasmids. Fig S14A shows the phylogenetic tree of MOB_{P6} REL proteins, underscoring the distant positions of the ST131 plasmid REL proteins. Plasmid pBWH24_3 (60.3 kb) is similar to the *E.coli* IncI2 plasmid prototype R721 (75.6 kb; [63]) and the *E.coli* APEC plasmid pChi7122_3 (56.7 kb, GenBank acc FR851304) over most of their length (Fig S14B). Plasmid R721 is the representative of a number of enterobacterial plasmids commonly isolated from *K. pneumoniae* and associated with spread of antibiotic resistance, mainly bla_{CMY-2} and bla_{KPC-2} [83]. Plasmids pBWH24_3 and pChi7122_3, on the other hand, lack any antibiotic resistance gene. These two plasmids are highly similar, the last one having a role in acid resistance and biofilm formation [64]. Besides, it is of note that IncI2 plasmids have a high number of putative integration sites, which would facilitate the acquisition of different antibiotic resistance genes, resulting in large composite multidrug platforms[83].

Plasmid pE61BA_7 (37.9 kb) is most similar (Fig S14C) to the *S. enterica* plasmid SL483 (37.9kb; [Fricke et al., 2011] and *E. coli* plasmid pO157_Sal (37.8 kb; [65]). Plasmid pE61BA_7 belongs to a different IncI2 subcluster than pBWH24_3, as shown in Fig S14A. This group is composed of cryptic plasmids, represented by SL483 from *Salmonella agona* and pO157_Sal from *E. coli* O157:H5, and seems to be widely spread among enterobacterial isolates from animals [65]. IncI2 plasmids have been identified among CTX-M-15 producers of the ST131/H30 sublineage collected in Canada, Australia and New Zealand [16] but they were not fully characterized in any of those studies.

MOB_{P3} /IncX plasmids (Suppl Fig S15). IncX plasmids are prevalent in *E. coli* and belong to a large plasmid family with four recognized subgroups X1 to X4 [Johnson et al., 2012]. The two IncX plasmids identified in this survey belong to the IncX1 (pFV9873_4) and IncX4 (pE2022_3) subclusters (Fig S15A). To these, the previously reported IncX4 plasmid pJIE143 should be added. As shown in Fig S15B, the IncX1 plasmid pFV9873_4 (33.3 kb) was most similar to the 33.8 kb plasmid p2ESCUM [66] over its entire length, and no so much to other reference hits. p2ESCUM was originally isolated from the emblematic strain UMN026, representative of a widely disseminated urinary pathogenic ST69 *E. coli* (UPEC) clone [Lescat et al., 2009]. The IncX4 plasmid pE2022_3 (35.0 kb) shares extensive homology with the *S. enterica* plasmid pSH696_34 (33.8 kb, [Gokulan et al., 2013]), which is used as a reference in Fig S15C. Compared to these two plasmids, the ST131 reference plasmid pJIE143 (34.3 kb;[94]), widely disseminated among *E. coli* from foodborne animals, lacks two important backbone genes, the conjugative coupling protein gene *traG* (around coord 19 kb) and the RIP encoding region (coord 9 kb to 10 kb). Plasmid pJIE143 codes for a completely different Pir protein (13% amino acid identity) although

also belonging to the Rep3 family. Therefore, it is probable that pSH696_34 and pE2022_3 belong to a different incompatibility group than pJIE143. IncX4 plasmids can be transferred at different temperatures and are frequent vehicles of antibiotic resistance, mainly *qnr*. Difficulties for detecting this plasmid group using PBRT might have underestimated its prevalence.

MOB_{P11}/IncP1 plasmids (Suppl Fig S16). Members of the IncP1 family are frequently isolated in the environment and often carry modules containing diverse accessory genes can be transferred between unrelated bacteria [Schluter et al., 2007, Venturini et al., 2013]. IncP1 plasmids from *E. coli* remain largely unexplored although detection of IncP sequences from animal [Dotto et al., 2014] and human *E. coli* isolates, included ST131 [16], suggest that IncP1 plasmids can be easily acquired by human associated Proteobacteria. As can be observed in the phylogenetic tree of Fig S16A, plasmids pJJ1886_4 and pE61BA_4 represent different new branches of the MOB_{P11} family and thus new additions to the ST131 plasmidome. The pJJ1886_4 REL protein is most similar to that of the 40.6 kb plasmid pDS1. However, proteome analysis resulting in the dendrogram of Fig. 5, shows plasmid pHS102707 (*E. coli*, 69.5 kb, Genbank acc NC_023907) as its closest homolog. This is confirmed in Fig S16B, where the extensive homology of pJJ1886_4 and pHS102707 is depicted. The second branch of the phylogenetic tree contains the ST131 plasmid pE61BA_4 (18.3 kb). The 37.9 kb plasmid pMBUI2 [67], from an uncultured bacterium, appears as its closest homolog at the level of REL protein. Plasmid pE61BA_4 seems a crippled plasmid, lacking most of its backbone, with just four genes shared with pMBUI2 (Suppl. Table S1), suggesting that it is the first representative of a new plasmid group. Finally, the same phylogenetic branch contains IME_E35BA REL protein. To analyze this integrative and mobilizable element (IME), we compared the relevant regions of E35BA and JJ1886 genomes using EasyFig [Sullivan et al., 2011]. Fig S16C shows the IME_E35BA (14.2 kb) inserted between a hypothetical protein and a GMP synthase encoding genes in *E.coli* JJ1886. IME_E35BA is similar to the *Burkholderia glumae* IncP island (14.7 kb; [Yoshii et al., 2012]). Both share a *tra* conjugative region, *repA*, *alpA* (encoding a DNA binding protein) and *int-P4* (coding for a P4 phage integrase family protein). They differ in their *repC* (resolvase) genes. Besides, IME_E35BA does not harbor the kasugamycin-2'-N-acetyltransferase gene [aac(2')-Iia] present in the *Bukholderia glumae* strain that confers resistance to kasugamycin, an aminoglycoside antibiotic widely used in agriculture [Yoshii et al., 2012].

MOB_{C12} plasmids (Suppl Fig S17). The MOB_C family constitutes a wide plasmid group, present in γ-proteobacteria, Firmicutes and Tenericutes. MOB_C relaxases corresponding to γ-proteobacterial

plasmids are clustered in the MOB_{C1} group, subdivided as well in two branches: MOB_{C11} and MOB_{C12}[11, Garcillan-Barcia et al., 2009]. Plasmid pE61BA_2 (24.5kb), located in a ST131 ST9/H234 *E.coli* strain, is not close to any reference from Figure 5, but clusters in the MOB_{C12} group according to its REL (Fig S17A). pE61BA_2 REL protein is only 70% identical to the closest hits: the 22 kb *Y. pestis* cryptic plasmid pCRY [Song et al., 2004] and the 42 kb *K.pneumoniae* multidrug resistant (MDR) plasmid pMET1 [Soler Bistue et al., 2008]. As shown in Fig S17B, pE61BA_2 shares mobilization (*mobBC –virB*) and replication (*repA*) genes with pCRY and pMET1, but lacks the micrococcal nuclease-like and the Mpr coding genes. Since PBRT does not detect MOB_C plasmids, they are probably underrepresented in the plasmidome of Enterobacteriaceae.

MOB_{P5}/ColE plasmids (Suppl Fig S18). The MOB_{P5} REL phylogenetic tree shows three different branches, constituting the MOB_{P51}, MOB_{P52} and MOB_{P53} subfamilies [11]. The four ST131 MOB_{P5} plasmids are located in the MOB_{P51} subfamily (Fig S18A). Plasmid pE61BA_5 (6.6 kb) is similar to the prototype MOB_{P5} plasmid ColE1 (6.6 kb) [Tomizawa et al., 1977] over their entire length (including MOB proteins and colicin-E1), as shown in Fig S18B. The ST131 plasmids pBIDMC38_1 (11.8 kb) and pJJ1886_3 (5.6 kb) are very similar among them, including their MOB and RIP proteins (Fig S18C). They are unique in carrying a large (2 kb) gene encoding an intriguing EamA-like transporter protein (pfam00892). Finally, plasmid pE61BA_6 (6.9 kb) is similar to enterohemorrhagic *E. coli* (EHEC) O157 plasmid pColD-157 (6.7 kb, [Hofinger et al., 1998]) and colicin K plasmid pColK-K235 (8.3 kb, [Rijavec et al., 2007]). As Fig S18D shows, pE61BA_6 plasmid shares MbkABCD mobilization and colicin K encoding regions with the pColK-K235 reference plasmid.

Although widely used as cloning vectors for genetic engineering, ColE plasmids represent a largely unexplored family in epidemiological studies, since they not usually carry genes coding for antibiotic resistance. On the other hand, they frequently, but not always, carry colicins [68]. The MOB_{P5}/ColE1-like plasmids identified in this study exhibited a wide size range (5.6-10.5 kb) and variable presence of colicins, two of them carrying either ColE1 or ColK, both in the same strain (Suppl figures S18A-D). These two colicins have traditionally been associated with UPEC and the B2 phylogenetic group of *E. coli* [68, Rijavec et al., 2007]. Although the carriage of ColE-1 like plasmids, inferred by the presence of either RIP or REL sequences, seems to be frequent among non-outbreak O25b and non-O25b ST131 isolates [Matsumura et al., 2013], the lack of full plasmid sequences and functional information preclude any conclusion about a role in the pathogenesis of B2 and ST131 clones. Besides a possible involvement of

colicin production in virulence, perhaps as a competition weapon to avoid the presence of other *E. coli* pathogenic strains [Budic et al., 2011], ColE1 plasmids can promote rearrangements and contribute to the mobilization of other plasmids [Rijavec et al., 2007]. As an example, the large plasmid (pBIDMC38_1) is almost identical to the ST131 reference pJJ1886_3, with an additional type II restriction-modification system (Cfr10I) (Fig. S18). In spite of the above, ColE plasmids carrying genes encoding antibiotic resistance are increasingly being reported although by lack of phylogenetic analysis, they cannot be accurately classified within the MOB_{P5} group [10, Dotto et al., 2014].

MOB_{Qu} plasmids (Suppl Fig S19). MOB_{Qu} and MOB_{Q12} plasmid clusters were previously identified by our group [11,53]. They have scarcely been documented among *E. coli* to date, probably because they do not carry antimicrobial resistance genes. This report is the first to document their presence in the ST131 lineage. Nevertheless, they seem to be common among UPEC, as shown in this study. The four MOB_{Qu} plasmids identified in this study were almost identical among them and with pSE11_6 [Oshima et al., 2008] in a DNA stretch of 4 kb, as shown in Fig S19B. Apparently they only code for RIP and MOB proteins. The role of these cryptic plasmids in the adaptation of ST131 is intriguing. Although not explored at all, a role in the adaptation by providing useful mobilization tools cannot be excluded.

MOB_{Q12} plasmids (Suppl Fig S20). As seen in the REL phylogenetic tree of fig S20A, the four ST131 MOB_{Q12} plasmids have almost identical relaxases. Three of the four MOB_{Q12} plasmids (of about 5.2 kb) (pBIDMC38_2, pFV9873_6 and pJJ1886_2) were almost identical among them and to the UPEC plasmid pCE10B [Lu et al., 2011], as shown in Fig S20B. They constitute a cluster of plasmids within the MOB_{Q12} family previously defined by the authors [11,53]. The fourth plasmid (pE61BA_3; 5.5 kb) has a unique 1.6 kb DNA segment that codes for a 266 amino acid protein identical to a colicin from *S. enterica* (NCBI protein acc. WP_024155916). These plasmids frequently coexist with IncF plasmids and form cointegrates with them (our unpublished data).

Small cryptic no-MOB plasmids (Suppl Fig S21). Small non-mobilizable cryptic plasmids remain scarcely documented among Enterobacteriaceae. Four highly similar plasmids of about 1.6 to 2.2 kb were found in our study. They are also similar to the 1.5 kb *E. coli* plasmid pCE10D [Lu et al., 2011], as shown in Fig S21. As can be expected, they have minimal coding capacity. Their only annotated gene codes for a RIP protein of the Rep_HTH_36_superfamily (pfam13730). Similar plasmids have also been detected in various ExPEC, including ST131 [90], Shiga-toxin producing *E. coli* (STEC) [Lu et al., 2011, Brolund et al.,

2013] and *Klebsiella* [Liu et al., 2012] isolates. A fifth, 5kb no-MOB plasmid (pFV9873_3) was unique and unrelated to any reference plasmid (see Figure 5). The adaptive functions of these plasmids, if any, are unknown.

Other ST131 plasmid groups: MOB_{F11}/IncN plasmids (Suppl. Fig. S22). We carried out extensive literature analysis in the search of plasmid groups present in ST131 but not detected in our survey. There were two plasmid groups, IncN and IncA/C, for which no plasmid was identified in our study although they are being increasingly detected among ST131 isolates. Both groups have been associated with bla_{CTX-M} [120, Novais et al., 2012] and more recently with bla_{KPC} [89,81,121, Matsumura et al., 2013] and bla_{NDM} genes (GenBank acc nº KJ413946). These plasmids have spread worldwide and might have been acquired by ST131 from other enterobacterial species, e.g., during nosocomial polyclonal outbreaks [85].

Four complete sequences of MOB_{F11}/IncN plasmids originating from ST131 strains are available: the IncN1 plasmids pECN580 [79], pKC394 and pKC396 [120] (GenBank acc nº HM138652 and HM138653, respectively) and the IncN2 plasmid pNDM-ECS01 (GenBank acc nº KJ413946). Suppl Fig S22A shows the REL phylogenetic tree of the MOB_{F11}/IncN plasmid branch, which displays the IncN1 and IncN2 subclusters. BRIG comparative analysis of the three ST131 IncN1 plasmids versus reference plasmids (Suppl Fig S22B) reveals a highly conserved 34kb backbone region (coordinates 0kb to 34kb). As previously reported [11], IncN1 and IncN2 plasmid backbones, including REL proteins, are homologous but their RIP proteins are different [Poirel et al., 2011, Partridge et al, 2012]. Accordingly, the IncN2 pNDM-ECS01 plasmid RIP has no similarity to the RIPs from pECN580, pKC394 or pKC396 IncN1 plasmids. Comparative analysis of the pNDM-ECS01 plasmid (Suppl Fig S22C), harboring the bla_{NDM-1} gene, versus the reference plasmids p271A [Poirel et al, 2011], pTR3 and pTR4 [Chen et al., 2012] and pJIE137 [Partridge et al., 2012] shows that all are almost identical except pJIE137, which harbors the $bla_{CTX-M-62}$ gene. Despite of the low prevalence of IncN1 and IncN2 plasmids in ST131 *E.coli* strains, these plasmids seem to be good vehicles for the transmission of several antibiotic resistance genes in Enterobacteriaceae by maintaining conserved backbones but variable regions that harbor genes encoding CTX-M, KPC or NDM beta-lactamases.

Supplemental references

Suzuki S, Shibata N, Yamane K, Wachino J, Ito K, et al. (2009) Change in the prevalence of extended-spectrum-beta-lactamase-producing Escherichia coli in Japan by clonal spread. J Antimicrob Chemother 63: 72-79.

Matsumura Y, Yamamoto M, Nagao M, Ito Y, Takakura S, et al. (2013) Association of fluoroquinolone resistance, virulence genes, and IncF plasmids with extended-spectrum-beta-lactamase-producing Escherichia coli sequence type 131 (ST131) and ST405 clonal groups. Antimicrob Agents Chemother 57: 4736-4742.

Curiao T, Canton R, Garcillan-Barcia MP, de la Cruz F, Baquero F, et al. (2011) Association of composite IS26-sul3 elements with highly transmissible IncI1 plasmids in extended-spectrum-beta-lactamase-producing Escherichia coli clones from humans. Antimicrob Agents Chemother 55: 2451-2457.

Zong Z, Yu R, Wang X, Lu X (2011) blaCTX-M-65 is carried by a Tn1722-like element on an IncN conjugative plasmid of ST131 Escherichia coli. J Med Microbiol 60: 435-441.

Novais A, Viana D, Baquero F, Martinez-Botas J, Canton R, et al. (2012) Contribution of IncFII and broad-host IncA/C and IncN plasmids to the local expansion and diversification of phylogroup B2 Escherichia coli ST131 clones carrying blaCTX-M-15 and qnrS1 genes. Antimicrob Agents Chemother 56: 2763-2766.

Naseer U, Haldorsen B, Simonsen GS, Sundsfjord A (2010) Sporadic occurrence of CMY-2-producing multidrug-resistant Escherichia coli of ST-complexes 38 and 448, and ST131 in Norway. Clin Microbiol Infect 16: 171-178.

Dhanji H, Doumith M, Rooney PJ, O'Leary MC, Loughrey AC, et al. (2011) Molecular epidemiology of fluoroquinolone-resistant ST131 Escherichia coli producing CTX-M extended-spectrum beta-lactamases in nursing homes in Belfast, UK. J Antimicrob Chemother 66: 297-303.

Calhau V, Ribeiro G, Mendonca N, Da Silva GJ (2013) Prevalent combination of virulence and plasmidic-encoded resistance in ST 131 Escherichia coli strains. Virulence 4: 726-729.

Cai JC, Zhang R, Hu YY, Zhou HW, Chen GX (2014) Emergence of Escherichia coli sequence type 131 isolates producing KPC-2 carbapenemase in China. Antimicrob Agents Chemother 58: 1146-1152.

Rogers BA, Sidjabat HE, Paterson DL (2011) Escherichia coli O25b-ST131: a pandemic, multiresistant, community-associated strain. J Antimicrob Chemother 66: 1-14.

Ma L, Siu LK, Lin JC, Wu TL, Fung CP, et al. (2013) Updated molecular epidemiology of carbapenem-non-susceptible Escherichia coli in Taiwan: first identification of KPC-2 or NDM-1-producing E. coli in Taiwan. BMC Infect Dis 13: 599.

Accogli M, Giani T, Monaco M, Giufre M, Garcia-Fernandez A, et al. (2014) Emergence of Escherichia coli ST131 sub-clone H30 producing VIM-1 and KPC-3 carbapenemases, Italy. J Antimicrob Chemother.

Hanni C, Meyer J, Iida S, Arber W (1982) Occurrence and properties of composite transposon Tn2672: evolution of multiple drug resistance transposons. J Bacteriol 150: 1266-1273.

Womble DD, Rownd RH (1988) Genetic and physical map of plasmid NR1: comparison with other IncFII antibiotic resistance plasmids. Microbiol Rev 52: 433-451.

Kudinha T, Johnson JR, Andrew SD, Kong F, Anderson P, et al. (2013) Distribution of phylogenetic groups, sequence type ST131, and virulence-associated traits among Escherichia coli isolates from men with pyelonephritis or cystitis and healthy controls. Clin Microbiol Infect 19: E173-180.

Billard-Pomares T, Tenaillon O, Le Nagard H, Rouy Z, Cruveiller S, et al. (2011) Complete nucleotide sequence of plasmid pTN48, encoding the CTX-M-14 extended-spectrum beta-lactamase from an Escherichia coli O102-ST405 strain. Antimicrob Agents Chemother 55: 1270-1273.

Fiett J, Baraniak A, Izdebski R, Sitkiewicz I, Zabicka D, et al. (2014) The first NDM metallo-beta-lactamase-producing Enterobacteriaceae isolate in Poland: evolution of IncFII-type plasmids carrying the bla(NDM-1) gene. Antimicrob Agents Chemother 58: 1203-1207.

Doumith M, Dhanji H, Ellington MJ, Hawkey P, Woodford N (2012) Characterization of plasmids encoding extended-spectrum beta-lactamases and their addiction systems circulating among Escherichia coli clinical isolates in the UK. J Antimicrob Chemother 67: 878-885.

Smet A, Van Nieuwerburgh F, Vandekerckhove TT, Martel A, Deforce D, et al. (2010) Complete nucleotide sequence of CTX-M-15-plasmids from clinical Escherichia coli isolates: insertional events of transposons and insertion sequences. PLoS One 5: e11202.

Sandegren L, Linkevicius M, Lytsy B, Melhus A, Andersson DI (2012) Transfer of an Escherichia coli ST131 multiresistance cassette has created a Klebsiella pneumoniae-specific plasmid associated with a major nosocomial outbreak. J Antimicrob Chemother 67: 74-83.

Ho PL, Yeung MK, Lo WU, Tse H, Li Z, et al. (2012) Predominance of pHK01-like incompatibility group FII plasmids encoding CTX-M-14 among extended-spectrum beta-lactamase-producing Escherichia coli in Hong Kong, 1996-2008. Diagn Microbiol Infect Dis 73: 182-186.

DebRoy C, Sidhu MS, Sarker U, Jayarao BM, Stell AL, et al. (2010) Complete sequence of pEC14_114, a highly conserved IncFIB/FIIA plasmid associated with uropathogenic Escherichia coli cystitis strains. Plasmid 63: 53-60.

Perichon B, Bogaerts P, Lambert T, Frangeul L, Courvalin P, et al. (2008) Sequence of conjugative plasmid pIP1206 mediating resistance to aminoglycosides by 16S rRNA methylation and to hydrophilic fluoroquinolones by efflux. Antimicrob Agents Chemother 52: 2581-2592.

Zhao F, Bai J, Wu J, Liu J, Zhou M, et al. (2010) Sequencing and genetic variation of multidrug resistance plasmids in Klebsiella pneumoniae. PLoS One 5: e10141.

Partridge SR, Hall RM (2004) Complex multiple antibiotic and mercury resistance region derived from the r-det of NR1 (R100). Antimicrob Agents Chemother 48: 4250-4255.

Johnson TJ, Jordan D, Kariyawasam S, Stell AL, Bell NP, et al. (2010) Sequence analysis and characterization of a transferable hybrid plasmid encoding multidrug resistance and enabling zoonotic potential for extraintestinal Escherichia coli. Infect Immun 78: 1931-1942.

Hu P, Elliott J, McCready P, Skowronski E, Garnes J, et al. (1998) Structural organization of virulence-associated plasmids of Yersinia pestis. J Bacteriol 180: 5192-5202.

Gibbs MD, Spiers AJ, Bergquist PL (1993) RepFIB: a basic replicon of large plasmids. Plasmid 29: 165-179.

Zhang L, Lu X, Zong Z (2013) The emergence of blaCTX-M-15-carrying Escherichia coli of ST131 and new sequence types in Western China. Ann Clin Microbiol Antimicrob 12: 35.

Onnberg A, Soderquist B, Persson K, Molling P (2014) Characterization of CTX-M-producing Escherichia coli by repetitive sequence-based PCR and real-time PCR-based replicon typing of CTX-M-15 plasmids. APMIS.

Takahashi H, Shao M, Furuya N, Komano T (2011) The genome sequence of the incompatibility group Igamma plasmid R621a: evolution of IncI plasmids. Plasmid 66: 112-121.

Fricke WF, Mammel MK, McDermott PF, Tartera C, White DG, et al. (2011) Comparative genomics of 28 Salmonella enterica isolates: evidence for CRISPR-mediated adaptive sublineage evolution. J Bacteriol 193: 3556-3568.

Johnson TJ, Bielak EM, Fortini D, Hansen LH, Hasman H, et al. (2012) Expansion of the IncX plasmid family for improved identification and typing of novel plasmids in drug-resistant Enterobacteriaceae. Plasmid 68: 43-50.

Lescat M, Calteau A, Hoede C, Barbe V, Touchon M, et al. (2009) A module located at a chromosomal integration hot spot is responsible for the multidrug resistance of a reference strain from Escherichia coli clonal group A. Antimicrob Agents Chemother 53: 2283-2288.

Gokulan K, Khare S, Rooney AW, Han J, Lynne AM, et al. (2013) Impact of plasmids, including those encodingVirB4/D4 type IV secretion systems, on Salmonella enterica serovar Heidelberg virulence in macrophages and epithelial cells. PLoS One 8: e77866.

Schluter A, Szczepanowski R, Puhler A, Top EM (2007) Genomics of IncP-1 antibiotic resistance plasmids isolated from wastewater treatment plants provides evidence for a widely accessible drug resistance gene pool. FEMS Microbiol Rev 31: 449-477.

Venturini C, Hassan KA, Roy Chowdhury P, Paulsen IT, Walker MJ, et al. (2013) Sequences of two related multiple antibiotic resistance virulence plasmids sharing a unique IS26-related molecular signature isolated from different Escherichia coli pathotypes from different hosts. PLoS One 8: e78862.

Dotto G, Giacomelli M, Grilli G, Ferrazzi V, Carattoli A, et al. (2014) High prevalence of oqxAB in Escherichia coli isolates from domestic and wild lagomorphs in Italy. Microb Drug Resist 20: 118-123.

Sullivan MJ, Petty NK, Beatson SA (2011) Easyfig: a genome comparison visualizer. Bioinformatics 27: 1009-1010.

Yoshii A, Moriyama H, Fukuhara T (2012) The novel kasugamycin 2'-N-acetyltransferase gene aac(2')-IIa, carried by the IncP island, confers kasugamycin resistance to rice-pathogenic bacteria. Appl Environ Microbiol 78: 5555-5564.

Garcillan-Barcia MP, Francia MV, de la Cruz F (2009) The diversity of conjugative relaxases and its application in plasmid classification. FEMS Microbiol Rev 33: 657-687.

Song Y, Tong Z, Wang J, Wang L, Guo Z, et al. (2004) Complete genome sequence of Yersinia pestis strain 91001, an isolate avirulent to humans. DNA Res 11: 179-197.

Soler Bistue AJ, Birshan D, Tomaras AP, Dandekar M, Tran T, et al. (2008) Klebsiella pneumoniae multiresistance plasmid pMET1: similarity with the Yersinia pestis plasmid pCRY and integrative conjugative elements. PLoS ONE 3: e1800.

Tomizawa JI, Ohmori H, Bird RE (1977) Origin of replication of colicin E1 plasmid DNA. Proc Natl Acad Sci U S A 74: 1865-1869.

Hofinger C, Karch H, Schmidt H (1998) Structure and function of plasmid pColD157 of enterohemorrhagic Escherichia coli O157 and its distribution among strains from patients with diarrhea and hemolytic-uremic syndrome. J Clin Microbiol 36: 24-29.

Rijavec M, Budic M, Mrak P, Muller-Premru M, Podlesek Z, et al. (2007) Prevalence of ColE1-like plasmids and colicin K production among uropathogenic Escherichia coli strains and quantification of inhibitory activity of colicin K. Appl Environ Microbiol 73: 1029-1032.

Budic M, Rijavec M, Petkovsek Z, Zgur-Bertok D (2011) Escherichia coli bacteriocins: antimicrobial efficacy and prevalence among isolates from patients with bacteraemia. PLoS One 6: e28769.

Oshima K, Toh H, Ogura Y, Sasamoto H, Morita H, et al. (2008) Complete genome sequence and comparative analysis of the wild-type commensal Escherichia coli strain SE11 isolated from a healthy adult. DNA Res 15: 375-386.

Lu S, Zhang X, Zhu Y, Kim KS, Yang J, et al. (2011) Complete genome sequence of the neonatal-meningitis-associated Escherichia coli strain CE10. J Bacteriol 193: 7005.

Brolund A, Franzen O, Melefors O, Tegmark-Wisell K, Sandegren L (2013) Plasmidome-analysis of ESBL-producing escherichia coli using conventional typing and high-throughput sequencing. PLoS One 8: e65793.

Liu P, Li P, Jiang X, Bi D, Xie Y, et al. (2012) Complete genome sequence of Klebsiella pneumoniae subsp. pneumoniae HS11286, a multidrug-resistant strain isolated from human sputum. J Bacteriol 194: 1841-1842.

Poirel L, Bonnin RA, Nordmann P (2011) Analysis of the resistome of a multidrug-resistant NDM-1-producing Escherichia coli strain by high-throughput genome sequencing. Antimicrob Agents Chemother 55: 4224-4229.

Partridge SR, Paulsen IT, Iredell JR (2012) pJIE137 carrying blaCTX-M-62 is closely related to p271A carrying blaNDM-1. Antimicrob Agents Chemother 56: 2166-2168.

Chen YT, Lin AC, Siu LK, Koh TH (2012) Sequence of closely related plasmids encoding bla(NDM-1) in two unrelated Klebsiella pneumoniae isolates in Singapore. PLoS One 7: e48737.
