# Supplementary material for: Plasmid Flux in Escherichia coli ST131 Sublineages, Analyzed by Plasmid Constellation Network (PLACNET), a New Method for Plasmid Reconstruction from Whole Genome Sequences
Source: PLoS Genet. 2014 Dec 18;10(12):e1004766. doi: 10.1371/journal.pgen.1004766 (PMC4270462; doi:10.1371/journal.pgen.1004766)

Figure S28

Plasmid 5 (p5) : 104,426 bp

Reference plasmid pJJ1886_5 : 110,040 bp

Delta-size (% error): 5,614 bp (5 %)

MOB_{F12}, IncF plasmid

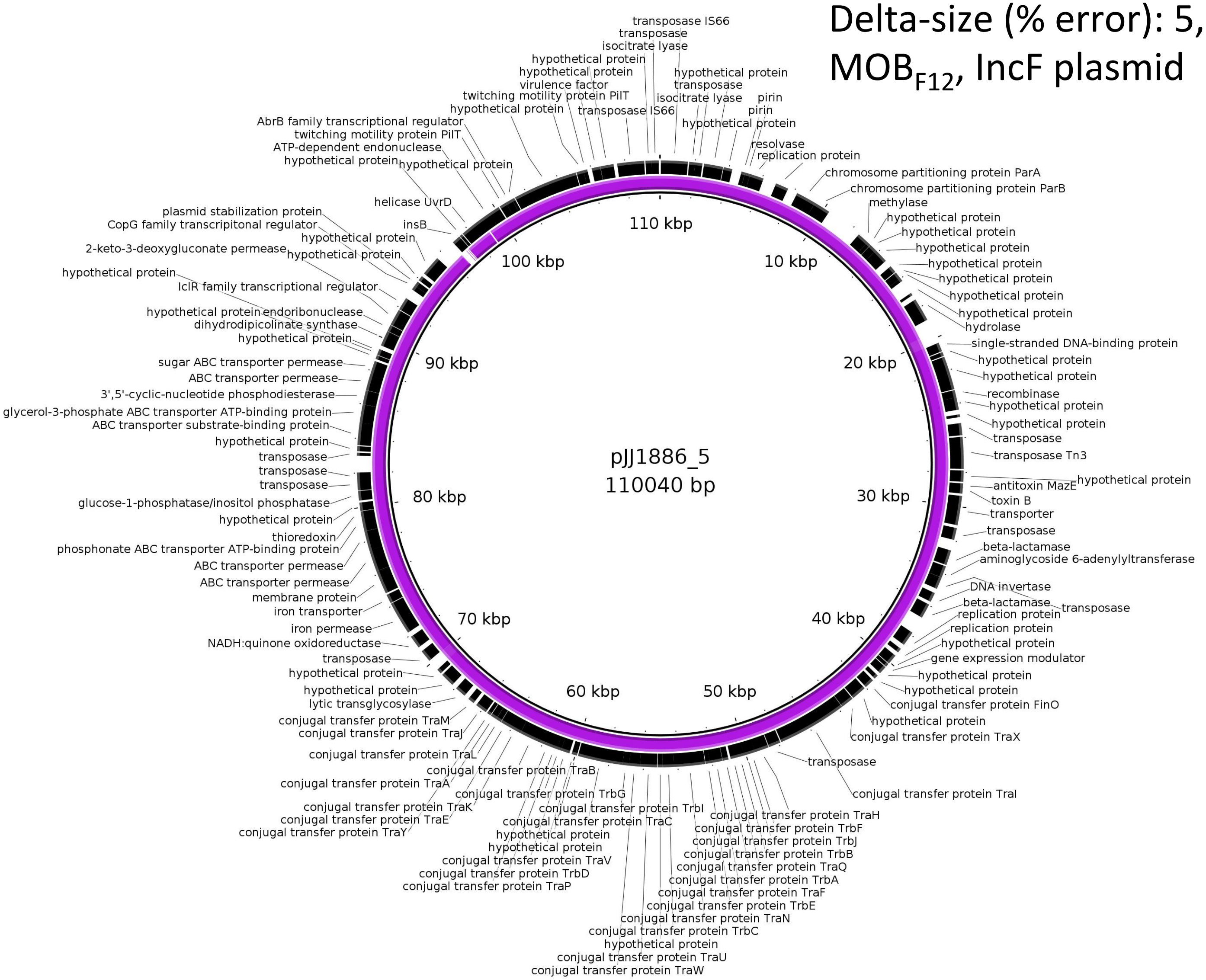

Supplement: S28 Fig — BRIG comparison of the reconstructed plasmid p5 with the reference plasmid pJJ1886_5. The reference plasmid (inner ring) is compared to the p5 reconstructed plasmid (purple ring). Outer black and white ring sectors represent pJJ1886_5 gene annotations. (PDF) [file pgen.1004766.s028.pdf]
