# Supplementary material for: Plasmid Flux in Escherichia coli ST131 Sublineages, Analyzed by Plasmid Constellation Network (PLACNET), a New Method for Plasmid Reconstruction from Whole Genome Sequences
Source: PLoS Genet. 2014 Dec 18;10(12):e1004766. doi: 10.1371/journal.pgen.1004766 (PMC4270462; doi:10.1371/journal.pgen.1004766)
Supplement: S2 Text — PLACNET validation by reconstruction of finalized genomes. (PDF) [file pgen.1004766.s041.pdf]

Text S2: PLACNET validation by reconstruction of finalized genomes

Methods

The objective of this report is the validation of PLACNET by plasmid reconstruction in genomes that were published as complete, finished genomes. The ten analyzed genomes are shown in **Table S2**. Genome sequences were downloaded from NCBI in FASTA format. As a consequence of using FASTA, all sequences were considered as linear DNA sequences. The ART software (Huang et al., 2012) was used to simulate pair-end Illumina reads. Main parameters used were: read length, 101 bp; insert size, 400 bp; standard deviation of fragment size for pair end, 10 bp. For each plasmid, the relative coverage was estimated according to its reported or estimated copy number, as shown in **Table S2**. Resulting reads were analyzed using the workflow shown in **Figure 6** of the main text, strictly following the same steps as in plasmid reconstruction of real data. For the analysis of each individual genome, the query genome sequences (including plasmids) were subtracted from the local megablast NCBI database. For each reconstructed plasmid, we define the ***error rate*** as the % of total DNA sequence of the finalized plasmid that is lost (or misplaced) in the plasmid reconstruction.

Besides, DNA from strains FV9873, E35BA, E2022 and E61BA was extracted as explained in Materials and Methods of Valverde et al., 2009. DNA samples were digested with S1-nuclease and visualized after Pulsed-Field Gel Elecrophoresis (PFGE) using exactly the conditions described by Valverde et al., 2009.

Exemplar reconstruction of a multi-plasmid genome: *E. coli* ST131 ExPEC strain JJ1886

Figure S25 shows the original and pruned Cytoscape networks of the JJ1886 genome, constructed as explained in Materials and Methods. PLACNET-based plasmid reconstruction is summarized in **Figures S26** and **S27**. **Figure S26** shows plasmid definition in the pruned network. Four single contigs (surrounded by colored circles in the figure) represent plasmids p1 to p4, since they contain a Replication Initiator Protein (RIP) and/or a relaxase protein (REL). These are unambiguous assignations, defined in the inset table (Step 2). One additional potential plasmid is shown by a chromosome-connected network with two replication proteins. It is defined as p5 by the red circle in **Figure S27**. p5 is a 16 contig plasmid, adding a total of 104,416 bp. It is an IncF plasmid, according to its two RIP proteins (yellow-tagged contigs containing IncFrepb and IncFIA in **Figure S27**). Four small contigs (shown in the Blastn descriptions in the inset Table Step 3), corresponding to known hubs, were duplicated. The resulting summary of plasmid definitions is shown in **Table S3**. As shown in the Table, plasmid reconstructions give almost zero error rate for plasmids p1 to p4. The 5.6 kb DNA sequences missing in the PLACNET reconstruction of plasmid p5 correspond to insertion sequences (IS66, ISEC23 and IS26) that were repeated two, two and six times in the original sequence but were considered only once in the reconstructed plasmid. The IS elements appeared after the assembly as either isolated contigs or linked

to one of the unique plasmid sequences. Although these IS elements show additional scaffold links, IS multiplication within the plasmid was not attempted. Nevertheless, as seen in **Figure S28**, PLACNET reconstruction includes essentially all sequences in plasmid p5. Conversely, only two very small contigs (nodes 104 and 120) corresponding to IS911 transposases, are not correctly assigned to plasmid p5, as shown in **Figure S29**. These nodes were hubs, (wrongly) duplicated during network pruning, due to their link to references connecting the chromosome with the plasmid.

Exemplar reconstruction of single-plasmid genomes: *E. coli* ST131 commensal strain SE15 and UPEC strain UTI89

The reconstruction of strain SE15 genome is summarized in **Figures S30** to **S33**. **Figure S30** shows the transition from the original to the pruned SE15 network, underscoring the presence of one RIP and one REL protein (color-tagged contigs). **Figure S31** helps interpreting the network. Three nodes, loosely bound to the chromosome, correspond to chromosomal sequences, as inferred from nearest BLAST hits (see the Blastn descriptions of the red background nodes in Inset Table Step 2). Three other "hub" nodes, which were duplicated, are also indicated with a green circle in the network and described in Inset table Step 2. The IncF plasmid was reconstructed from 15 contigs, as shown in **Figure S32**. As shown in the figure, there are only three differences (totaling 5,709 bp) between plasmid pECSF1 and the reconstructed plasmid. They correspond to three copies of IS66 (two complete copies plus one incomplete copy of the 2.436 bp element), which is also contained in the main chromosome. No node was wrongly assigned to the plasmid (data not shown).

The reconstruction of strain UTI89 genome was straight forward. Since it presented no special problems, the detailed steps of the reconstruction are not shown. The results are summarized in **Table S3**. As can be seen, the IncF plasmid is almost perfectly reconstructed, with just 0.5% error rate.

Additional plasmid reconstructions, underscoring main PLACNET reconstruction problems

Seven other *E. coli* genomes were reconstructed to validate PLACNET as well as to outline potential problems in plasmid reconstruction. A summary of the results is shown in **Table S3**. In general, reconstruction of small plasmids is almost perfect most of the times, with error rates <1%. Genomes containing large plasmids are also straightforwardly reconstructed if the corresponding genome contains a single large plasmid, even in the presence of co-resident small plasmids, as shown by the reconstruction of the genome of strain SMS-3-5 (**Table S3**) and that of the previously discussed strain JJ1186. In most cases, the error rate for large plasmids is <5%. Genomes with one large plasmid (with or without small plasmids) were the most commonly encountered situation in the set of 68 sequenced *E. coli* genomes as of August 2014.

Nevertheless, more complicated situations were sometimes found. The next two genomes in **Table S3** (corresponding to strain MG1655 carrying either the recombinant plasmid pEC_L46 or the two separate plasmids pEC958 and R46) illustrate PLACNET discrimination in the reconstruction of a plasmid

cointegrate with respect to the same strain containing the plasmids that originated the cointegrate as two separate genetic entities. In this particular case, the cointegrate is nicely reconstructed with 3% error rate. On the other hand, the reconstruction of the 2-plasmid strain is more problematic. The IncN plasmid cannot be fully reconstructed (resulting in a 19% error) by the lack of assignment of a five contig set (containing sequences of the In1 integron) with the IncN backbone. This problem is represented in the Cytoscape network shown in **Figure S33**. The In1 element (5 contig element circled in blue) should belong to one or the other plasmid in the strain, but this cannot be decided by the lack of scaffold links. In this particular case (as in others), relying solely on reference links does not help, since the In1 integron is mobile and thus could be linked to different backbones. This result underscores the crucial importance for the dataset to provide sufficient scaffold links.

The three next genomes (corresponding to pathogenic *E. coli* strains SS17, RM12581 and 11368), are examples of strains containing two or more large plasmids in genome datasets that were highly fragmented in the assembled datasets (500 - 700 contigs; see **Table S2**). In these situations, PLACNET analysis becomes more complicated, since the number of contigs and reference/scaffold links to analyze grow disproportionately with the number of contigs. In such cases, we found it convenient to eliminate contigs >200 bp, which was the default threshold. By taking out additional (small) contigs, we could reconstruct the plasmids, but sometimes incurring in a penalty in the error rate of assignment (up to 15%). Results are shown in **Table S3**. For strain SS17, it was sufficient to eliminate contigs <350 bp. Using this modification, the reconstructed plasmids showed error rates of 1-3%. In the cases of RM12581 and 11368, all contigs <500 bp had to be eliminated for proper plasmid reconstruction, resulting in error rates up to 15%. This result underscores another property of PLACNET: plasmid definition improves by increasing contig size elimination in the pruning step, but this comes at a price in terms of a larger error rate in the reconstructed plasmid.

The last genome in **Table S3** reports the only case we analyzed in which PLACNET failed to separate individual plasmids in an *E. coli* genome. It corresponds to the ETEC strain H0407. The strain contains two IncF plasmids of 66,681 bp and 94,797 bp. These two plasmids show extensive homologous regions, in which DNA identity is >90% (incidentally, they can be compatible, belonging to IncFI and IncFII incompatibility groups, for instance, due to a few point mutations in their replication region). Thus, it is impossible for the Illumina assembling programs to distinguish among many DNA segments that are almost identical in both plasmids. Thus, the Cytoscape representation of these plasmids is a densely connected network, as shown in **Figure S34**. This is because the non-segregated sequences produce scaffold links with contigs belonging to both plasmids. As an example, there is just a single contig containing the REL and RIP proteins of both plasmids (red arrow in **Figure S34**).

Confirmation of plasmid sizes by S1-PFGE.

The use of S1-PFGE allows the visualization of plasmids in a DNA sample as well as the estimation of the molecular weight (Barton et al., 1995). Table S4 shows a summary of the results obtained by S1-PFGE on the four strains (FV9873, E35BA, E2022 and E61BA) that were sequenced for this work. As can be seen in the table, there is a good correlation between the number and molecular size of the plasmids as estimated by S1-PFGE when compared to PLACNET reconstructions. The only significant difference is the

identification of two molecular species of aprox. 140 and 75 kb in the case of S1-PFGE that could not be differentiated by PLACNET reconstruction of strain E35BA. Nevertheless, PLACNET suggested the existence of two plasmid species based on the existance of two different relaxases in this genome. It shoud be also noted that S1-PFGE does not allow the visualization of plasmids of less than 15 kb.

Conclusions

PLACNET correctly identifies plasmids in Illumina-based WGS datasets. With practically no exception, PLACNET identifies and assembles plasmid backbones either in a single contig or as a connected component of several contigs. Plasmids containing multiple contigs may not be covered to 100% of their sequences, sometimes carrying a small error rate in the contigs assigned to each plasmid. The error is very low (<1%) for small plasmids, but can be as high as 20% for genomes containing several large plasmids. There are two main sources of error:

1. Repeated sequences originating from mobile genetic elements (MGEs). They are usually spotted as hubs, because they show multiple scaffold links, and their contigs are hits to known MGEs. Two alternative strategies are used by PLACNET to deal with repeated sequences. They can be eliminated, mainly if the contigs are small, or duplicated. As a result, the network connectivity diminishes and allows the identification of disjoint connected networks, as explained in the text.

2. Plasmids with extensive regions of high homology. This is in fact equivalent to the presence massive repeated sequences. In these cases, the relevant plasmids cannot be separated. They are identified by their signature sequences (REL and/or RIP).

Sources of error are more relevant if:

- A particular genome assembly results in highly fragmented genomes (400 contigs or more). This can be minimized by combining paired-end with mate-pair sequencing.

- The analyzed genome contains many repeated sequences. This is an intrinsic source of error that can only be minimized by improving the quality of the assembly process.

- There is a lack of a sufficiently wide reference dataset. This was clearly not a problem with *E. coli*, but certainly is for other genomes for which no many plasmid sequences are available. Future work will deal with this problem, when we report on plasmid reconstruction for genomes of other bacteria.

Besides, the existence and molecular size of the plasmids reconstructed by PLACNET from the four strains sequenced in our laboratory were confirmed by S1-PFGE. In all cases, but the two IncF plasmids of strain E35BA, which could be not separated by PLACNET, all plasmids were correctly identified and their molecular sizes were calculated with acceptable error rate (less than 3%).

Supplementary reference

Huang, W., Li, L., Myers, J.R., and Marth, G.T. (2012). ART: a next-generation sequencing read simulator. Bioinformatics (Oxford, England) *28*, 593-594.

Valverde, A., Cantón, R., Garcillán-Barcia, M.P., Novais, A., Galán, J.C., Alvarado, A., de la Cruz, F., Baquero, F., Coque, T.M. (2009). Spread of *bla*$_{CTX-M-14}$ is driven mainly by IncK plasmids disseminated among *Escherichia coli* phylogroups A, B1, and D in Spain. Antimicrob Agents Chemother *53(12),* 5201-5212.

Barton, B. M., Harding, G.P., Zuccarelli, A.J. (1995). A general method for detecting and sizing large plasmids. Anal Biochem, *226*, 235-240.
